# Douglas‐fir *LEAFY COTYLEDON1* (*PmLEC1*) is an active transcription factor during zygotic and somatic embryogenesis

**DOI:** 10.1002/pld3.333

**Published:** 2021-07-29

**Authors:** Mariana A. Vetrici, Dmytro P. Yevtushenko, Santosh Misra

**Affiliations:** ^1^ Department of Biological Sciences University of Lethbridge Lethbridge AB Canada; ^2^ Centre for Forest Biology Department of Biochemistry & Microbiology University of Victoria Victoria BC Canada

**Keywords:** Douglas‐fir, conifer *LEC1*, somatic embryogenesis, stress and hormone treatments, transcription factors

## Abstract

Douglas‐fir (*Pseudotsuga menziesii*) is one of the world's premier lumber species and somatic embryogenesis (SE) is the most promising method for rapid propagation of superior tree genotypes. The development and optimization of SE protocols in conifers is hindered by a lack of knowledge of the molecular basis of embryogenesis and limited sequence data. In Arabidopsis, the *LEAFY COTYLEDON1* (*AtLEC1*) gene is a master regulator of embryogenesis that induces SE when expressed ectopically. We isolated the *LEC1* homologue from Douglas‐fir, designated as *PmLEC1*. *PmLEC1* expression in somatic embryos and developing seeds demonstrated a unique, alternating pattern of expression with the highest levels during early stages of embryogenesis. PmLEC1 protein accumulation during seed development correlated with its transcriptional levels during early embryogenesis; however, substantial protein levels persisted until 2 weeks on germination medium. Treatment of mature, stratified seeds with 2,4‐epibrassinolide, sorbitol, mannitol, or NaCl upregulated *PmLEC1* expression, which may provide strategies to induce SE from mature tissues. Sequence analysis of the *PmLEC1* gene revealed a 5′ UTR intron containing binding sites for transcription factors (TFs), such as ABI3, LEC2, FUS3, and AGL15, which are critical regulators of embryogenesis in angiosperms. Regulatory elements for these and other seed‐specific TFs and biotic and abiotic signals were identified within the *PmLEC1* locus. Most importantly, functional analysis of *PmLEC1* showed that it rescued the Arabidopsis *lec1‐1* null mutant and, in the T2 generation, led to the development of embryo‐like structures, indicating a key role of *PmLEC1* in the regulation of embryogenesis.

## Graphical abstract

Ectopic expression of *PmLEC1* leads to the formation of embryo‐like structures in the T2 *lec1‐1* *^PmLEC1^* seedlings.

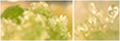

## 1 INTRODUCTION

Douglas‐fir (*Pseudotsuga menziesii* [Mirb.] Franco) is an economically valuable conifer that is native to western North America and cultivated throughout Europe, New Zealand, and Australia for its superior growth performance, desirable wood qualities, and ability to withstand the abiotic stresses of a changing climate (Spiecker, 2019). Douglas‐fir is in high demand, but reforestation and afforestation initiatives are severely strained by its long reproductive cycle (17 months), unreliable seed production, and germination efficiencies as low as 40% (Allen & Owens, 1972).

Somatic embryogenesis (SE), the asexual production of embryos from differentiated tissues, is the most promising method for rapid propagation of large numbers of true‐to‐type, high‐value trees (Gupta & Timmis, 2005; Pullman et al. 2003). In Douglas‐fir, SE begins with the induction of embryogenic calluses from immature zygotic embryos, usually at the precotyledonary stage, via treatment with plant growth regulators (PGRs) 2,4‐dichlorophenoxyacetic acid (2,4‐D), kinetin, and 6‐benzylaminopurine (BA) (Gupta et al., 1991). Dedifferentiated calli proliferate as proembryogenic masses (PEMs), and the embryogenic sequence is recapitulated via subculturing in stage‐specific combinations of PGRs and media to produce singulated, precotyledonary, cotyledonary, and mature embryos (Gupta et al., 1991). Singulation is a process unique to Douglas‐fir SE, whereby the callus separates into individual somatic embryos.

SE ensures consistent and unlimited production of quality embryos irrespective of the time of year and shortens embryo production from 17 months to 4–6 months (Gupta et al., 1991). Forests composed of superior genotypes will yield genetic gains much faster than those that could occur by natural selection (Pullman et al., 2003). However, the molecular regulatory mechanisms that induce SE in conifers remain unknown (Gautier et al., 2019; Pullman et al., 2015). Many conifer genotypes are recalcitrant to SE induction (Pullman et al., 2005). More than 50% of Douglas‐fir SE cultures discontinue growth after 6 months, and significant losses occur during the progression from culture initiation to somatic seedling establishment (Gupta et al., 1991). Moreover, conifer SE can be induced only from juvenile tissues (e.g., megagametophytes, zygotic embryos) that are not readily available throughout the year or when the superiority of a tree is proven.

The paucity of knowledge about the genes and molecular events responsible for embryogenesis in conifers, and the lack of molecular markers of embryogenic potential, have permitted only trial‐and‐error tests of medium components, PGRs and stress treatments for improving SE. Researchers urgently need gene expression profiles to understand natural embryo development (Pullman et al., 2015), and the characterization of the embryogenic state at the molecular level, before the full potential of SE can be realized (Gautier et al., 2019). Research efforts are hampered by limited sequence information due to large conifer genomes (20–40 Gb), lengthy life cycles, the absence of a reliable transformation system to study reverse genetics, and small embryos being buried in maternal tissues (Cairney & Pullman, 2007). Progress in conifer embryogenesis depends on angiosperm model plants combined with rigorous investigation and inference. Angiosperm SE is easily manipulated, and hundreds of genes that function during angiosperm embryogenesis have been characterized. EST studies suggest that 70% of embryo‐specific genes are shared with conifers (Cairney & Pullman, 2007).

In angiosperms, the most prominent transcription factor (TF) with a high‐order regulatory role in embryogenesis is *Arabidopsis* LEAFY COTYLEDON1 (AtLEC1). AtLEC1 is a subunit of a CCAAT box‐binding TF and is also named AtHAP3 or AtNF‐YB9; the NF‐YB family comprises 13 members. *AtLEC1* acts during both early and late embryogenesis and is a central regulator of embryo and endosperm development in Arabidopsis (Lotan et al., 1998). *AtLEC1* promotes epigenetic reprogramming and controls morphogenesis, photosynthesis, and storage compound accumulation by regulating different sets of genes at specific stages of embryogenesis (Jo et al., 2019). *AtLEC1* rescues the Arabidopsis *lec1‐1* null mutant, and its ectopic expression induces embryonic programs and leads to spontaneous formation of embryo‐like structures from vegetative tissues (Lotan et al., 1998).

We hypothesized that Douglas‐fir has its own homologue of the *AtLEC1* gene that plays a critical role in conifer embryogenesis and might be used to induce SE. Hence, the aim of our study was to identify, isolate, and characterize the *LEC1* gene from Douglas‐fir and evaluate its potential as a candidate gene for applications in SE. Phylogenetic analysis of the putative amino acid sequence deduced from *PmLEC1* cDNA revealed that PmLEC1 grouped with the AtLEC1 clade. *PmLEC1* gene expression during seed development demonstrated a unique, alternating pattern, with the highest levels occurring during early embryogenesis. Conversely, PmLEC1 protein accumulation persisted at substantial levels until the germination stage. *PmLEC1* expression was upregulated in mature, stratified seeds by treatment with 2,4‐epibrassinolide, sorbitol, mannitol, or NaCl. Acquisition of the flanking upstream region and sequence analysis of the *PmLEC1* locus revealed a 5′ UTR intron‐containing binding sites for TFs known to interact with *LEC1* and critical to angiosperm embryogenesis. Thus, homologous TFs are expected to participate in conifer embryogenesis. Functional analysis of *PmLEC1* via ectopic expression in the Arabidopsis *lec1‐1* null mutant showed that *PmLEC1* complemented the mutation, generated embryo‐like structures in T2 seedlings, and induced embryonic programs in vegetative tissues. These findings expand our understanding of the molecular biology of conifer embryogenesis and may lead to improved SE protocols for Douglas‐fir and other gymnosperms.

## 2 MATERIALS AND METHODS

### 2.1 Plant material

Douglas‐fir somatic embryos donated by Weyerhaeuser (Seattle, WA, USA) were maintained in vitro as PEM in liquid BM2 media (Gupta et al., 1991). Singulated, stage 3 somatic embryos were generated by subculturing PEM in BM3 media (Gupta et al., 1991). Precotyledonary, cotyledonary, and mature embryos were produced by subculturing singulated embryos on solid BM4 media, followed by harvesting at 20, 30, and 45 days, respectively (Gupta et al., 1991).

Developing seeds, vegetative buds, and pollen cones were collected from open‐pollinated coastal Douglas‐fir trees growing in Saanich, BC, Canada, during May–August, immediately frozen in liquid nitrogen and stored at −80℃.

Douglas‐fir seeds for imbibition, stratification, and germination were obtained from an open‐pollinated seed orchard (Sorrento Nurseries Ltd., Sorrento, BC, Canada). The seeds were imbibed in distilled water with slow agitation for 24 hr at 4℃, then stratified at 4℃ for 3 weeks between layers of Whatman Grade 1 filter paper placed over water‐soaked sponges. To expose stratified seeds to germination conditions, the seeds were placed over Kimpack absorbent cellulose wadding (LPS Industries) cut to the size of 9‐cm Petri dishes, soaked with water and covered with two layers of filter paper. The seeds were placed on top of the filter paper. The petri dishes were sealed with Parafilm (Beemis, Neenah, WI, USA) and placed in a growth chamber under a 16‐hr light photoperiod (16 μmol/m^2^ s^−1^) at 24℃. Germinating seeds were collected after 2, 6, 10, 12, 14, 45, and 90 DAEG, frozen in liquid nitrogen and stored at −80℃ until further analysis.

### 2.2 Primers

PCR primers used in this work were designed by MV and synthesized at Invitrogen and are listed in Table S2.

### 2.3 RNA isolation

Total RNA was isolated from somatic embryos, developing seeds, imbibed seeds, and stratified seeds by the TRIzol method modified for plants (Invitrogen). Isolation of total RNA from seeds exposed to germination conditions, seedlings, vegetative buds, and pollen cones was performed using the modified hot‐phenol extraction (Verwoerd et al., 1987).

### 2.4 Isolation and cloning of the conserved *LEC1* sequence

Total RNA from somatic embryos at the maintenance and singulated stages, and immature zygotic seeds, was used in separate RT‐PCR reactions. Total RNA was treated with Invitrogen Amplification Grade DNase I, and the absence of DNA contamination was confirmed by conventional PCR. First‐strand cDNA synthesis was performed with 5 μg total RNA, 1 μl oligo(dT)_12_VN (V = A or C or G, *N* = A or C or G or T), and SuperScript II RNase H^‐^ reverse transcriptase (Invitrogen).

Degenerate PCR primers based on the conserved sequence of *Arabidopsis thaliana* ecotype WS *AtLEC1*, and an EST sequence of *Pinus taeda,* were designed to amplify a homologous Douglas‐fir sequence from PEM‐derived cDNA. PCR was performed using the QIAGEN Taq PCR Master Mix (Qiagen, Mission, ON, Canada) with the following thermocycle program: 5 min at 94℃, 40 cycles of denaturation (94℃ for 30 s), annealing (60℃ for 30 s), and elongation (72℃ for 1 min), followed by 10 min extension at 72℃. The products were resolved by agarose gel electrophoresis and visualized after ethidium bromide (EtBr) staining.

The single, amplified PCR product (~200 bp) was extracted from the gel using the QIAquick Gel Extraction Kit (Qiagen), ligated into the pCR2.1 TOPO vector, and transformed into *E. coli* using the TOPO TA cloning kit (Invitrogen). Plasmid DNA was purified from transformed colonies using QIAprep Spin Miniprep kit (Qiagen), and the inserted DNA was sequenced at the University of Victoria DNA Sequencing Centre. Database searches for similar sequences were performed using BLAST.

### 2.5 Northern blot analysis

RNA (20 μg) was resolved by denaturing formaldehyde gel electrophoresis and checked for equal loading and integrity by visualizing after ethidium bromide staining. The RNA was transferred to a Biodyne B nylon membrane and hybridized with a ^32^P‐labeled *PmLEC1* cDNA probe.

### 2.6 RACE‐PCR for obtaining full‐length cDNA

To isolate sequences upstream and downstream of the conserved domain, RACE‐PCR was performed with 1 μg total RNA isolated from ~200 mg singulated somatic embryo masses harvested from liquid medium. The SMART RACE cDNA Amplification Kit (BD Biosciences) was used according to the manufacturer's instructions. The 5′‐ and 3′‐RACE‐PCR products were resolved in 1% agarose gels, purified, cloned, and sequenced, as described above. A new set of primers was designed based on the start and stop codons of individual RACE‐PCR sequences. RT‐PCR was performed with RNA isolated from zygotic developing seeds. The product was resolved and visualized on an agarose gel, extracted from the gel, cloned, and sequenced, as described above.

### 2.7 Phylogenetic analysis

The full‐length *PmLEC1* sequence was queried against the TBLASTX database (Altschul et al., 1990). Full‐length LEC1 or LEC1‐like putative protein sequences were aligned with MUSCLE (http://www.ebi.ac.uk/Tools). Phylogenetic tree construction was performed by importing the MUSCLE alignment into MEGA X (Kumar et al., 2018) and applying the neighbor joining method and bootstrapping with 1,000 replicates.

### 2.8 Real‐time quantitative reverse transcription PCR (qRT‐PCR) analyses

First‐strand cDNA synthesis was performed with 1 μg DNase I‐treated RNA, 1 μl oligo(dT)_12_VN primer and Invitrogen SuperScript II RNase H^‐^ Reverse Transcriptase in 20‐μl reactions of five biological replicates. The cDNA was diluted 20‐fold, and 2 μl of the dilution was used in 14‐μl reactions with Invitrogen Platinum SYBR Green qPCR SuperMix‐UDG, performed in quadruplicate (4 technical replicates), in a Stratagene Mx4000 real‐time thermocycler (Stratagene). Amplification program: 9‐min enzyme activation at 94℃, and 40 cycles of denaturation (94℃ for 15 s), annealing (60℃ for 30 s), and elongation (72℃ for 45 s). Reaction specificity was confirmed with melting curve analysis. Data were normalized to the expression of the invariant ribosomal protein L8 gene, and relative expression was calculated using the 2^−ΔΔCt^ method. Statistical analyses were performed with SPSS (SPSS Version 12.0) using Kruskal–Wallis and two‐tailed Mann–Whitney *U* tests for nonparametric data.

### 2.9 Treatment of stratified seeds with stress factors and plant growth regulators; qRT‐PCR

Stratified seeds were placed in petri dishes on filter paper soaked with each treatment compound and placed in the dark at 24℃ for 24 hr. The treatment solutions were 0.7 M sorbitol, 0.7 M mannitol, 0.7 M sucrose, 0.3 M NaCl, 0.6 mM CdCl_2_, 23.75 mM PEG 8000, 10 μM 2,4‐epibrassinolide, 50 μM 2,4‐D in combination with 20 μM BAP, and 7.2 μM GA_3_ in combination with 38 μM ABA. For the hypotonic treatment, seeds were fully immersed in water. Water‐moistened filter paper served as the control treatment. Following treatment, seeds were excised from the seed coats, immediately frozen in liquid nitrogen and stored at −80℃ until RNA isolation. Each treatment group consisted of three seeds (three biological replicates). RNA was isolated separately from each seed and used in quadruplicate qRT‐PCR reactions (four technical replicates) with the ribosomal protein L8 gene as the normalizer. qRT‐PCR and statistical analyses were performed as described above.

### 2.10 Antibody production

A peptide corresponding to the first 18 amino acids of the putative PmLEC1 protein, with an additional cysteine residue at the C‐terminus (MMSEVGSPTSQDSRNSEDC) and coupled to the KLH carrier protein was synthesized by GenScript Corporation. Antibody production was performed at Immuno‐Precise Antibodies, Ltd. Four Balb/C mice were each immunized with 25 μg of the KLH‐coupled peptide and mixed with Freund's complete adjuvant. Six additional immune boosts of 25 μg peptide‐KLH in Freund's incomplete adjuvant followed at 3‐week intervals. Dilutions of the polyclonal mouse antiserum were tested by ELISA against the free peptide and protein extracts from Douglas‐fir developing seed. The polyclonal antiserum from two mice showed a significant response against the peptide when used at a dilution of 1:1,000. Blood was drawn from these two mice on four dates over a 2‐month period. The antisera were obtained by centrifugation and combined for a total of 6 ml of polyclonal antiserum used in western blotting.

### 2.11 Western blot analysis

Total proteins were extracted from frozen and ground developing seeds, imbibed seeds, stratified seeds, seeds exposed to germination conditions, vegetative buds and pollen cones, and suspended in extraction buffer (65 mM Tris (pH 6.8), 1% SDS, 5% glycerol and 2.5% EtSH) at 1 mg/3 μl. Protein concentrations were determined by the Bradford assay (Bradford, 1976).

Total proteins (20 μg) were resolved by SDS‐PAGE and transferred to PVDF membranes (Amersham Biosciences). Equal protein loading was confirmed by staining duplicate gels with Coomassie brilliant blue. Western blotting was performed with mouse anti‐PmLEC1 primary antibody (Immuno‐Precise Antibodies) diluted 1:1,000, and ImmunoPure Goat Anti‐Mouse IgG, Peroxidase Conjugated (Pierce Biotechnology) secondary antibody diluted 1:100,000. Proteins were detected by chemiluminescence with ECL Plus Western Blotting Detection Reagents (Amersham).

### 2.12 Isolation of the genomic sequence upstream of the coding region

Total DNA was isolated from somatic embryos using the Sigma GenElute Plant Genomic DNA kit (Sigma) and from Douglas‐fir needles using the modified cetyltrimethylammonium bromide method (De Verno et al., 1989).

The Genome Walker Universal Kit (Clontech) was used with Douglas‐fir genomic DNA to create four genomic libraries corresponding to digestion by restriction enzymes *Dra* I, *Eco* RV, *Pvu* II, and *Stu* I. PCR reactions were performed using QIAgen Taq PCR Master Mix (Qiagen). Only the *Dra* I and *Pvu* II libraries yielded specific, major products in the secondary PCR reaction. These two DNA products were purified from agarose gels, cloned, and sequenced as described above. The sequences were identical where they overlapped, but the sequence from the *Dra* I library was longer.

### 2.13 Sequence analysis for cis‐ and trans‐acting regulatory elements

The *Dra* I sequence, GenBank accession number FJ418169, provided the longest nucleotide sequence upstream of the coding region. Sequence analysis was completed using the Plant Promoter Analysis Navigator (PlantPan) 3.0 program (http://plantpan.itps.ncku.edu.tw; Chow et al., 2019).

### 2.14 Arabidopsis plant material and generation of transgenic plants

Wild‐type and heterozygous *LEC1/lec1* seeds of *A. thaliana* (L.) Heynh ecotype Wassilewskija, stock numbers CS2360 and CS8101, respectively, were obtained from the Arabidopsis Biological Resource Center. Homozygous *lec1‐1* plants were generated by planting the heterozygous seeds and growing them in a greenhouse under standard conditions. Plants self‐pollinated to produce homozygous progeny. Immature seeds were removed from green siliques and cultured on half‐strength MS medium (Murashige & Skoog, 1962). Only homozygous *lec1‐1* seeds germinate under these conditions. The phenotype was confirmed by PCR analysis using *AtLEC1* gene‐specific primers.

Two expression cassettes were constructed, one containing the *PmLEC1* gene and another containing the *AtLEC1* gene. The *PmLEC1* and *AtLEC1* coding sequences were directionally inserted into the corresponding restriction sites of the pBI221 vector (Jefferson et al., 1987) between the cauliflower mosaic virus duplicated enhancer and the alfalfa mosaic virus untranslated leader sequence (35S‐35S‐AMV) promoter (Datla et al., 1993), and the NOS terminator. Each vector contained the *NPTII* kanamycin‐resistance gene for plant selection. The vectors were transferred into the *Agrobacterium tumefaciens* strain M90 as described by Datla et al. (1993). The transformed colonies were selected on antibiotic‐containing medium, and the presence of the insert was confirmed by sequencing.

Arabidopsis *lec1‐1* null mutant and wild‐type plants were transformed using the floral dip method (Clough & Bent, 1998). Flowering plants (T0 generation) were dipped into *Agrobacterium* suspension and produced the first generation of transgenic seeds (T1). Transgenic T1 plants were selected on kanamycin‐containing MS medium, and transgene integration was confirmed by PCR. T1 plants were grown in a greenhouse and self‐pollinated to produce T2 seeds. T2 plants were selected on kanamycin‐MS medium, genotyped, and used in gene expression studies.

### 2.15 Accession numbers

FJ418168 (*P. menziesii LEC1*), AEF56565.2 (*Larix decidua* LEC1), AEG75669.1 (*Picea abies* HAP‐3), ADR10435.1 (*Pinus contorta* HAP‐3), AEG75670.1 (*Pinus sylvestris* HAP‐3), CAI05932.1 (*Helianthus* *annuus* L1L), CAM35799.1 (*T. cacao* L1L), XP_017980261.1 (*Theobroma cacao* NF‐YB9), NP_001236625.1 (*Glycine max* LEC1‐A), ACB12186.1 (*Brassica napus* LEC1), CRY49357.1 (*Coffea canephora* LEC1), XP_016687892.1 (*Gossypium hirsutum* NF‐YB6), XP_016705377.1 (*G. hirsutum* NF‐YB‐9‐like), BAD15083.1 (*Daucus carota* HAP3), ABG67973.1 (*Kalanchoe daigremontiana* L1L), KM115581.1_1 (*Rosa canina* LEC1‐B), RWR83382.1 (*Cinnamomum micranthum* f. kanehirae LEC1), AY138461.1 (*A. thaliana* L1L), NP_001078727.1 (*A. thaliana* NF‐YB6), AAP22065.1 (*Oryza sativa* Indica LEC1), NP_001105518.1 (*Zea mays* LEC1), OAP15884.1 (*A. thaliana* ecotype Columbia LEC1), AAC39488.1 (*A. thaliana* ecotype Wassilewskija LEC1), AZQ19317.1 (*Populus tormentosa* NF‐YB6), XP_024442979.1 (*Populus trichocarpa* NF‐YB6), AJP62202.1 (*Arachis hypogaea* LEC1‐A), NP_001239679.1 (*G. max* NF‐YB6), AAN01148.1 (*Phaseolus coccineus* L1L), XP_013728330.1 (*B. napus* NF‐YB6).

## 3 RESULTS

### 3.1 Isolation of cDNA, phylogenetic analysis and MUSCLE alignment confirm *PmLEC1* identity

A partial cDNA sequence was amplified from Douglas‐fir somatic embryo RNA using degenerate PCR primers complementary to the central, conserved B domain of the Arabidopsis *LEC1*, and an expressed sequence tag (EST) sequence from *P. taeda*. Sequence data were obtained from stages 2 and 3 of Douglas‐fir somatic embryos using multiple combinations of degenerate primers. These reactions yielded a single amplicon, despite the use of degenerate primers and the possibility of a gene family. The amplified sequence was used to design primers for RACE‐PCR to generate the full‐length cDNA from Douglas‐fir singulated somatic embryos. The 779 bp Douglas‐fir cDNA sequence, designated *PmLEC1,* contained 5′ and 3′ UTRs, a start codon, and an open‐reading frame with a single‐stop codon. The 5′ UTR contained a stop codon but no start codons. The absence of start codons within the 5′ UTR correlates with strong translational efficiency (Rogozin et al., 2001).

**FIGURE 1:**
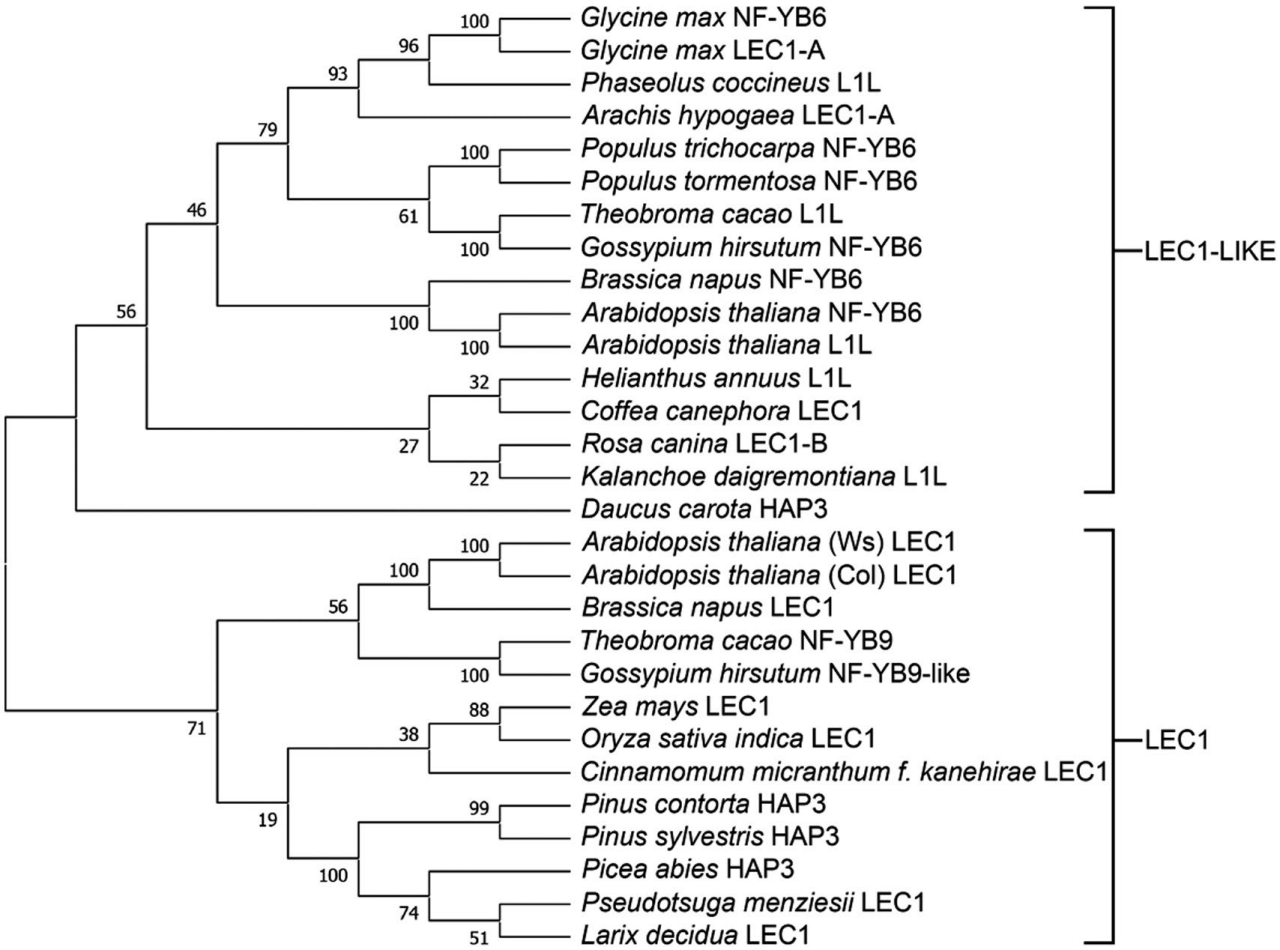
PmLEC1 is more closely related to AtLEC1 than to AtL1L. Evolutionary relationships among LEC1‐type sequences deduced from crop and tree species were determined via alignment in the MUSCLE program and the neighbour‐joining tree method in MEGA X. AtLEC1 (NF‐YB9) and AtL1L (NF‐YB6) each have two sequences available in GenBank. The *A. thaliana* Wassilewskija ecotype has a LEC1 sequence of 208 amino acids while the Colombia ecotype has a 238 amino acid LEC1 sequence. The two published sequences of AtL1L comprise 234 and 205 amino acids. Bootstrap values were obtained from 1,000 replications. The conifer sequences grouped within the LEC1 clade

In Arabidopsis, two of the NF‐YB genes, *LEC1* and *LEC1‐LIKE* *(L1L* or *NF‐YB6)*, are active during embryogenesis and share significant sequence identity within the central, conserved B domain, including the specific residues that are necessary for activity in embryogenesis (Lee et al., 2003). *L1L* is expressed in all vegetative organs, and its expression peaks later in embryogenesis (Kwong et al., 2003). The putative PmLEC1 amino acid sequence showed 55% identity and 71% similarity with AtLEC1, and 53% identity and 70% similarity with AtL1L. Phylogenetic analysis found PmLEC1 to be more closely related to AtLEC1 than to AtL1L, and the conifer sequences grouped within the LEC1 clade (Figure 1).

**FIGURE 2:**
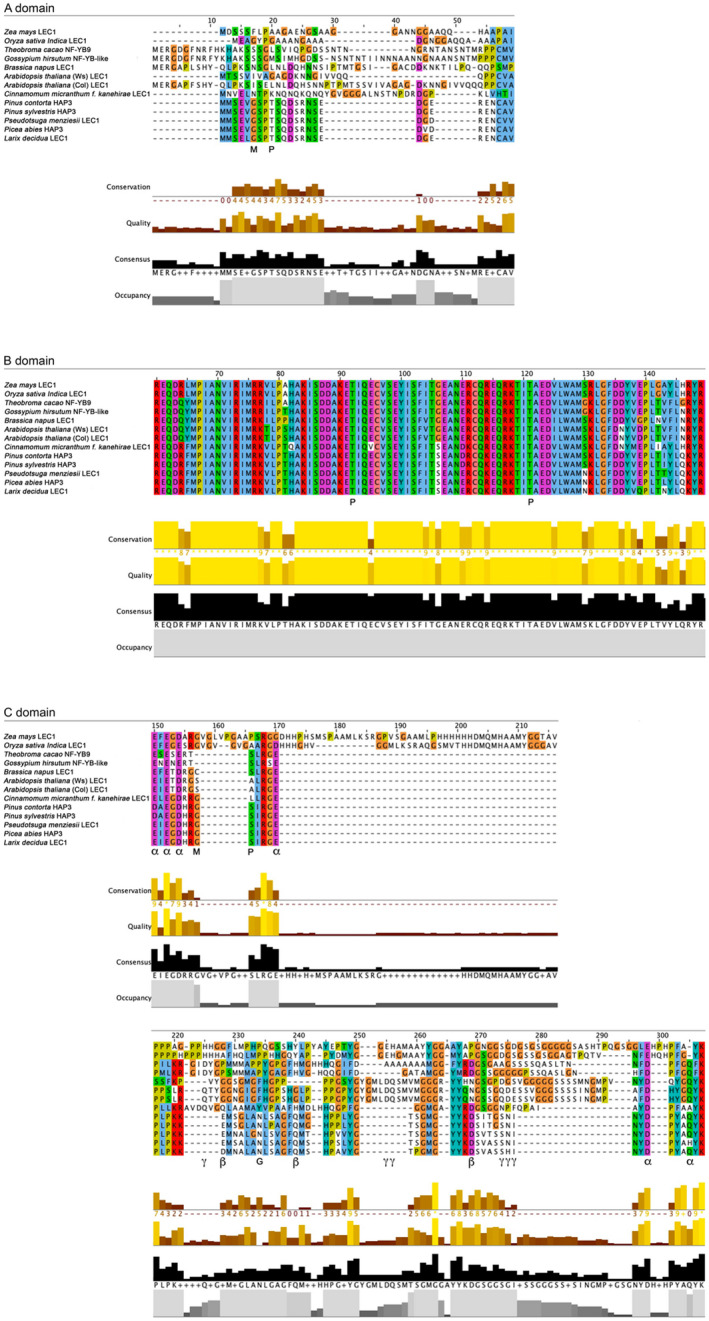
MUSCLE alignment of deduced LEC1 sequences highlights the conservation of the B domain, residues important for protein‐protein interactions in the C domain and potential post‐translational modification sites. α: Gln, Asp and Glu residues conserved between conifers and Arabidopsis. β: Gln, Asp and Glu residues present in conifers but not Arabidopsis. γ: Gln, Asp and Glu residues present in Arabidopsis but not conifers. G: N‐glycosylation site. M: N‐myristoylation site. P: phosphorylation site

Examination of sequence features and critical residues shed further insight into PmLEC1 function (Figure 2). Angiosperm LEC1 contains three domains: A, B, and C. The B domain is conserved across plant species and is critical to embryogenesis. The AtLEC1 B‐domain Asp‐55 residue required for LEC1 activity in embryogenesis (Lee et al., 2003) was found in PmLEC1 and all aligned sequences (Figure 2). In the C domain, Gln, Asp, and Glu residues are critical for protein–protein interactions and have roles in development (Li et al. 1992). Comparison of the C domain of PmLEC1 with those of AtLEC1 and AtL1L revealed that PmLEC1 shares 41% identity with AtLEC1 but only 19% with AtL1L. MUSCLE (Edgar, 2004) alignment of the LEC1 clade revealed six Gln, Asp, or Glu residues that are conserved between conifers and Arabidopsis (α), and three that are unique to conifers (β) (Figure 2). In the Arabidopsis C‐domain, there were three segments of one, two, and three residues that were absent in conifers (γ) (Figure 2).

In silico translation of the *PmLEC1* cDNA sequence on the Expasy server (Gasteiger et al. 2005) resulted in a putative protein of 180 amino acids, with a predicted size of 21 kD. This tool estimates the MW of a protein based on the average isotopic masses of amino acids but does not account for posttranslational modifications (Gasteiger et al. 2005). ScanProsite (DeCastro et al. 2006) analysis of the deduced PmLEC1 sequence revealed four phosphorylation sites (P), two N‐myristoylation sites (M), and a single N‐glycosylation site (G), which were present in all putative conifer LEC1 sequences (Figure 2). Two of the phosphorylation sites were in the central B domain and conserved in all species. The potential modifications within the A and C domains were conserved in conifers but absent in Arabidopsis.

### 3.2 *PmLEC1* expression alternates between high and low levels during zygotic embryogenesis

**FIGURE 3:**
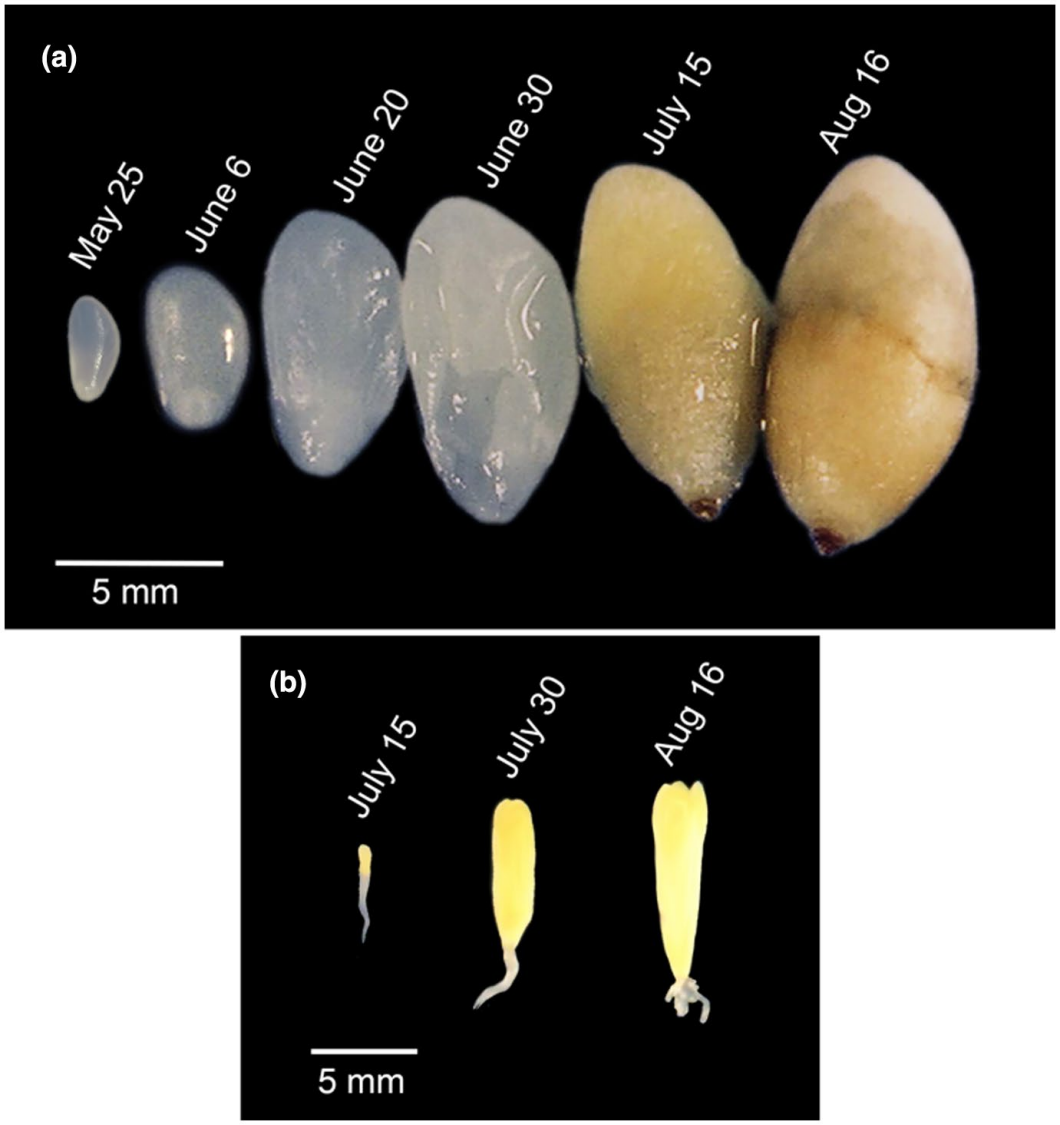
Douglas‐fir developing seeds with seed coat removed, and representative zygotic embryos. A, Seeds of coastal Douglas‐fir collected from open‐pollinated trees and with their seed coats removed exhibit increasing hydrophobicity and size. B, Zygotic embryos excised from corresponding seeds reveal a club‐shaped embryo (July 15), elongating cotyledons (July 30) and a mature embryo with fully elongated cotyledons (Aug 16)

**FIGURE 4:**
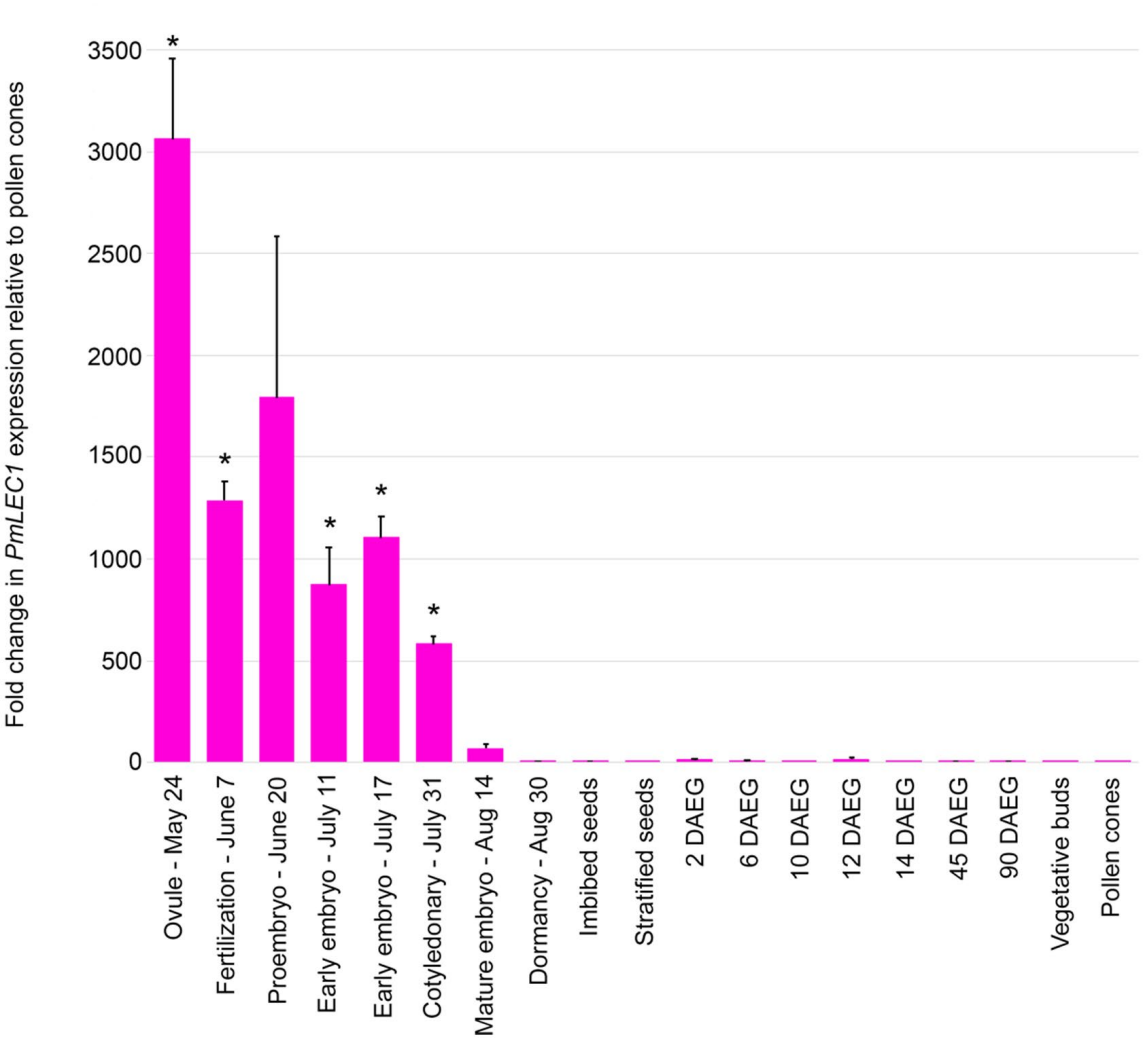
qRT‐PCR analysis of *PmLEC1* expression in Douglas‐fir embryonic and vegetative tissues. Total RNA was isolated from 5 biological replicates at each developmental stage. DNase I treatment and reverse transcription were carried out with 5 samples of 1 μg from each stage. The qRT‐PCR reactions were performed in quadruplicate with Douglas‐fir ribosomal *L8* transcripts as internal controls. Relative gene expression was calculated according to the 2^‐ΔΔCt^ method (Dorak, 2006) by comparison to transcript levels in pollen cones, which showed the lowest expression. Statistical analysis was performed on the pooled groups by the Mann‐Whitney *U* test. Results are shown as means ± SE (*n* = 5). Asterisks indicate *PmLEC1* transcript levels that are significantly different (*p* < .05) from pollen cones. DAEG: days after exposure to germination conditions

To accurately quantify the expression levels of the *PmLEC1* gene, qRT‐PCR analysis was performed using RNA isolated from tissues representing each stage of zygotic embryogenesis (ZE), mature seeds, seeds exposed to germination conditions, vegetative buds, and pollen cones (Figures 3 and 4). The highest levels of expression occurred during seed development, but trace amounts were observed in juvenile tissues, such as seeds exposed to germination conditions, vegetative buds, and pollen cones (Figure 4). A striking alternating pattern of *PmLEC1* induction and repression was noted during seed development, May 24 ‐ Aug 30 (Figure 4). The unfertilized ovule (May 24) showed the highest level of *PmLEC1* expression, demonstrating its activity prior to embryo formation. During fertilization (June 7), there appeared to be a repression of *PmLEC1*, followed by induction at the pro‐embryo stage (June 20) (Figure 4). Another cycle of induction, repression, and induction was visible at the early embryo stage (July 11), early embryo stage (July 17), and cotyledonary stage (July 31), respectively (Figure 4.) The beginning of the mature embryo stage, starting on August 14, was the last time that *PmLEC1* expression was substantial (Figure 4). This marked the end of maturation and the beginning of dormancy, at which time the expression level of *PmLEC1* decreased by two orders of magnitude (Figure 4). From August 30 onward, *PmLEC1* expression was three orders of magnitude lower than during early embryogenesis. In tissues of increasing maturity, including imbibed, stratified, and germinating seeds; vegetative buds; and pollen cones, *PmLEC1* expression was extremely low or undetectable (Figure 4). Trace amounts of *PmLEC1* transcripts were detected in 1.5‐month‐old seedlings, the youngest pollen cones and vegetative buds. No *PmLEC1* expression was observed in 3‐month‐old seedlings, some in 1.5‐month‐old seedlings, pollen cones >2 mm in diameter or vegetative buds >3 mm in diameter.

**FIGURE 5:**
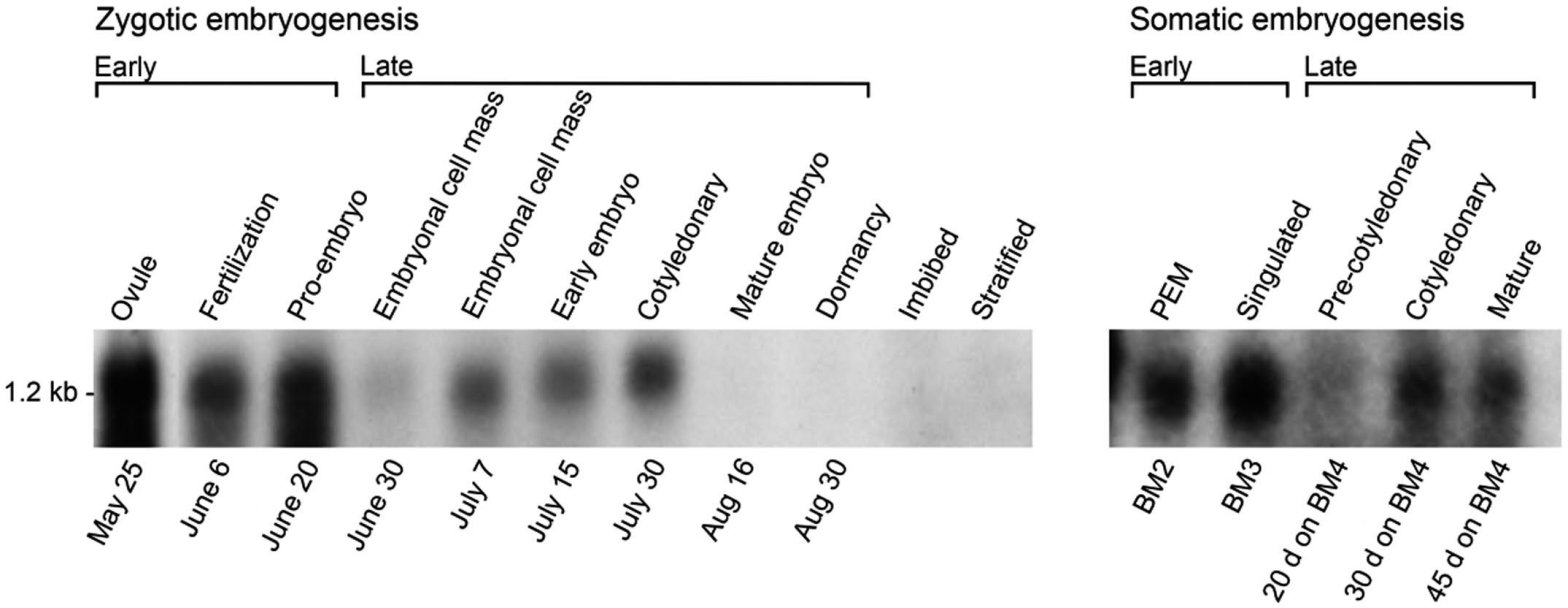
Northern blot analysis of *PmLEC1* gene expression demonstrates an alternating pattern of induction and repression during both ZE and SE. Total RNA samples (20 μg each) were resolved by denaturing formaldehyde‐agarose gel electrophoresis, blotted onto nylon membranes and hybridized with ^32^P‐labeled *PmLEC1* cDNA. A single band was visible at approximately 1.2 kb. Labels above the blots indicate the stage of development. Labels below the blots represent dates of seed collection during ZE, and the media and timing for generating somatic embryos during SE. PEM: proembryogenic masses. BM4: culture media in which somatic embryos reach the indicated stage after the specified number of days

This alternating pattern of *PmLEC1* gene expression was also observed in Northern blot analysis of samples collected in a different year. The alternating pattern and peaks of expression were consistent across both methods and years. Unfertilized ovules (May 25) had the highest expression of *PmLEC1* (Figure 5). Again, an alternating pattern of induction and repression was observed until August 16. Northern blot analyses showed high levels of *PmLEC1* expression during early embryogenesis and lower levels during late embryogenesis during both ZE and SE (Figure 5). *PmLEC1* transcripts were not detectable by northern blot analysis in desiccated, mature embryos (Aug. 16 and 30), in imbibed and stratified seeds (Figure 5), or in vegetative tissues, such as seedling roots, stems, or needles (not shown).

Northern blot analysis also revealed the similarity between ZE and SE (Figure 5): moderate expression in early ZE (fertilization) and PEMs of SE; high expression in proembryos of ZE and singulated somatic embryos; low expression in the precotyledonary stages of both ZE and SE; and moderate expression in the cotyledonary stages of both ZE and SE.

### 3.3 Evaluation of anti‐PmLEC1 polyclonal antiserum in Douglas‐fir seeds and transgenic Arabidopsis plants suggests posttranslational modifications of PmLEC1 during seed development

**FIGURE 6:**
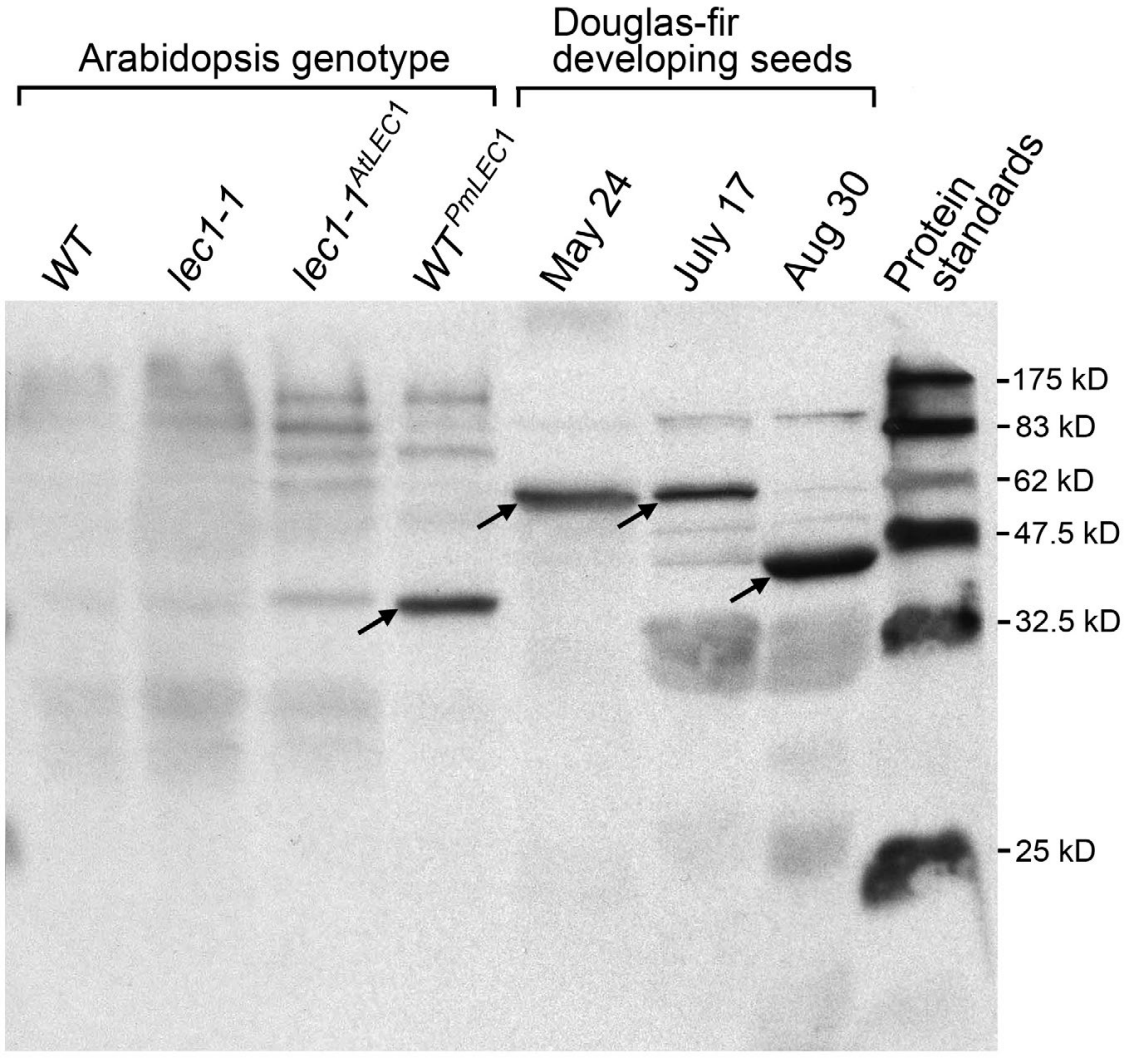
Evaluation of the polyclonal anti‐PmLEC1 antiserum for specificity and cross‐reactivity. Transgenic *lec1‐1^AtLEC1^* and *WT^PmLEC1^* plants were generated via the floral dip method and grown to T2 generation. Total protein extracts (20 μg each) from leaf and stem tissues of *A. thaliana* transgenic and wild type plants, as well as from developing seeds harvested from Douglas‐fir trees at the indicated dates, were resolved by SDS‐PAGE, transferred to PVDF membranes and detected with anti‐PmLEC1 antiserum. The arrows indicate PmLEC1‐specific bands observed in lanes of *WT^PmLEC1^* plants at ~34 kD, and in Douglas‐fir developing seeds at ~36 kD and ~59 kD. WT: Arabidopsis wild type plant. lec1‐1: Arabidopsis *lec1‐1* null mutant. *lec1‐1^AtLEC1^*: Arabidopsis *lec1‐1* mutant transformed with *AtLEC1*. *WT^PmLEC1^*: Arabidopsis *lec1‐1* mutant transformed with *PmLEC1*

To analyze PmLEC1 protein accumulation, we raised antibodies against a synthetic peptide corresponding to the first 18 amino acids of the PmLEC1 N‐terminus (MMSEVGSPTSQDSRNSED). This sequence is well conserved at the N‐terminus of conifer LEC1 sequences, only shares five identical residues within the N‐terminus of AtLEC1 and does not encroach on the evolutionarily conserved B‐domain of NF‐YB (HAP3) proteins. The known, angiosperm NF‐YB family of genes does not share sequence identity withing the N‐terminal A domain. Rather, the NF‐YB family is defined by high conservation within the central B‐domain. Thus, antibodies designed to recognize the N‐terminus of PmLEC1 reduce the chances of other NF‐YB members being detected. A BLAST search using this peptide sequence did not reveal any similarity to other plant sequences. The polyclonal antiserum selected for western blot analysis showed significant responses by ELISA against both the synthetic peptide and cellular proteins extracted from Douglas‐fir developing seed. To validate the antiserum for western blotting, the *PmLEC1* and *AtLEC1* genes were cloned under control of a strong constitutive promoter into Arabidopsis wild‐type and *lec1‐1* null mutant plants, and total proteins were extracted from leaf and stem tissues of the transgenic plants. Western blot analysis of total proteins extracted from *A. thaliana* wild type (WT), *lec1‐1* null mutant, transgenic plants harboring either *PmLEC1* or *AtLEC1* transgenes and developing Douglas‐fir seeds, suggested that the anti‐PmLEC1 antiserum was specific for PmLEC1 and did not cross‐react with AtLEC1 (Figure 6, Figure S1). The faint, nonspecific bands seen in the vicinity of 34 kD in WT, *lec1‐1*, and *lec1‐1^AtLEC1^* are not likely to be other NF‐YB proteins because the antibodies were made against the N‐terminal sequence and the other NF‐YB proteins have a range of different sizes, most of them significantly below 200 amino acids. Moreover, the Coomassie blue‐stained gels indicate the considerable quantity of proteins that were blotted, and this may contribute to nonspecific binding.

In vegetative tissues of *WT^PmLEC1^* plants, the PmLEC1 protein appears to have a molecular weight (MW) of about 34 kD. Developing Douglas‐fir seeds show two distinct PmLEC1 MW species, approximately 36 kD and 59 kD, with a transition between them visible on July 17 (Figure 6 and Figure S1). The Expasy server‐predicted MW for the PmLEC1 protein without modifications is 21 kD (Gasteiger et al., 2005); however, seven posttranslational modifications were predicted via ScanProsite (DeCastro et al. 2006) PmLEC1 sequence analysis. The posttranslational modifications appear to correlate with changes in seed environment from watery (May 25–June 30) to hydrophobic and desiccated (July 15–Aug 16) during embryogenesis (Figure 3a). These changes may be specific for the seed environment and necessary for stabilizing PmLEC1, whereas PmLEC1 protein production within transgenic Arabidopsis stem and leaf tissues would not require the same type of modification (Figure 6 and Figure S1).

### 3.4 PmLEC1 protein persists at substantial levels until germination

**FIGURE 7:**
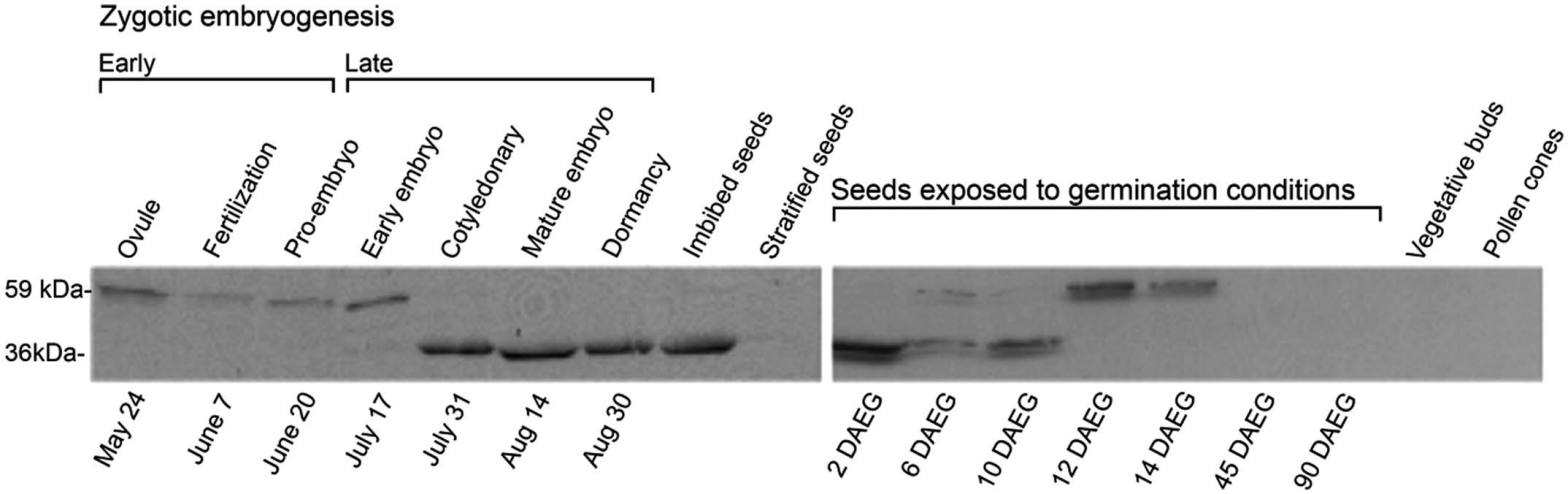
Western blot analysis of PmLEC1 protein during embryonic and vegetative growth reveals a ~59 kD form during early embryogenesis and late germination, and a ~36 kD form during late embryogenesis and early germination. Total protein was extracted from developing seeds harvested from Douglas‐fir trees at the indicated dates and ZE stages, mature imbibed seeds, mature stratified seeds, mature seeds incubated on germination medium for 2, 6, 10, 12, 14, 45 and 90 days, as well as from vegetative buds and pollen cones. The protein extracts (20 μg each) were resolved by SDS‐PAGE, transferred to a PVDF membrane and probed with PmLEC1‐specific polyclonal antibodies. DAEG: days after exposure to germination conditions

To define the temporal PmLEC1 protein distribution and determine the relationship between transcript and protein levels, we performed western blot analysis on protein extracts from all stages of ZE; imbibed, stratified, and germinating seeds; 1.5‐ and 3‐month‐old seedlings; vegetative buds; and pollen cones. During early embryo development and late germination, the antiserum reacted with a 59‐kD molecular species (Figure 7, Figure S1). A transition to 36 kD was apparent on July 17 (Figure 7, Figure S1), with three species being recognized. The 36‐kD MW form was recognized starting with late embryogenesis and until 10 days after exposure to germination conditions (DAEG); there were no reactive bands in the stratified seed samples (Figure 7). At 12 and 14 DAEG, the 59 kD species was again detected. No protein accumulation was observed in 1.5‐ and 3‐month‐old seedlings, vegetative buds, or pollen cones (Figure 7). In conclusion, the PmLEC1 protein appears to be abundant during all phases of embryogenesis (morphogenesis, maturation, desiccation, and germination), with a possible change in MW during the time of seed dormancy (when storage protein accumulation and desiccation take place).

Accumulation of PmLEC1 protein correlated with mRNA levels during early embryogenesis: high, low, and high levels on May 24, June 7, and June 20, respectively, of both protein (Figure 7) and RNA (Figure 5). During late embryogenesis, from July 31 until seed imbibition, protein accumulation remained at similarly high levels (Figure 7). No protein was detected in stratified seeds. Stratification is a process whereby cold and moist conditions encourage seed germination when the ambient temperature changes from 4°C to 24°C. Imbibed seeds, stratified seeds, and seeds exposed to germination conditions had very low levels of *PmLEC1* transcripts (Figures 4 and 5), whereas protein levels at these stages oscillated from high to low and also changed in apparent MW (Figure 7). Evidence exists for intron‐mediated enhancement (IME) affecting translation more profoundly than transcription (Laxa, 2017). Protein levels also appeared to cycle between high and low levels from 2 to 14 DAEG and shifted to the 59 kD form at 12 DAEG (Figure 7). In contrast, beginning on July 11, the RNA levels exhibited another cycle of repression, induction, and repression and then gradually declined to undetectable levels by August 30 (Figure 4). The protein–RNA discordance at the later stages of seed development may be explained by the need to stabilize PmLEC1 during desiccation and seed dormancy to allow it to perform its functions.

### 3.5 Sorbitol, mannitol, NaCl, and brassinosteroid treatments induce *PmLEC1* expression in stratified seeds

In conifers, embryogenic competence is restricted to specific tissues and genotypes, and SE is most easily induced from immature or cotyledonary zygotic embryos, both of which exhibit high *PmLEC1* expression (Figures 4 and 5). Tissues of increasing maturity, such as harvested seeds or vegetative tissues, are progressively more recalcitrant to SE, which is a major obstacle to the commercialization of SE technology. In plants, SE induction is facilitated by 2,4‐D/BA, gibberellic acid/abscisic acid (GA_3_/ABA), brassinosteroids, plasmolysing stressors (sorbitol, mannitol, or sucrose) and other stressors such as heavy metals or salt.

**FIGURE 8:**
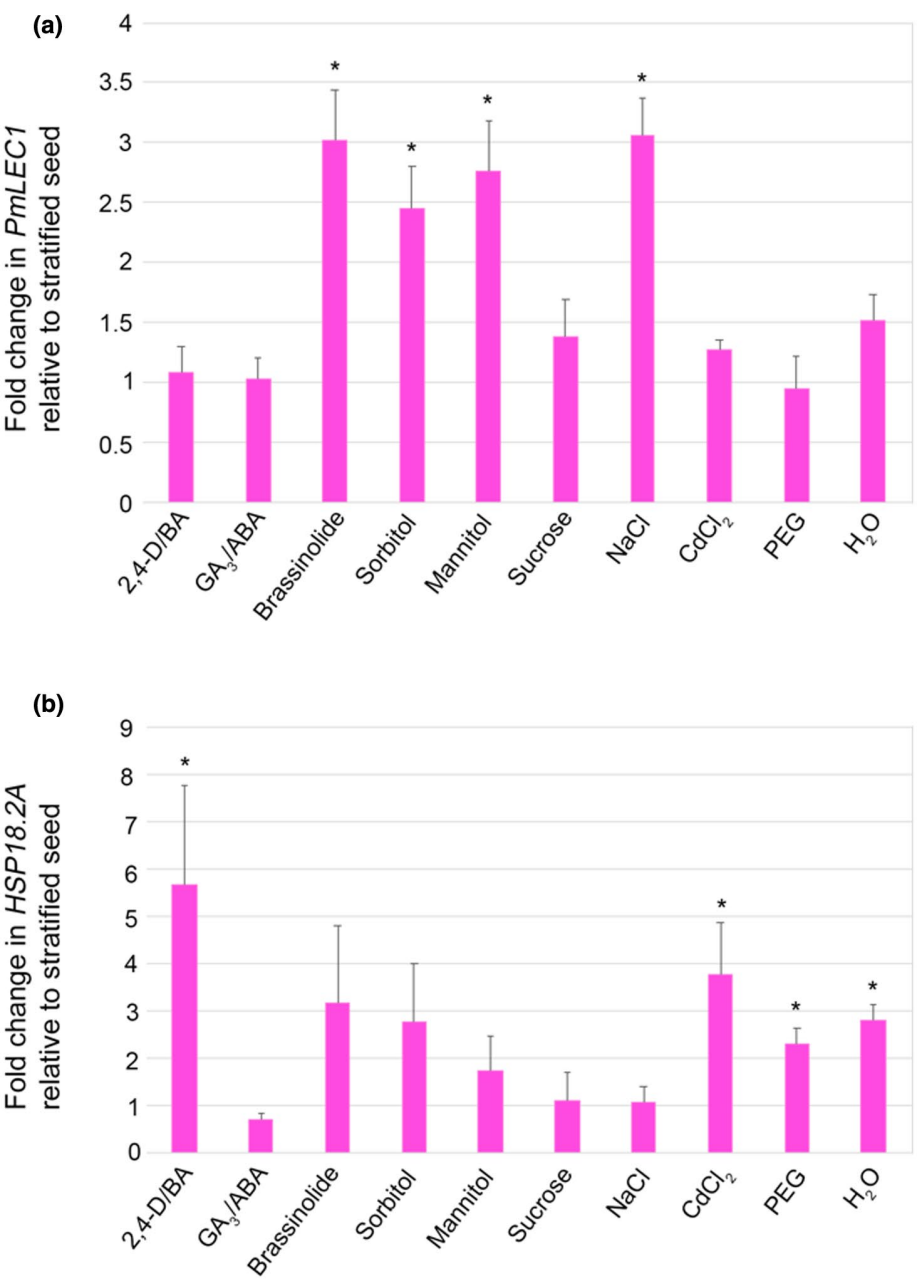
*PmLEC1* expression is upregulated in stratified seeds by treatment with 2,4‐epibrassinolide (10 μM), sorbitol (0.7 M), mannitol (0.7 M) or NaCl (0.3 M), while *HSP18.1* expression is not affected. A, Relative expression of the *PmLEC1* gene in seeds subjected to the indicated treatments for 24 hr. B, Relative expression of the *HSP18.1* gene in stratified seeds subjected to the indicated treatments for 24 hr. Total RNA was isolated from 3 seeds of each treatment group, treated with DNase I and used for reverse transcription (1 mg RNA each). The qRT‐PCR reactions were carried out in quadruplicate with Douglas‐fir ribosomal *L8* transcripts as internal controls. Relative gene expression was calculated according to the 2^‐ΔΔCt^ method (Dorak, 2006) by comparison to transcript levels in mature stratified seeds. Statistical analysis was performed on the pooled groups (*n* = 3) by the Mann‐Whitney *U* test. Results are shown as means ± SE (*n* = 3). Asterisks indicate transcript levels that are significantly different (*p* < .05) from stratified seeds

To assess whether *PmLEC1* expression may be upregulated in recalcitrant tissues, stratified seeds were exposed to stress and PGR treatments, followed by qRT‐PCR analysis. Each treatment group consisted of three individual seeds held at room temperature for 24 hr on filter paper soaked with specified compounds. The 2,4‐epibrassinolide (10 μM) treatment resulted in a threefold upregulation of *PmLEC1* (Figure 8a). The combinations of 50 μM 2,4‐D/20 μM BA and 7.2 μM GA_3_/38 μM ABA had no effect on *PmLEC1* expression (Figure 8a). Among the plasmolysing osmotic stresses, 0.7 M mannitol and 0.7 M sorbitol upregulated *PmLEC1* expression in stratified seeds by 2.5‐fold and 2.75‐fold, respectively, whereas 0.7 M sucrose had no significant effect (Figure 8). *PmLEC1* expression increased by threefold following 0.3 M NaCl treatment (Figure 8a). The other stress treatments, 0.6 mM CdCl_2_ (heavy metal), 23.75 mM polyethylene glycol (PEG) 8000 (nonplasmolysing osmotic) or immersion in water (hypotonic) did not affect *PmLEC1* expression (Figure 8a).

To verify whether these treatments were perceived as stresses, we measured the transcript levels of the heat shock protein, *PmHSP18*.*2A*, which shows specific responses to PGRs and stress after four days (Kaukinen et al., 1996). Treatment with 2,4‐D/BA upregulated *HSP18*.*2A* expression by 5.7‐fold (Figure 8b). *PmHSP18.2A* expression was also upregulated 3.8‐fold by CdCl_2_ treatment, 2.3‐fold by PEG, and 2.8‐fold by immersion in water (Figure 8b). The treatments that upregulated *PmLEC1* expression did not upregulate *PmHSP18.2A* (Figure 8b). In conclusion, PmLEC1 expression in stratified seeds responded in as few as 24 hr to both specific stressors and PGRs.

### 3.6 The regulatory landscape of *PmLEC1* contains a 5′ UTR intron, regulatory elements for physiological, biotic, and abiotic signals and seed‐specific transcription factors

**FIGURE 9:**
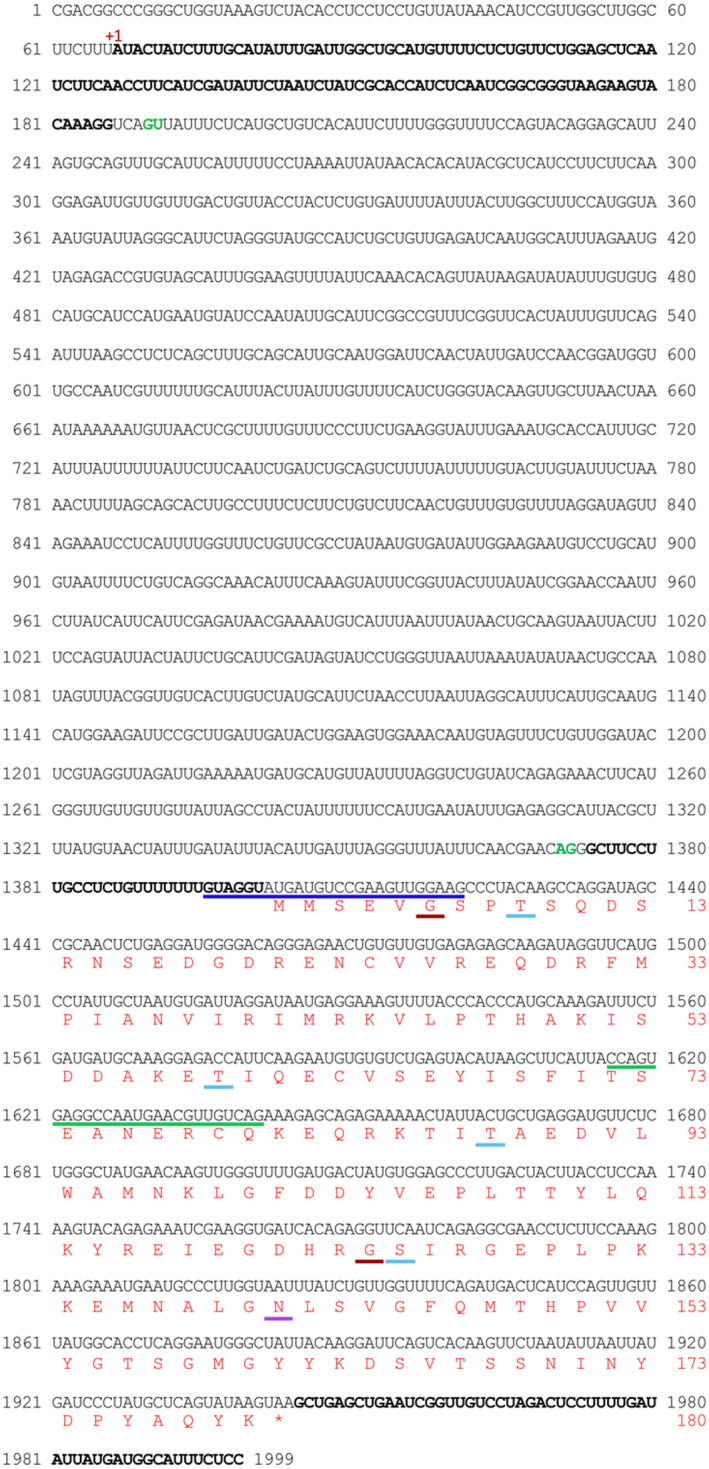
Complete *PmLEC1* nucleotide sequence, 5′ UTR intron and deduced protein sequence with putative sites for post‐translational modifications. The nucleotide sequence upstream of the coding region was obtained by genome walking. The green line corresponds to the complementary DNA sequence and represents GSP1, employed in the primary PCR reaction of genome walking, while the blue line corresponds to the complementary DNA sequence and represents GSP2 employed in the secondary PCR reaction. The 5′ UTR and 3′ UTR sequences are displayed in bold. The 5′ UTR is split into two sections by the 5′ UTR intron. The potential intron splice sites are in green. The deduced protein sequence is shown in red and numbered on the right. Putative sites of post‐translational modifications are underlined amino acid residues: dark‐green, N‐myristoylation; light‐blue, phosphorylation; violet, N‐glycosylation

To gain insight into the transcriptional regulation of *PmLEC1*, we isolated the genomic DNA sequence 1,400 bp upstream of the coding sequence. Alignment of the genomic sequence with the *PmLEC1* cDNA sequence revealed the presence of a 5′ UTR intron (Figure 9). An intron in the initial cDNA sequence was also indicated by an in‐frame stop codon within the 5′ UTR, signifying a splicing event (Soccio et al., 2002).

**FIGURE 10:**
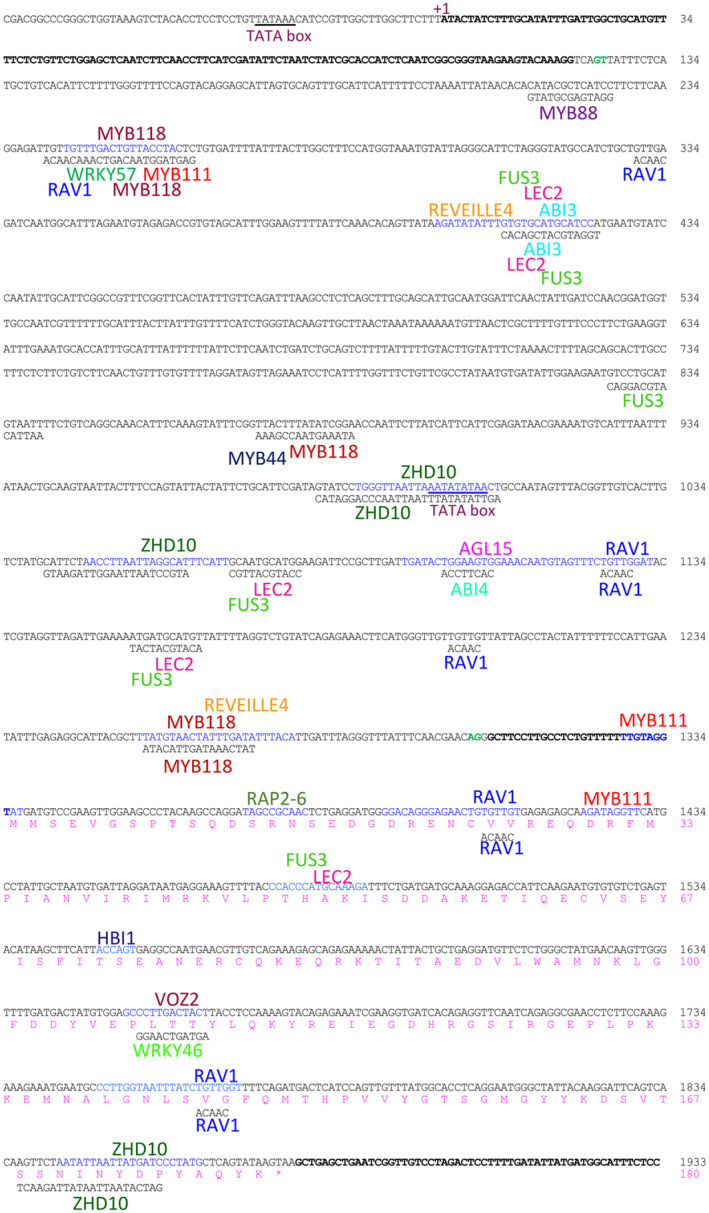
The Douglas‐fir *PmLEC1* gene map with regulatory elements and corresponding transcription factors. The nucleotide sequence of the sense strand is in black and numbered on the right in black. +1, transcriptional start site. The 5′ and 3′ UTRs are represented by bold letters. Intron splice site sequences are shown in green. Regulatory elements (sequences in blue, elements on coding strand; sequences in black, elements on template strand) and the corresponding names of binding factors identified via PlantPAN3.0. The putative protein sequence is shown in pink and numbered on the right in pink

Because 5′ UTR introns demonstrate promoter‐like activity (Kim et al., 2006), we queried the *PmLEC1* locus in PlantPAN3.0 (Chow et al., 2019), a resource for identifying regulatory elements in plant promoters. Putative TATA boxes were identified at −28 bp and +998 bp (Figure 10). Over 700 cis‐regulatory elements, with high similarity scores, were identified within the *PmLEC1* genomic sequence. The full list of regulatory elements, their positions and similarity scores, their associated TFs, source organisms, and the TF gene ontology biological processes are presented in Table S1.

Approximately 50 cis‐regulatory elements or TFs with well‐characterized functions in angiosperm embryogenesis were identified (Figure 10), suggesting potential culture medium manipulations that may facilitate conifer SE and identifying TFs that regulate *PmLEC1* transcription. The gene ontology biological processes represented by these regulatory elements and associated TFs include responses to salt stress, water stress, osmotic stress, metal ions, sugars, PGRs, defense responses to microbes and pests, chromatin remodeling, nucleosome assembly, and intron splicing (Table 1, Table S1). Photoperiodism, responses to specific wavelengths of light, circadian rhythm, and photomorphogenesis are also prominent. Several regulatory elements for TFs that have been well characterized in angiosperms and participate in SE, embryo development and seed maturation include AGL15, ABI3, LEC2, FUS3, WUS, VP1, and MYB118 (Table 1, Figure 10). Astonishingly, the cis‐elements for ABI3, LEC2, and FUS3 are collocated on the same nucleotide segment in four instances in the *PmLEC1* gene (Figure 10).

**TABLE 1:** Regulatory elements of the *PmLEC1* locus and associated transcription factors most relevant to embryo development and somatic embryogenesis (SE)

Cis*‐*element	Transcription factor	Source organism	Gene ontology—biological process
AGAMOUS	AGAMOUS	*A. thaliana*	Flower development.
MADS box; MIK3	AGL3	*A. thaliana*	Plant ovule, carpel, petal, sepal, stamen development; cell differentiation; maintenance of floral meristem identity; multicellular organism development.
MADS box; MIK3	AGL6	*A. thaliana*	Cell differentiation; floral organ development; vegetative to reproductive phase transition of meristem; multicellular organism and plant ovule development.
MADS box; MIK3	AGL15	*A. thaliana*	SE; cellular response to auxin stimulus; embryo development ending in seed dormancy; GA catabolic process; multicellular organism development; seed maturation, short‐day photoperiodism.
MADS box; MIK3	FLOWERING LOCUS C (FLC)	*A. thaliana*	Cell differentiation; multicellular organism development; regulation of circadian rhythm; response to temperature stimulus; vernalization response.
AP2; B3; RAV	RAV1	*A. thaliana*	Cellular response to hypoxia, brassinosteroids; ethylene‐activated signaling pathway; lateral root development; leaf development.
AP2; ERF	ABI4	*A. thaliana*	ABA‐activated, ethylene‐activated, and sugar‐mediated signaling pathways; defense response; response to glucose, osmotic stress, sucrose, trehalose, water deprivation; root meristem growth; seed development; starch catabolism.
B3; RY element	ABI3	*A. thaliana;* *B. napus*	ABA‐activated signaling pathway; embryo development ending in seed dormancy; response to ABA; response to auxin.
B3	LEC2	*A. thaliana*	SE; embryo development ending in seed dormancy; seed maturation; seed oilbody biogenesis; auxin biosynthetic process.
B3	FUS3	*A. thaliana*	SE; embryo development ending in seed dormancy; plant organ development; regulation of ABA and GA biosynthetic processes; regulation of ABA‐activated signaling pathway, cell population proliferation, embryonic development; response to auxin; vegetative to reproductive phase transition of meristem.
SPH CORE; RY REPEAT4	VP1	*Z. mays;* *T. aestivum; O. sativa*	Expression of the maturation program in seed development; potentiation of seed‐specific hormone response.
WOX	WUSCHEL (WUS)	*A. thaliana*	Anther development; axillary shoot meristem initiation; cell differentiation; stem cell population maintenance.
HD‐ZIP	MERISTEM L1	*A. thaliana*	Cotyledon development; plant epidermal cell differentiation; seed germination; cell specification and pattern formation during embryogenesis.
MYB‐related	CCA1	*A. thaliana*	Circadian rhythm; long‐day photoperiodism, flowering; response to ABA, auxin, cadmium ion, cold, ethylene, jasmonic acid, GA, organonitrogen compound, salicylic acid, salt stress.
Myb/SANT; MYB; G2‐like	KANADI 1 (KAN1)	*A. thaliana*	Abaxial cell fate specification; adaxial/abaxial axis specification; plant organ morphogenesis; plant ovule development; radial pattern formation; xylem and phloem pattern formation; polarity specification of adaxial/abaxial axis.
Cold Shock Domain	CSD protein 4 (CSP4)	*A. thaliana*	Embryo development ending in seed dormancy; response to cold. Regulates seed development, particularly at late stages of embryo development.
Myb/SANT; ARR‐B	Two‐component response regulator ARR1	*A. thaliana*	Maintenance of shoot apical meristem identity; regulation of root meristem growth, seed growth; response to cytokinin, water deprivation; root development; shoot system development.
NAC; NAM	NAC TF 29 (NAC029)	*A. thaliana*	Embryo development ending in seed dormancy; multidimensional cell growth; flower development; fruit ripening; leaf senescence.
YABBY	Axial regulator YABBY 1	*A. thaliana*	Abaxial cell fate specification; cell fate commitment; meristem structural organization; regulation of shoot apical meristem development; specification of plant organ position.
YABBY	CRABS CLAW	*A. thaliana*	Cell fate commitment; floral meristem determinacy; meristem determinacy; polarity specification of adaxial/abaxial axis.
AP2; ERF	Ethylene‐responsive TF RAP2‐6	*A. thaliana*	Cellular response to heat; chloroplast organization; defense response; ethylene‐activated signaling pathway; response to ABA, cold, jasmonic acid, osmotic stress, salicylic acid, salt stress, water deprivation, wounding.
ARID	ARID6	*A. thaliana*	Chromatin silencing; glucosinolate metabolic process.
bHLH	HBI1	*A. thaliana*	Brassinosteroid‐ and GA_3_‐mediated signaling pathways; regulation of growth.
Dof	CDF5	*A. thaliana*	Flower development; negative regulation of long‐day photoperiodism, flowering; negative regulation of short‐day; photoperiodism, flowering.
C3H	Splicing factor U2AF35B	*A. thaliana*	Photoperiodism; flowering; mRNA splicing via spliceosome.
Myb/SANT	MYB33	*A. thaliana*	Pollen sperm cell differentiation; ABA‐activated and GA3‐mediated signaling pathways; regulation of cell proliferation, growth, programmed cell death; protein storage vacuole organization; responses to cytokinin, ethylene, GA.
Myb/SANT; MYB	MYB44	*A. thaliana*	ABA‐activated signaling pathway; defense responses to bacteria and fungi; responses to ABA, auxin, cadmium ion, chitin, ethylene, GA, jasmonic acid, salicylic acid, salt stress, water deprivation.
Myb/SANT	MYB 65	*A. thaliana*	Cell differentiation; regulation of auxin mediated signaling pathway, protein storage vacuole organization, cell population proliferation, growth, programmed cell death; pollen sperm cell differentiation; response to ethylene, salicylic acid.
Myb/SANT; MYB	MYB 88	*A. thaliana*	Embryo sac development; guard cell and guard mother cell differentiation; megasporogenesis; responses to salt stress, ABA, water deprivation, gravity; water homeostasis; regulation of cell cycle G1/S phase transition.
Myb/SANT	MYB111	*A. thaliana*	Cell differentiation; flavanol biosynthetic process; responses to UV‐B and light.
Myb/SANT; MYB	MYB 118	*A. thaliana*	SE; regulation of embryo and endosperm development, seed maturation, fatty acid biosynthesis; vegetative to reproductive phase transition of meristem.
Myb/SANT; MYB‐related	REVEILLE 4 RVE4	*A. thaliana*	Circadian rhythm; response to ABA, auxin, cadmium ion, ethylene, GA, jasmonic acid, salicylic acid, salt stress.
Myb/SANT; MYB‐related	REVEILLE 8 (RVE8)	*A. thaliana*	Circadian regulation of gene expression; photoperiod; response to ABA, auxin, cadmium ion, ethylene, GA, jasmonic acid, salicylic acid, salt stress.
Myb/SANT; MYB‐related	LHY	*A. thaliana*	Circadian rhythm; long‐day photoperiodism, flowering; response to ABA, auxin, cadmium ion, cold, ethylene, GA, jasmonic acid, salicylic acid, salt stress.
Myb/SANT	KUA1	*A. thaliana*	Auxin homeostasis; postembryonic plant organ morphogenesis; response to ABA, absence of light, auxin, cadmium ion, ethylene, GA, salt stress, sucrose, jasmonic acid, salicylic acid; hypocotyl elongation in response to darkness.
Myb/SANT; MYB‐related	Telomere repeat‐binding factor 2	*A. thaliana*	Nucleosome assembly; response to ABA, auxin, cadmium ion, ethylene, GA, jasmonic acid, salicylic acid, salt stress.
WRKY	WRKY46	*A. thaliana*	Brassinosteroid‐mediated signaling pathway; cellular response to hypoxia; response to water deprivation, bacteria, chitin, and salicylic acid.
WRKY	WRKY57	*A. thaliana*	Response to osmotic stress, salt stress, water deprivation.
ZF‐HD	ZHD1	*A. thaliana*	Seed maturation; regulation of GA biosynthetic process.
ZF‐HD	ZHD10	*A. thaliana*	GA_3_‐mediated signaling pathway; response to blue light, GA.
VOZ	VOZ2	*A. thaliana*	Long‐day photoperiodism, flowering; positive regulation of long‐day photoperiodism; red, far‐red light phototransduction; response to salt stress.
Dehydrin	Protein COLD‐REGULATED 15A (COR15A)	*A. thaliana*	Circadian rhythm; cold acclimation; drought recovery; heat acclimation; leaf senescence; red or far‐red light signaling pathway; response to ABA, cold, freezing, light, osmotic stress, salt stress, water deprivation.
Others	RD29A (Desiccation‐responsive protein 29A)	*A. thaliana*	Circadian rhythm; hyperosmotic salinity response; leaf senescence; regulation of root development; response to abscisic acid, cold, mannitol, osmotic stress, reactive oxygen species, salt, salt stress, symbiotic bacterium, water deprivation, wounding; responses to abiotic stresses.
bHLH	PIF1	*A. thaliana*	Chlorophyll biosynthetic process; GA_3_‐mediated signaling pathway; regulation of photomorphogenesis, seed germination; red light signaling pathway.
bHLH	PIF3	*A. thaliana*	De‐etiolation; GA_3_‐mediated signaling pathway; red, far‐red light phototransduction; red or far‐red light signaling pathway and response.
PYRIMIDINE BOX; NF‐YC	NF‐YC9	*O. sativa;* *H. vulgare*	ABA‐activated signaling; GA_3_‐mediated signaling pathway; positive regulation of photomorphogenesis; regulation of seed germination.
Myb/SANT; MYB; NF‐YC	CDC5	*A. thaliana*	Cell cycle; defense response signaling pathway; defense responses to bacteria and fungi; DNA repair; innate immune response; RNA splicing.

### 3.7 Functional analysis of *PmLEC1* via ectopic expression in the *Arabidopsis lec1‐1* null mutant confirms somatic embryogenesis induction by *PmLEC1*

To elucidate the function of *PmLEC1*, we transformed the *A. thaliana lec1‐1* null mutant with a DNA construct, designed to express the *PmLEC1* gene under the control of a strong constitutive promoter. The *lec1‐1* null mutant is embryo‐lethal due to the deletion of the *AtLEC1* gene, which renders *lec1‐1* seeds intolerant to desiccation and nonviable (Lotan et al., 1998).

**FIGURE 11:**
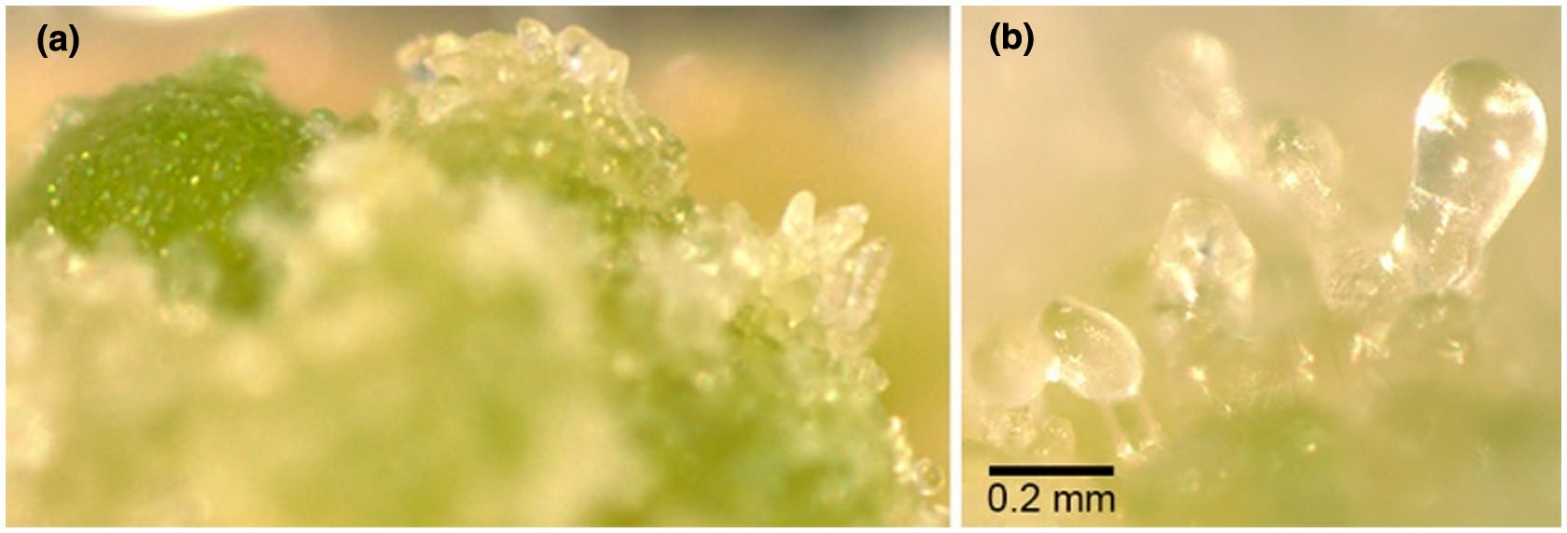
Ectopic expression of *PmLEC1* complements the *Arabidopsis lec1‐1* null mutation and leads to the formation of embryo‐like structures in T2 *lec1‐1^PmLEC1^* seedlings. (a) T2 *lec1‐1^PmLEC1^* seeds germinated on kanamycin‐containing MS medium, produced roots and cotyledons, and often masses of green and white embryo‐like structures grew recurrently on the cotyledons. (b) At higher magnification, these structures resembled late globular stage embryos

**FIGURE 12:**
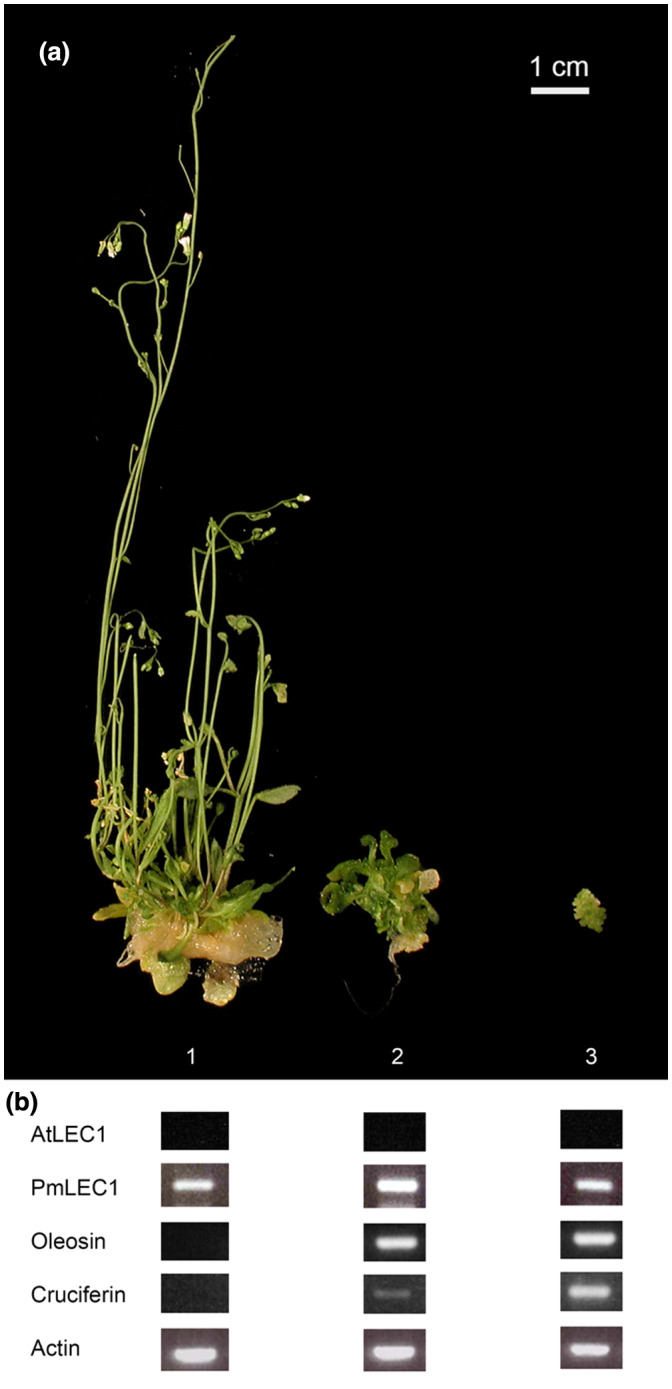
Ectopic expression of *PmLEC1* in the *A. thaliana lec1‐1* null mutant produces diverse T2 phenotypes and induces expression of embryo‐specific genes in mature tissues. (a) Plant morphology ranged from normal phenotype of *A. thaliana* (1) to seedlings with cotyledon‐like organs at positions normally occupied by leaves (2), and seedlings with masses of recurrent embryo‐like structures emerging from the cotyledons (3). (b) Gene expression analysis. Total RNA was isolated only from vegetative tissues (leaves and stems). RT‐PCR was performed with 0.1 μg DNase I‐treated RNA to evaluate gene expression of *AtLEC1*, *PmLEC1*, *oleosin* and *cruciferin*. *Actin* served as the control

*The PmLEC1* transgene complemented the *lec1‐1* mutation and produced viable, desiccated T1 seeds from transformed *lec1‐1* mutants. Transgenic T1 seeds that produced viable and fertile plants were self‐pollinated to produce T2 seeds, which were germinated on kanamycin‐containing medium. We performed molecular and morphometric analyses of this T2 generation, T2 *lec1‐1^PmLEC1^*. PCR analysis confirmed stable integration of the *PmLEC1* transgene into all selected *lec1‐1^PmLEC1^* lines. The T2 *lec1‐1^PmLEC1^* lines displayed various phenotypes, ranging from normal morphological characteristics of wild‐type *A. thaliana* to seedlings with recurrent, embryo‐like structures on vegetative tissues (Figures 11 and 12). These embryo‐like *lec1‐1^PmLEC1^* seedling phenotypes were similar to those observed by Lotan et al. (1998) in the *lec1‐1* mutants transformed with the *AtLEC1* gene. Thus, the ectopic expression of *PmLEC1* not only rescued the *lec1‐1* mutants but also affected vegetative development of T2 *lec1‐1^PmLEC1^* seedlings after embryogenesis. Most importantly, the *lec1‐1^PmLEC1^* seedlings exhibiting an embryo‐like phenotype accumulated transcripts of the embryo‐specific genes, *oleosin* and *cruciferin* (Figure 12). These results indicate that ectopic expression of the *PmLEC1* gene activated embryonic programs in vegetative tissues of the host plant and induced the morphogenesis stage of embryo development.

## 4 DISCUSSION

The *LEC1* homologue from Douglas‐fir, *PmLEC1*, was isolated and characterized to gain insight into conifer embryogenesis. Phylogenetic analysis revealed that PmLEC1 was most closely related to AtLEC1, a central regulator of embryogenesis in Arabidopsis. Similarly to *AtLEC1*, ectopic expression of *PmLEC1* in the Arabidopsis *lec1‐1* null mutant rescued the mutant, led to the appearance of embryo‐like structures and embryo‐like seedlings in the T2 generation, and induced embryonic programs in mature T2 tissues. Unique features of *PmLEC1* include a 5′ UTR intron, an alternating pattern of gene expression and protein levels that persist until germination stages. Our results identify *PmLEC1* as an embryo‐regulatory TF whose expression in mature tissues is upregulated by brassinosteroids, sorbitol, mannitol, and NaCl. The isolation and characterization of *PmLEC1* is a crucial first step in not only understanding the molecular basis of conifer embryogenesis but also overcoming recalcitrance to SE induction. Homologs of specific TFs critical to angiosperm embryogenesis deduced from the cis‐regulatory elements in the 5′ UTR intron and are likely to be involved in conifer embryogenesis. Their identification and characterization may unlock the enigmatic process of conifer embryogenesis that has eluded researchers for the last 50 years. The cis‐regulatory elements also provide pertinent insights into culture medium components and TFs that may induce SE or promote robust embryo production.

### 4.1 *PmLEC1* as a master regulator of embryogenesis in Douglas‐fir

Functional analysis of *PmLEC1* via ectopic expression in the Arabidopsis *lec1‐1* mutant confirmed that *PmLEC1* complemented the *lec1‐1* mutation and induced embryonic programs and the generation of embryo‐like structures from vegetative tissues (Figures 11 and 12), implying that *PmLEC1* directs embryo formation, embryo maturation and desiccation tolerance. The induction of embryonic programs in vegetative cells of a heterologous host suggests that *PmLEC1* may orchestrate SE in Douglas‐fir, and possibly other conifers. As a master regulator, PmLEC1 would affect genes responsible for morphogenesis, embryo maturation, and seed development. Indeed, during conifer SE, expression of numerous genes involved in metabolism, cellular processes, and information storage and processing exhibit an alternating up‐down‐up pattern (Stasolla et al., 2004; van Zyl et al., 2003) that closely resembles the alternating pattern of *PmLEC1* gene expression we observed (Figures 4 and 5). The relationship between *PmLEC1* activity and embryogenic transcript levels will provide insight into its regulatory capacity. The functional differences between PmLEC1 and AtLEC1 remain to be discovered, yet the use of *PmLEC1* is a promising strategy for SE induction.

### 4.2 *PmLEC1* expression as a molecular marker of embryogenic potential

The mechanisms underlying pattern formation during plant embryogenesis remain unknown, and the characterization of essential genes should remain a focus of research. Because many of these genes are active for the duration of embryogenesis, the knowledge of precise patterns of gene expression will help to elucidate “normal” embryogenesis and/or identify embryogenic tissues.

A precise and consistent pattern of *PmLEC1* gene expression was demonstrated in both ZE and SE by northern blot analysis and qRT‐PCR (Figures 4 and 5), with peaks in transcript levels corresponding to the beginning of the appearance of morphological stages (proembryo, early embryo, late embryo) of ZE. The large error bar on June 20 (Figure 5) may reflect asynchronous fertilization leading to embryos that are not uniformly at the same stage of development (Owens, 1995). The alternating pattern of induction and repression observed in both ZE and SE is reminiscent of a phenomenon described by Cairney and Pullman (2007), whereby embryogenic lines exhibit an “up‐down‐up” expression pattern of some genes while a pattern reversal occurs in nonembryogenic lines. Consequently, the pattern and level of gene expression may differentiate embryogenic from those of nonembryogenic lines. The alternating *PmLEC1* expression pattern we observed may be the embryogenic one, with the “up” representing the beginning of the developmental stages.

The presence of trace amounts of *PmLEC1* transcripts and protein in germinating seeds (Figures 6 and 7) ascertains its role in the transition from dormant seed to vegetative growth because low levels of *AtLEC1* transcripts were observed in germinating 5‐day‐old Arabidopsis seedlings (Huang et al., 2015). However, the expression of trace amounts of *LEC1* in germinating seeds and the youngest vegetative buds and pollen cones, may also be an indicator of their embryogenic competence and the window for inducing SE from these juvenile tissues.

### 4.3 5′ UTR introns enhance gene expression and protein accumulation

Our results are the first to describe a *LEC1* gene with a 5′ UTR intron. Some of the known *LEC1* genes contain introns within the coding sequence, but no 5′ UTR introns have been evident (Cagliari et al., 2014; Han et al., 2017). Only a few conifer genomes have been sequenced; most *LEC1* sequence data are derived from cDNAs and ESTs. 5′ UTR introns are associated with promoter‐like features, developmental regulation and increased gene expression *via* IME (Chung et al., 2006; Kim et al., 2006).

Normally, 5′ UTR introns are located 80–300 bp from the beginning of a 5′ UTR and 1–40 bp upstream of the start codon and are usually longer than introns within coding sequences (Chung et al., 2006). Large 5′ UTR introns function as spacers within the genome and provide AT‐rich stimulatory elements between coding and noncoding sequences of the genome (Chung et al., 2006). The *PmLEC1* 5′ UTR intron splice sites are located 123 bp from the beginning of the 5′ UTR and 30 bp upstream of the start codon. The *PmLEC1* intron is 1,176 bp in length, more than twice the size of the 543 bp coding sequence.

Multiple factors and signals appear to regulate the *PmLEC1* locus (Figure 9, Table 1). Large numbers of elements mediating responses to a variety of signals couple brief stimuli to long‐term adaptive responses (Finkbeiner, 2001). Therefore, the use of multiple signals for synergistic activation may be one strategy to induce SE at any time and in any tissue. Multiple regulatory sites on introns lead to synergistic activation rather than additive activation, and specific biological responses may be achieved combinatorially (Finkbeiner, 2001). Combinatorial responses result when two signaling pathways converge on two or more TFs that cooperate (Finkbeiner, 2001). In angiosperms, gain‐of‐function and loss‐of‐function mutants demonstrate that LEC2, *LEC1/FUS3*, *LEC1/ABI3*, and *FUS3/ABI3* interact synergistically during seed development and maturation (Kagaya et al., 2005; Lotan et al., 1998; Parcy et al., 1997; Vicient et al., 2000). We found that the *PmLEC1* intron contains binding sites for ABI3, FUS3, and LEC2; the multiple pathways that converge include biotic and abiotic stress responses, light perception, developmental cues and phytohormone signaling (Figure 9, Table 1).

### 4.4 PmLEC1 protein profile, electrophoretic mobility, and possible posttranslational modifications

The estimated sizes of PmLEC1, 59 kD and 36 kD, as observed via electrophoretic mobility, are greater than the 21 kD MW, initially predicted for the 180 amino acid residues, but they are likely correct assuming predicted posttranslational modifications of PmLEC1. Similarly, murine HAP3 (NF‐YB) proteins are known to migrate at higher‐than‐expected MWs. Both rat and mouse HAP3 proteins contain 207 amino acids and have expected MWs of 25 kD, but they migrate at 32 kD and 36 kD, respectively (Gilthorpe et al., 2002; Vuorio et al., 1990). The SDS‐PAGE mobility of PmLEC1 protein extracted from vegetative tissues of transgenic *WT^PmLEC1^* Arabidopsis plants is 34 kD and is comparable to that of HAP3 proteins purified from other species.

The change in PmLEC1 electrophoretic mobility from July 31 until 10 DAEG (Figure 8) coincides with the maturation and desiccation phases of embryogenesis, and seed dormancy. The maturation phase is characterized by an interruption in patterning, proliferation and differentiation, accumulation of storage proteins and lipids and a reduction in water content (Jo et al., 2019). In Arabidopsis, AtLEC1 inhibits precocious germination and is necessary for the acquisition of desiccation tolerance and accumulation of storage molecules (Jo et al., 2019). The embryo remains developmentally arrested until conditions become favorable for germination (Jo et al., 2019), which involves increased water uptake and metabolic activity. The change from 36 to 59 kD occurring at 12 DAEG could signify that favorable germination conditions were finally perceived.

Posttranslational modifications would allow PmLEC1 to function during all stages of embryogenesis. The capture of a sequential transition is likely due to seeds at different stages of late embryogenesis (each sample contained proteins from several seeds), and the two visible steps on July 17 and July 31 are suggestive of the two predicted N‐myristoylations. N‐myristoylation functions in protein–protein interactions, protein stability and adaptation to salt stress, cellular localization, targeting to membranes, and acting as a molecular switch (de Jonge et al., 2000; Wright et al., 2010).

### 4.5 Potential regulators of *PmLEC1* suggest molecular events coordinating conifer embryogenesis

Regulatory elements within the 5′ UTR intron are specific for TFs that have well‐characterized functions in angiosperm embryogenesis and imply that homologues of AGL15, LEC2, FUS3, VP1, ABI3, and/or WUS regulate *PmLEC1* expression. Because AGL15 is present prior to and during early embryogenesis, it may be the most significant factor responsible for *LEC1* induction. In Brassica, maize and Arabidopsis, AGL15 is the only MADS box protein that preferentially accumulates in developing embryos and associated maternal tissues, as well as in somatic embryos (Harding et al., 2003; Perry et al., 1996; Tang & Perry, 2003). Ectopic expression of AGL15 in angiosperms promotes and supports somatic embryo production (Harding et al., 2003). During middle and late angiosperm embryogenesis, transcripts of the seed‐specific transcriptional activators VP1 and ABI3 are the most abundant (Kurup et al., 2000; Suzuki et al., 2007).

The presence of regulatory elements for brassinosteroids, salt, and mannitol supports our findings on PmLEC1 induction by these compounds (Figure 8). For example, RAV1, HBI1, and WRKY46 mediate responses to brassinosteroids and these target sites are also present in *PmLEC1* (Table 1). Computational analysis of the PmLEC1 locus identified key TFs that are likely to have roles in conifer embryogenesis and should be characterized in future studies.

In summary, *PmLEC1* is the first early embryo regulatory gene characterized in Douglas‐fir. This gene is highly expressed during early embryogenesis and may be induced by salt, osmotic stress, and brassinosteroids in mature tissues. The induction of *PmLEC1* in mature tissues may lead to improved SE protocols and, most importantly, the induction of SE from vegetative conifer explants. The 5′ UTR intron sequence of *PmLEC1* identifies potential genes involved in conifer embryogenesis. The function of PmLEC1 may involve integrating multiple signals perceived through the intron and initiating major reprogramming to coordinate development and responses to the environment. Finally, since LEC1 is known to be part of a regulatory network (Han et al., 2017; Rupps et al., 2016), other TFs must be characterized before we may fully control embryogenesis in conifers.

## CONFLICT OF INTEREST

The authors are not aware of any conflicts of interest.

## AUTHOR CONTRIBUTIONS

MV and SM conceived the original project. MV designed and performed the experiments. DPY assisted with design and experimental procedures. All the authors contributed to data analysis. MV drafted the manuscript. MV and DPY revised and finalized the manuscript. MV agrees to serve as the author responsible for contact and ensures communication.

## Supporting information

Fig S1

Table S1‐S2

## References

[pld3333-bib-0001] Allen, G. S. , & Owens, J. N. (1972). The life history of Douglas fir. Environment Canada, Canadian Forestry Service.

[pld3333-bib-0002] Altschul, S. F. , Gish, W. , Miller, W. , Myers, E. W. , & Lipman, D. J. (1990). Basic local alignment search tool. Journal of Molecular Biology, 215, 403–410. 10.1016/S0022-2836(05)80360-2 2231712

[pld3333-bib-0003] Bradford, M. M. (1976). A rapid and sensitive method for the quantitation of microgram quantities of protein utilizing the principle of protein‐dye binding. Analytical Biochemistry, 7, 248–254. 10.1016/0003-2697(76)90527-3 942051

[pld3333-bib-0004] Cagliari, A. , Turchetto‐Zolet, A. C. , Korbes, A. P. , Maraschin Fdos, S. , Margis, R. , & Margis‐Pinheiro, M. (2014). New insights on the evolution of Leafy cotyledon1 (LEC1) type genes in vascular plants. Genomics, 103, 380–387. 10.1016/j.ygeno.2014.03.005 24704532

[pld3333-bib-0005] Cairney, J. , & Pullman, G. S. (2007). The cellular and molecular biology of conifer embryogenesis. New Phytologist, 176, 511–536. 10.1111/j.1469-8137.2007.02239.x 17953539

[pld3333-bib-0006] Chow, C. N. , Lee, T. Y. , Hung, Y. C. , Li, G. Z. , Tseng, K. C. , Liu, Y. H. , Kuo, P. L. , Zheng, H. Q. , & Chang, W. C. (2019). PlantPAN3.0: A new and updated resource for reconstructing transcriptional regulatory networks from ChIP‐seq experiments in plants. Nucleic Acids Research, 47, D1155–D1163. 30395277 10.1093/nar/gky1081 PMC6323957

[pld3333-bib-0007] Chung, B. Y. W. , Simons, C. , Firth, A. E. , Brown, C. M. , & Hellens, R. P. (2006). Effect of 5’UTR on gene expression in *Arabidopsis thaliana* . BMC Genomics, 7, 120. 16712733 10.1186/1471-2164-7-120 PMC1482700

[pld3333-bib-0008] Clough, S. J. , & Bent, A. F. (1998). Floral dip: A simplified method for Agrobacterium‐mediated transformation of Arabidopsis thaliana. The Plant Journal, 16, 735–743. 10.1046/j.1365-313x.1998.00343.x 10069079

[pld3333-bib-0009] Datla, R. S. S. , Bekkaoui, F. , Hamerlindl, J. K. , Pilate, G. , Dunstan, D. I. , & Crosby, W. L. (1993). Improved high‐level constitutive foreign gene expression in plants using an AMV RNA4 untranslated leader sequence. Plant Science, 94, 139–149. 10.1016/0168-9452(93)90015-R

[pld3333-bib-0010] de Jonge, H. R. , Hogema, B. , & Tilly, B. C. (2000). Protein N‐myristoylation: Critical role in apoptosis and salt tolerance. Science Signaling, 63, pe1. 10.1126/stke.2000.63.pe1 11752628

[pld3333-bib-0011] De Verno, L. L. , Byrne, J. R. , Pitel, J. A. , & Cheliak, W. M. (1989). Constructing conifer genomic libraries: A basic guide. Information report, PI‐X‐88. Petawawa National Forestry Institute, Canadian Forest Service.

[pld3333-bib-0012] DeCastro, E. , Sigrist, C. J. A. , Gattiker, A. , Bulliard, V. , Langendijk‐Genevaux, P. S. , Gasteiger, E. , Bairoch, A. , & Hulo, N. (2006). ScanProsite: Detection of PROSITE signature matches and ProRule‐associated functional and structural residues in proteins. Nucleic Acids Research, 34, W362–W365. 10.1093/nar/gkl124 16845026 PMC1538847

[pld3333-bib-0055] Dorak, M. (2006). Real‐time PCR (1st ed.). London: Taylor & Francis.

[pld3333-bib-0013] Edgar, R. C. (2004). MUSCLE: A multiple sequence alignment method with reduced time and space complexity. BMC Bioinformatics, 5, 113. 15318951 10.1186/1471-2105-5-113 PMC517706

[pld3333-bib-0015] Finkbeiner, S. (2001). New roles for introns: Sites of combinatorial regulation of Ca^2+^‐ and cyclic AMP‐dependent gene transcription. Science, 94, PE1. 10.1126/stke.2001.94.pe1 11752669

[pld3333-bib-0016] Gasteiger, E. , Hoogland, C. , Gattiker, A. , Duvaud, S. , Wilkins, M. R. , Appel, R. D. , & Bairoch, A. (2005). In J. M. Walker (Ed.), The proteomics protocols handbook (pp. 571–607). Humana Press.

[pld3333-bib-0017] Gautier, F. , Label, P. , Eliášová, K. , Leplé, J. C. , Motyka, V. , Boizot, N. , Vondráková, Z. , Malbeck, J. , Trávníčková, A. , Le Metté, C. , Lesage‐Descauses, M. C. , Lomenech, A. M. , Trontin, J. F. , Costa, G. , Lelu‐Walter, M. A. , & Teyssier, C. (2019). Cytological, biochemical and molecular events of the embryogenic state in Douglas‐fir (Pseudotsuga menziesii [Mirb.]). Frontiers in Plant Science, 10, 118. 10.3389/fpls.2019.00118 30873184 PMC6403139

[pld3333-bib-0018] Gilthorpe, J. , Vandromme, M. , Brend, T. , Gutman, A. , Summerbell, D. , Totty, N. , & Rigby, P. W. J. (2002). Spatially specific expression of Hoxb4 is dependent on the ubiquitous transcription factor NFY. Development, 129, 3887–3899. 12135926 10.1242/dev.129.16.3887

[pld3333-bib-0019] Gupta, P. K. , & Timmis, R. (2005). Mass propagation of conifer trees in liquid cultures‐progress towards commercialization. Plant Cell, Tissue and Organ Culture, 81, 339–346. 10.1007/s11240-004-6654-1

[pld3333-bib-0020] Gupta, P. K. , Timmis, R. , Pullman, G. , Yancey, M. , Kreitinger, M. , Carlson, W. , & Carpenter, C. (1991). Development of an embryogenic system for automated propagation of forest trees. In I. K. Vasil (Ed.), Cell culture and somatic cell genetics of plants (Vol. 8, pp. 75–92). Academic press.

[pld3333-bib-0021] Han, J. D. , Li, X. , Jiang, C. K. , Wong, G. K. , Rothfels, C. J. , & Rao, G. Y. (2017). Evolutionary analysis of the *LAFL* genes involved in the land plant seed maturation program. Frontiers in Plant Science, 4, 439. 10.3389/fpls.2017.00439 PMC5379062 28421087

[pld3333-bib-0022] Harding, E. W. , Tang, W. , Nichols, K. W. , Fernandez, D. E. , & Perry, S. E. (2003). Expression and maintenance of embryogenic potential is enhanced through constitutive expression of AGAMOUS‐Like 15. Plant Physiology, 133, 653–663. 14512519 10.1104/pp.103.023499 PMC219041

[pld3333-bib-0023] Huang, M. , Hu, Y. , Liu, X. , Li, Y. , & Hou, X. (2015). Arabidopsis LEAFY COTYLEDON1 Mediates Postembryonic Development via Interacting with PHYTOCHROME‐INTERACTING FACTOR4. The Plant Cell, 27, 3099–3111. 10.1105/tpc.15.00750 26566918 PMC4682307

[pld3333-bib-0024] Jefferson, R. A. , Kavanagh, T. A. , & Bevan, M. W. (1987). GUS fusions: Beta‐glucuronidase as a sensitive and versatile gene fusion marker in higher plants. EMBO Journal, 6, 3901–3907. 10.1002/j.1460-2075.1987.tb02730.x PMC553867 3327686

[pld3333-bib-0025] Jo, L. , Pelletier, J. M. , & Harada, J. J. (2019). Central role of the LEAFY COTYLEDON1 transcription factor in seed development. Journal of Integrative Plant Biology, 61, 564–580. 10.1111/jipb.12806 30916433

[pld3333-bib-0026] Kagaya, Y. , Toyoshima, R. , Okuda, R. , Usui, H. , Yamamoto, A. , & Hattori, T. (2005). LEAFY COTYLEDON1 controls seed storage protein genes through its regulation of FUSCA3 and ABSCISIC ACID INSENSITIVE 3. Plant and Cell Physiology, 46, 399–406. 10.1093/pcp/pci048 15695450

[pld3333-bib-0027] Kaukinen, K. H. , Tranbarger, T. J. , & Misra, S. (1996). Post‐germination‐induced and hormonally dependent expression of low‐molecular‐weight heat shock protein genes in Douglas fir. Plant Molecular Biology, 30, 1115–1128. 10.1007/BF00019546 8704123

[pld3333-bib-0028] Kim, M. J. , Kim, H. , Shin, J. S. , Chung, C. H. , Ohlrogge, J. B. , & Suh, M. C. (2006). Seed‐specific expression of sesame microsomal oleic acid desaturase is controlled by combinatorial properties between negative cis‐regulatory elements in the SeFAD2 promoter and enhancers in the 5'‐UTR intron. Molecular Genetics and Genomics, 276, 351–368. 10.1007/s00438-006-0148-2 16862401

[pld3333-bib-0029] Kumar, S. , Stecher, G. , Li, M. , Knyaz, C. , & Tamura, K. (2018). MEGA X: Molecular Evolutionary Genetics Analysis across Computing Platforms. Molecular Biology and Evolution, 35, 1547–1549. 10.1093/molbev/msy096 29722887 PMC5967553

[pld3333-bib-0030] Kurup, S. , Jones, H. D. , & Holdsworth, M. J. (2000). Interactions of the developmental regulator ABI3 with proteins identified from developing *Arabidopsis* seed. The Plant Journal, 21, 143–155. 10743655 10.1046/j.1365-313x.2000.00663.x

[pld3333-bib-0031] Kwong, R. W. , Bui, A. Q. , Lee, H. , Kwong, L. W. , Fischer, R. L. , Goldberg, R. B. , & Harada, J. J. (2003). LEAFY COTYLEDON1‐LIKE defines a class of regulators essential for embryo development. The Plant Cell, 15, 5–18. 10.1105/tpc.006973 12509518 PMC143447

[pld3333-bib-0032] Laxa, M. (2017). Intron‐mediated enhancement: A tool for heterologous gene expression in plants? Frontiers in Plant Science, 7, 1977. 10.3389/fpls.2016.01977 28111580 PMC5216049

[pld3333-bib-0033] Lee, H. , Fischer, R. L. , Goldberg, R. B. , & Harada, J. J. (2003). Arabidopsis LEAFY COTYLEDON1 represents a functionally specialized subunit of the CCAAT binding transcription factor. Proceedings of the National Academy of Sciences of the United States of America, 100, 2152–2156. 10.1073/pnas.0437909100 12578989 PMC149974

[pld3333-bib-0034] Li, X. Y. , Mantovani, R. , Hooft van Huijsduijnen, R. , Andre, I. , Benoist, C. , & Mathis, D. (1992). Evolutionary variation of the CCAAT‐binding transcription factor NF‐Y. Nucleic Acids Research, 20, 1087–1091. 10.1093/nar/20.5.1087 1549471 PMC312095

[pld3333-bib-0035] Lotan, T. , Ohto, M. , Yee, K. M. , West, M. A. L. , Lo, R. , Kwong, R. W. , Yamagishi, K. , Fischer, R. L. , Goldberg, R. B. , & Harada, J. J. (1998). *Arabidopsis* LEAFY COTYLEDON1 is sufficient to induce embryo development in vegetative cells. Cell, 93, 1195–1205. 10.1016/S0092-8674(00)81463-4 9657152

[pld3333-bib-0036] Murashige, T. , & Skoog, F. (1962). A revised medium for rapid growth and bioassays with tobacco tissue cultures. Physiologia Plantarum, 15, 473–497. 10.1111/j.1399-3054.1962.tb08052.x

[pld3333-bib-0037] Owens, J. N. (1995). Constraints to seed production: Temperate and tropical forest trees. Tree Physiology, 15, 477–484. 10.1093/treephys/15.7-8.477 14965931

[pld3333-bib-0038] Parcy, F. , Valon, C. , Kohara, A. , Miséra, S. , & Giraudat, J. (1997). The ABSCISIC ACID‐INSENSITIVE3, FUSCA3, and LEAFY COTYLEDON1 loci act in concert to control multiple aspects of *Arabidopsis* seed development. The Plant Cell, 9, 1265–1277. 9286105 10.1105/tpc.9.8.1265 PMC156996

[pld3333-bib-0039] Perry, S. E. , Nichols, K. W. , & Fernandez, D. E. (1996). The MADS domain protein AGL15 localizes to the nucleus during early stages of seed development. The Plant Cell, 8, 1977–1989. 8953767 10.1105/tpc.8.11.1977 PMC161328

[pld3333-bib-0040] Pullman, G. S. , Mein, J. , Johnson, S. , & Zhang, Y. (2005). Gibberellin inhibitors improve embryogenic tissue initiation in conifers. Plant Cell Reports, 23, 596–605. 10.1007/s00299-004-0880-1 15688237

[pld3333-bib-0041] Pullman, G. S. , Zeng, X. , Copeland‐Kamp, B. , Crockett, J. , Lucrezi, J. , May, S. W. , & Bucalo, K. (2015). Conifer somatic embryogenesis: Improvements by supplementation of medium with oxidation‐reduction agents. Tree Physiology, 35, 209–224. 10.1093/treephys/tpu117 25716878

[pld3333-bib-0042] Pullman, G. S. , Zhang, Y. , & Phan, B. H. (2003). Brassinolide improves embryogenic tissue initiation in conifers and rice. Plant Cell Reports, 22, 96–104. 10.1007/s00299-003-0674-x 12879262

[pld3333-bib-0043] Rogozin, I. B. , Kochetov, A. V. , Kondrashov, F. A. , Koonin, E. V. , & Milanesi, L. (2001). Presence of ATG triplets in 5’ untranslated regions of eukaryotic cDNAs correlates with a ‘weak’ context of the start codon. Bioinformatics, 17, 890–900. 10.1093/bioinformatics/17.10.890 11673233

[pld3333-bib-0044] Rupps, A. , Raschke, J. , Rümmler, M. , Linke, B. , & Zoglauer, K. (2016). Identification of putative homologs of Larix decidua to BABYBOOM (BBM), LEAFY COTYLEDON1 (LEC1), WUSCHEL‐related HOMEOBOX2 (WOX2) and SOMATIC EMBRYOGENESIS RECEPTOR‐like KINASE (SERK) during somatic embryogenesis. Planta, 243, 473–488. 10.1007/s00425-015-2409-y 26476718

[pld3333-bib-0045] Soccio, R. E. , Adams, R. M. , Romanowski, M. J. , Sehayek, E. , Burley, S. K. , & Breslow, J. L. (2002). The cholesterol‐regulated StarD4 gene encodes a StAR‐related lipid transfer protein with two closely related homologues, StarD5 and StarD6. PNAS, 99, 6943–6948. 10.1073/pnas.052143799 12011452 PMC124508

[pld3333-bib-0046] Spiecker, H. (2019) Executive summary. In H. Spiecker , M. Lindner , & J. Schuler (Eds.), Douglas‐fir – An option for Europe. EFI What science can tell us 9. European Forest Institute.

[pld3333-bib-0047] Stasolla, C. , Bozhkov, P. V. , Chu, T. M. , Van Zyl, L. , Egertsdotter, U. , Suarez, M. F. , Craig, D. , Wolfinger, R. D. , Von Arnold, S. , & Sederoff, R. R. (2004). Variation in transcript abundance during somatic embryogenesis in gymnosperms. Tree Physiology, 24, 1073–1085. 10.1093/treephys/24.10.1073 15294754

[pld3333-bib-0048] Suzuki, M. , Wang, H.‐H.‐Y. , & McCarty, D. R. (2007). Repression of the LEAFY COTYLEDON1/B3 regulatory network in plant embryo development by VP1/ABSCISIC ACID INSENSITIVE 3‐LIKE B3 genes. Plant Physiology, 143, 902–911. 17158584 10.1104/pp.106.092320 PMC1803726

[pld3333-bib-0049] Tang, W. , & Perry, S. E. (2003). Binding site selection for the plant MADS domain protein AGL15. Journal of Biological Chemistry, 278, 28154–28159. 10.1074/jbc.M212976200 12743119

[pld3333-bib-0050] van Zyl, L. , Bozhkov, P. V. , Clapham, D. H. , Sederoff, R. R. , & von Arnold, S. (2003). Up, down and up again is a signature global gene expression pattern at the beginning of gymnosperm embryogenesis. Gene Expression Patterns, 3, 83–91. 10.1016/S1567-133X(02)00068-6 12609608

[pld3333-bib-0051] Verwoerd, T. C. , Dekker, B. M. M. , & Hoekema, A. (1987). A small scale procedure for the rapid isolation of plant RNAs. Nucleic Acids Research, 17, 2362. 10.1093/nar/17.6.2362 PMC317610 2468132

[pld3333-bib-0052] Vicient, C. M. , Bies‐Estheve, N. , & Delseny, M. (2000). Changes in gene expression in the leafy cotyledon1 (lec1) and fusca3 (fus3) mutants of *Arabidopsis thaliana* L. Journal of Experimental Botany, 51, 995–1003. 10.1093/jexbot/51.347.995 10948227

[pld3333-bib-0053] Vuorio, T. , Maity, S. N. , & de Crombrugghe, B. (1990). Purification and molecular cloning of the “A” chain of a rat heteromeric CCAAT‐binding protein. Journal of Biological Chemistry, 265, 22480–22486. 2266139

[pld3333-bib-0054] Wright, M. H. , Heal, W. P. , Mann, D. J. , & Tate, E. W. (2010). Protein myristoylation in health and disease. Journal of Chemical Biology, 3, 19–35. 10.1007/s12154-009-0032-8 19898886 PMC2816741

