# Supplementary material for: Douglas‐fir LEAFY COTYLEDON1 (PmLEC1) is an active transcription factor during zygotic and somatic embryogenesis
Source: Plant Direct. 2021 Jul 29;5(7):e00333. doi: 10.1002/pld3.333 (PMC8320655; doi:10.1002/pld3.333)
Supplement: Supplementary file 1 — Fig S1 [file PLD3-5-e00333-s001.docx]

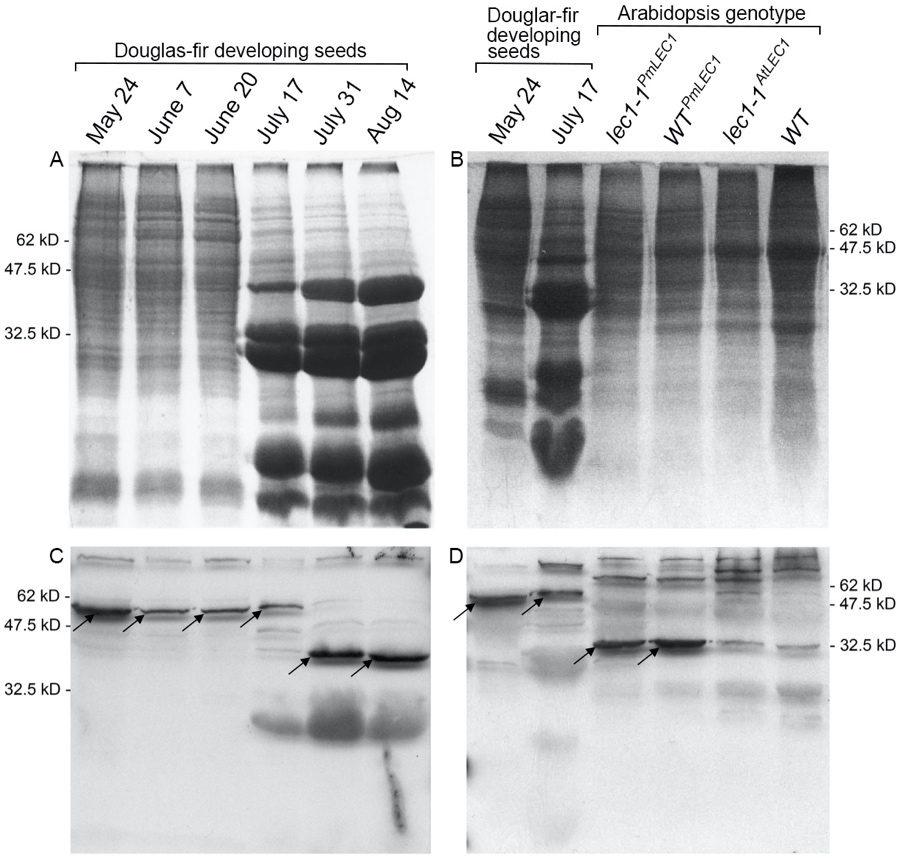


**Supplemental Figure S1**. Evaluating specificity and cross-reactivity of the polyclonal anti-PmLEC1 antiserum. Transgenic *lec1-1^AtLEC1^* and *WT^PmLEC1^* plants were generated via the floral dip method and grown to T2 generation. Total protein extracts (20 μg each) from leaf and stem tissues of *A. thaliana* transgenic and wild type plants, as well as from developing seeds harvested from Douglas-fir trees at the indicated dates, were resolved by SDS-PAGE, transferred to PVDF membranes and detected with anti-PmLEC1 antiserum. The arrows indicate PmLEC1-specific bands observed in lanes of *WT^PmLEC1^* plants at ~34 kD, and in Douglas-fir developing seeds at ~36 kD and ~59 kD. A, B, SDS-PAGE gels stained with Coomassie brilliant blue confirm equal loading and illustrate the changing protein profile of developing Douglas-fir seeds. C, D, Western blots.

*lec1-1^PmLEC1^*, Arabidopsis *lec1-1* mutant transformed with *PmLEC1*.

*WT^PmLEC1^*, Arabidopsis wild type plant transformed with *PmLEC1*.

*lec1-1^AtLEC1^*, Arabidopsis *lec1-1* mutant transformed with *AtLEC1*.

WT, Arabidopsis wild type plant.
