# Supplementary material for: Douglas‐fir LEAFY COTYLEDON1 (PmLEC1) is an active transcription factor during zygotic and somatic embryogenesis
Source: Plant Direct. 2021 Jul 29;5(7):e00333. doi: 10.1002/pld3.333 (PMC8320655; doi:10.1002/pld3.333)
Supplement: Supplementary file 2 — Table S1‐S2 [file PLD3-5-e00333-s002.docx]

Supplemental Tables

**Supplemental Table S1:** Regulatory elements, their position and similarity scores, associated transcription factors and related biological processes most relevant to embryo development and somatic embryogenesis. Position: positive numbers indicate location on the sense DNA strand and negative numbers indicate location on the antisense DNA strand. Similarity scores, 0 to 1, are shown in bold.

| **Cis element** | **Position**  **(similarity score)** | **Transcription Factor** | **Source organism** | **Gene ontology - biological process** |  |
| --- | --- | --- | --- | --- | --- |
| AGAMOUS CONSENSUS | + 222 **(0.94)**  + 1613 **(0.82**)  + 1018 **(0.72**) | AGAMOUS | *Arabidopsis thaliana* | Binding consensus sequence for the product of the Arabidopsis floral homeotic gene AGAMOUS (AG); AG protein contains a region similar to the DNA binding domain of SRF and MCM1; consensus sequence contains a CArG box; AG protein is a putative transcription factor for floral genes; MADS domain and I region of AGAMOUS are sufficient and necessary for DNA binding. |  |
| CARG3-AT-AP3 | + 1018 **(0.72**) | APETALA1, APETALA3, PISTILLATA, AGAMOUS | *Arabidopsis thaliana* | CArG3 found in the Arabidopsis APETALA3 gene promoter; Binding site of AP3/PI heterodimer; Binding site for a negatively acting factors; Binding sequence of Arabidopsis MADS domain homeotic proteins APETALA1, APETALA3, PISTILLATA, and AGAMOUS; AP1, AG, and AP3-PI complexes induce similar conformational changes on a CArG-box sequence. |  |
| MADS box; MIK3 | - 830 **(0.85)**  + 939 **(0.85)** | AGL3 | *Arabidopsis thaliana* | Carpel, petal, sepal, stamen, and plant ovule development; cell differentiation; maintenance of floral meristem identity; multicellular organism development; positive regulation of transcription by RNA polymerase II. |  |
| MADS box; MIK3 | + 1163 (**0.81**) | AGL6 | *Arabidopsis thaliana* | Cell differentiation; floral organ development; multicellular organism development; plant ovule development; positive regulation of flower development; positive regulation of transcription by RNA polymerase II; vegetative to reproductive phase transition of meristem. |  |
| MADS box; MIK3 | + 1792 **(0.85)** | AGL13 | *Arabidopsis thaliana* | Multicellular organism development; plant ovule and pollen development; positive regulation of transcription by RNA polymerase II; regulation of gene expression. |  |
| MADS box; MIK3 | - 1161 (**0.85**) | AGL15 | *Arabidopsis thaliana* | SE. Cellular response to auxin stimulus; embryo development ending in seed dormancy; floral organ abscission; fruit abscission; fruit dehiscence; GA catabolic process; multicellular organism development; negative regulation of floral organ abscission, flower development, gene expression, seed maturation, short-day photoperiodism, transcription; positive regulation of transcription and transcription by RNA polymerase. |  |
| MADS box; MIKC | - 1164 **(0.88**)  - 1159 **(0.82)** | AGL16 | *Arabidopsis thaliana* | Flower development; positive regulation of transcription by RNA polymerase II; stomatal lineage progression. |  |
| MADS box; MIK3 | - 1561 **(1)** | AGL42 | *Arabidopsis thaliana* | Abscission; cell differentiation; floral organ senescence; leaf senescence; multicellular organism development; positive regulation of transcription by RNA polymerase II; regulation of flower development |  |
| MADS box; MIKC | + 1793 **(0.84)** | AGL63 | *Arabidopsis thaliana* | Fruit development; fruit morphogenesis; multicellular organism development; plant ovule development; integument development; negative regulation of cell growth; positive regulation of transcription by RNA polymerase II; regulation of timing of transition from vegetative to reproductive phase. |  |
| MADS box; MIK3 | + 1017 (**0.86**)  - 1163 **(0.89**)  - 1165 (**0.84**)  - 1518 (**0.86**)  + 1790 (**0.87**) | FLOWERING LOCUS C (FLC) | *Arabidopsis thaliana* | Cell differentiation; flower development; multicellular organism development; negative regulation of flower development; positive regulation of transcription by RNA polymerase II; regulation of circadian rhythm; response to temperature stimulus; vernalization response. |  |
| AP2; B3; RAV | - 307 (**1**)  - 395 (**1**)  + 1176 (**1**)  + 1189 (**1**)  + 1187 (**0.97**)  - 1187 (**0.98**)  - 1265 (**1**)  + 1458 (**0.88**)  - 1473 (**1**)  + 1815 (**0.88**)  +1826 (**0.97**)  -1826 (**0.97**)  + 1828 **(1)**  - 1830 (**1**) | RAV1 | *Arabidopsis thaliana* | Cellular response to hypoxia; ethylene-activated signaling pathway; lateral root development; leaf development; negative regulation of flower development; negative regulation of transcription, DNA-templated; response to brassinosteroid  Binds specifically to bipartite recognition sequences composed of two unrelated motifs, 5'-CAACA-3' and 5'-CACCTG-3'. May function as negative regulator of plant growth and development. |  |
| AP2; B3 | + 1629 **(0.95)**  - 1629 **(0.95)** | AP2/ERF | *Arabidopsis thaliana* | Ethylene-activated signaling pathway. |  |
| AP2 | + 1201 **(1)**  - 1112 **(1)** | TOE2;  TOE1 | *Arabidopsis thaliana* | Ethylene-activated signaling pathway. |  |
| AP2; ERF | + / -  88 sites | RAP2-2 | *Arabidopsis thaliana* | Ethylene-activated signaling pathway; regulation of gene expression; response to hypoxia. |  |
| AP2; ERF | - 1167 **(1)** | ABI4 | *Arabidopsis thaliana* | ABA-activated and ethylene-activated signaling pathways; defense response; lateral root development; mitochondria-nucleus signaling pathway; positive regulation of transcription; regulation of L-ascorbic acid biosynthetic process; regulation of protein localization; regulation of stomatal movement; regulation of triglyceride catabolic process; response to glucose, osmotic stress, sucrose, trehalose, water deprivation; root meristem growth; seed development; starch catabolic process; sugar mediated signaling pathway. |  |
| B3 | - 478 (**0.97**)  + 479 **(0.97**) | ABI3 | *Arabidopsis thaliana* | ABA-activated signaling pathway; embryo development ending in seed dormancy; mitochondria-nucleus signaling pathway; plastid organization; positive regulation of transcription; response to ABA, auxin. |  |
| RY-REPEAT-BN-NAPA | - 93 (**1**)  - 479 (**1**)  + 481 (**1**)  - 896 (**1**)  - 1139 (**1**)  - 1224 (**1**)  + 1546 (**1**) | ABI3 | *Brassica napus* | RY repeat found in RY/G box (the complex containing the two RY repeats and the G-box) of napA gene in Brassica napus Required for seed specific expression; dist B ABRE mediated transactivation by ABI3 and ABI3-dependent response to ABA; a tetramer of the composite RY/G complex mediated only ABA-independent transactivation by ABI3; B2 domain of ABI3 is necessary for ABA-independent and ABA-dependent activation through the dist B ABRE. |  |
| B3 | + 480 (**1**)  - 476 (**0.99**)  - 478 (**0.95**)  - 1138 (**0.87**)  - 1223 (**0.87**)  + 1544 (**0.99**) | LEC2 | *Arabidopsis thaliana* | SE. Embryo development ending in seed dormancy;  positive regulation of auxin biosynthetic process; positive regulation of transcription; seed maturation; seed oilbody biogenesis. |  |
| B3 | - 475 (**0.96**)  + 476 (**0.97**)  - 478 **(0.97)**  + 479 **(0.97)**  - 892 (**0.95**)  - 1135 (**0.95**)  - 1220 **(0.96)**  + 1541 (**0.95**) | FUS3 | *Arabidopsis thaliana* | SE. Embryo development ending in seed dormancy; negative regulation of GA biosynthetic process; plant organ development; positive regulation of ABA-activated signaling pathway and ABA biosynthetic process; positive regulation of cell population proliferation; vegetative to reproductive phase transition of meristem; regulation of embryonic development; response to auxin. Transcription regulator involved in gene regulation during late embryogenesis. Its expression to the epidermis is sufficient to control foliar organ identity by regulating positively the synthesis ABA and negatively GA production. Negatively regulates TTG1 in the embryo. Positively regulates the abundance of the ABI3 protein in the seed. Cooperates with KIN10 to regulate developmental phase transitions and lateral organ development and both act as positive regulators of ABA signaling during germination. |  |
| AT-Hook | - 991 **(0.96)**  + 1059 **(0.97)**  + 719 **(0.97)** | AGF1; AHL25 | *Arabidopsis thaliana* | Negative regulation of GA_3_-mediated signaling pathway; vegetative to reproductive phase transition of meristem. |  |
| WOX | + 965 (**0.92**)  - 965 (**0.90**) | WUSCHEL  (WUS) | *Arabidopsis thaliana* | Anther and stomium development; axillary shoot meristem initiation; cell differentiation; regulation of transcription; stem cell population maintenance. |  |
| WOX | + 992 **(0.91)**  + 1056 **(0.95)**  - 1057 **(0.99)**  + 1115 **(0.93)**  + 1911 **(0.93)** | WUSCHEL-related homeobox 11 (WOX11) | *Arabidopsis thaliana* | Multicellular organism development. |  |
| HomeodomainbZIP;  HD-ZIP;  WOX | - 84 **(0.94)**  - 1156 **(1)**  + 1772 **(0.97)**  + 933 **(0.95)**  +/- 1057 **(1)**  +/- 1116  **(0.96)**  +/- 1912 **(0.96)** | WUSCHEL-related homeobox 13  (WOX13) | *Arabidopsis thaliana* | Multicellular organism development. |  |
| C2H2 | - 1941 **(0.92)**  - 1946 **(0.92)** | Zinc finger (C2H2 type) | *Arabidopsis thaliana* | Regulation of transcription; response to chitin. |  |
| MYB-related | + 465 (**0.99**)  - 465 (**0.88**)  - 1333 (**0.90**) | CIRCADIAN CLOCK ASSOCIATED 1 (CCA1) | *Arabidopsis thaliana* | Circadian rhythm; long-day photoperiodism, flowering; negative regulation of circadian rhythm; positive and negative regulation of transcription; regulation of gene expression and protein homodimerization activity; response to ABA, auxin, cadmium ion, cold, ethylene, GA, jasmonic acid, organonitrogen compound, salicylic acid, salt stress. |  |
| Myb/SANT; MYB; G2-like | + 134 (**0.91**) | KANADI 1 (KAN1) | *Arabidopsis thaliana* | Abaxial cell fate specification; adaxial/abaxial axis specification; carpel development; plant organ morphogenesis; plant ovule development; polarity specification of adaxial/abaxial axis;  radial pattern formation; regulation of transcription, DNA-templated; xylem and phloem pattern formation. |  |
| Myb/SANT; G2-like | - 135 **(0.94)**  +131 **(0.92)**  + 734  + 1025 **(0.88)**  - 1295 **(0.91)** | KAN2 | *Arabidopsis thaliana* | Carpel development; cell differentiation;  plant ovule development; polarity specification of adaxial/abaxial axis; regulation of transcription. | |
| bZIP | + 965 **(0.67)** | Opaque2 | *Zea mays* | DNA-binding transcription factor activity. |  |
| bHLH | +/- 6 (**0.75**)  +/- 792 (**0.88**) | BHLH78 | *Arabidopsis thaliana* | Regulation of transcription;  response to blue light. |  |
| bZIP | - 1094 **(0.75)** | ABF3 | *Arabidopsis thaliana* | ABA-activated signaling pathway;  response to ABA, salt stress, water deprivation. |  |
| CSD | - 444 (**0.97**)  + 660 (**1**)  - 727 (**1**) | Cold shock domain-containing protein 4 | *Arabidopsis thaliana* | Embryo development ending in seed dormancy; fruit development; response to cold. |  |
| TCR; CPP | - 447 **(0.99)**  + 701 **(0.99)**  - 921 **(0.98)** | Protein tesmin/TSO1-like CXC 3 | *Arabidopsis thaliana* | Stomatal complex patterning; stomatal lineage progression.  Plays a role in development of both male and female reproductive tissues. |  |
| TCR; CPP; CPP | - 448 **(0.98)**  + 700 **(0.97)** | Protein  tesmin/ TSO1-like CXC 5 | *Arabidopsis thaliana* | Multicellular organism development; regulation of transcription. |  |
| Dof | + 18 **(1)**  + 1569 **(1)** | DOF2.5  DOF3.7 | *Arabidopsis thaliana* | Cellular response to red light and water stimulus; positive regulation of GA biosynthetic process; positive regulation of seed germination; red light signaling pathway; response to cold; response to light stimulus; seed germination. Transcription factor specifically involved in the maternal control of seed germination. Regulates transcription by binding to a 5'-AA[AG]G-3' consensus core sequence. May ensure the activation of a component that would trigger germination due to red light perception. Both zinc finger proteins (DOF 2.7 and 3.7) may act on a maternal switch that controls seed germination, possibly by regulating the same gene(s). |  |
| Dof | + 977 **(1)** | DOF3.2; DOF6 | *Arabidopsis thaliana* | Regulation of transcription; root radial pattern formation. |  |
| Dof | - 782 **(1)**  - 941 **(1)** | DOF4.7 | *Arabidopsis thaliana* | Floral organ abscission; positive regulation of transcription; regulation of transcription. |  |
| Dof | + 18 **(1)**  +182 **(1)**  +927 **(1)**  + 1569 **(1)**  + 1740 **(1)** | DOF5.1 | *Arabidopsis thaliana* | Polarity specification of adaxial/abaxial axis; regulation of transcription, DNA-templated; root radial pattern formation. Transcription factor that binds specifically to a 5'-AA[AG]G-3' consensus core sequence (By similarity). Binds to 5'-TAAAGT-3' motif in REV promoter to triggers its transcription, thus regulating adaxial-abaxial polarity and influencing leaf axial patterning in an auxin transport- and response-dependent manner (IAA6 and IAA19 genes expression) Probably involved in early processes for vascular development. The PEAR proteins (DOF2.4, DOF5.1, DOF3.2, DOF1.1, DOF5.6 and DOF5.3) activate gene expression that promotes radial growth of protophloem sieve elements. |  |
| Dof | - 782 **(1)** | Dof zinc finger protein DOF5.6 | *Arabidopsis thaliana* | Phloem or xylem histogenesis; procambium histogenesis; root radial pattern formation; positive regulation of transcription. |  |
| EIN3; EIL | + 494 **(0.99)**  + 1241 **(0.98)** | EIL1; AtEIL1 | *Arabidopsis thaliana* | Ethylene-activated signaling pathway; regulation of sulfur metabolic process; regulation of transcription;  defense response to bacterium; response to ethylene |  |
| EIN3; EIL | - 492 **(0.96)**  - 1621 | EIL3 | *Arabidopsis thaliana* | Ethylene-activated signaling pathway; regulation of sulfur metabolic process; regulation of transcription. |  |
| EIN3; EIL | +/- 480 **(0.99)**  - 487 **(0.91)**  + 706 **(0.98)**  +/- 1102 **(0.99)**  +/- 1136 **(0.99)**  +/- 1221 **(0.99)**  - 1293 **(0.87)** | ETHYLENE INSENSITIVE 3  (EIN3) | *Arabidopsis thaliana* | Defense response to bacterium; ethylene-activated signaling pathway; regulation of L-ascorbic acid biosynthetic process; sugar mediated signaling pathway; regulation of transcription;  response to hypoxia, ethylene. |  |
| GATA; tify | + 399 **(0.95)**  - 740 **(0.95)** | GATA6 | *Arabidopsis thaliana* | Cell differentiation; positive regulation of transcription; response to light stimulus. |  |
| Homeodomain HD-ZIP | + 82 **(0.97)**  - 90  - 476  + 479  - 893  - 1136  + 1154 **(0.97)**  - 1221  - 1774 **(0.96)** | Homeobox-Leucine zipper protein  ATHB-4 | *Arabidopsis thaliana* | Regulation of transcription; response to far red light, hormone;  shade avoidance. |  |
| Homeodomain  HD-ZIP | + 84 **(0.95)**  + 1154 **(0.95)**  - 1774 **(0.92)** | ATHB-7 | *Arabidopsis thaliana* | Positive regulation of transcription.  Response to ABA.  Response to water deprivation. |  |
| Homeodomain bZIP; HD-ZIP; | + 82 **(0.93)**  + 1154 **(0.93)**  +/- 1910 **(0.93)** | ATHB-12 | *Arabidopsis thaliana* | Multicellular organism development; positive regulation of transcription; response to ABA, osmotic stress, viruses, water deprivation. |  |
| bZIP; HomeodomainHD-ZIP | + 1913 **(0.91)** | ATHB15; CNA; ICU4 | *Arabidopsis thaliana* | Determination of bilateral symmetry, dorsal identity; integument development; leaf morphogenesis; meristem initiation and maintenance; phloem or xylem histogenesis; plant organ morphogenesis; regulation of meristem growth. |  |
| Homeodomain; HD-ZIP; HD-ZIP | + 1059 **(0.95)**  + 1910 **(0.95)** | Homeobox-leucine zipper protein ANTHOCYANINLESS 2 (ANL2) | *Arabidopsis thaliana* | Anthocyanin accumulation in tissues in response to UV light; cuticle development; plant-type cell wall modification;  root development;  root hair cell differentiation. |  |
| Homeodomain; TALE | - 201 **(1)**  - 907 **(0.99)** | Homeobox protein knotted-1-like 3 (Protein KNAT3) | *Arabidopsis thaliana* | Cellular response to cytokine stimulus;  detection of cytokinin stimulus;  response to light stimulus |  |
| Homeodomain | - 468 **(0.94)**  + 1064 **(0.94)**  - 1066 **(1)** | Pathogenesis-related homeodomain protein (PRH1) | *Petroselinum crispum* | Defense response;  regulation of transcription. |  |
| Myb/SANT; ARR-B | - 1146 (**0.98**) | Two-component response regulator ARR1 | *Arabidopsis thaliana* | Axillary shoot meristem initiation; cellular response to cytokinin stimulus; cytokinin-activated signaling pathway; maintenance of shoot apical meristem identity; primary root development; regulation of anthocyanin metabolic process, chlorophyll biosynthetic process, cytokinin-activated signaling pathway, root meristem growth, seed growth; response to cytokinin, water deprivation; root development; shoot system development. |  |
| Myb/SANT; G2-like | - 1146 **(1)** | Protein PHOSPHATE STARVATION RESPONSE 1  (PHR1) | *Arabidopsis thaliana* | Cellular response to high light intensity;  cellular response to phosphate starvation; circadian rhythm;  regulation of transcription; sulfate ion homeostasis. |  |
| Myb/SANT | - 960 **(0.92)**  + 1515 **(0.93)**  - 1519 **(1)** | Transcription factor SRM1 | *Arabidopsis thaliana* | abscisic acid-activated signaling pathway; negative regulation of response to salt stress; positive regulation of abscisic acid biosynthetic process, developmental vegetative growth, and  regulation of transcription, DNA-templated; regulation of abscisic acid-activated signaling pathway, leaf morphogenesis, and transcription, DNA-templated; response to gibberellin, salt stress, salicylic acid, water deprivation |  |
| NAC; NAM | - 311 (**1**)  - 1721 (**1**) | NAC transcription factor 29  (NAC029) | *Arabidopsis thaliana* | embryo development ending in seed dormancy; flower development;  fruit ripening; leaf senescence;  multidimensional cell growth |  |
| NAC; NAM | + 619 **(0.79)**  + 938 **(0.78)**  - 1007 **(0.78)**  + 1014 **(0.78)**  - 1937 **(0.79)** | Protein NTM1-like 9  (NTL9) | *Arabidopsis thaliana* | Cellular response to osmotic stress;  negative regulation of transcription, DNA-templated; positive regulation of defense response to bacterium;  regulation of defense response;  regulation of transcription, DNA-templated |  |
| YABBY | - 1916 (**0.92**)  - 1981 (**0.93**) | Axial regulator YABBY 1 | *Arabidopsis thaliana* | Abaxial cell fate specification; fruit development; cell fate commitment; inflorescence meristem growth; meristem structural organization; polarity specification of adaxial/abaxial axis; regulation of flower development, leaf development, shoot apical meristem development; specification of floral organ identity and plant organ position. |  |
| Sox; YABBY | - 1058 (**1**)  + 1115 (**1**)  - 1913 (**1**) | Axial regulator YABBY 4  (YAB4) | *Arabidopsis thaliana* | Cell fate commitment; plant ovule development; polarity specification of adaxial/abaxial axis. |  |
| YABBY | - 962 (**0.97**) | CRABS CLAW | *Arabidopsis thaliana* | Carpel development; cell fate commitment; floral meristem determinacy; leaf development; meristem determinacy; nectary development; polarity specification of adaxial/abaxial axis; regulation of transcription; style development. |  |
| Storekeeper | + 512 (**0.97**)  - 512 (**0.96**) | GLABROUS1 enhancer-binding protein  (GEBP) | *Arabidopsis thaliana* | defense response to oomycetes;  regulation of transcription, DNA-templated |  |
| Storekeeper | - 1517 **(0.91)** | STKL2 | *Arabidopsis thaliana* | Cellular response to glucose stimulus; negative regulation of gene expression; regulation of transcription. | |
| TCP | + 6 **(1)**  - 9 **(1)**  + 167 **(0.95)**  - 168 **(1)** | Transcription factor TCP20  (TCP20) | *Arabidopsis thaliana* | anatomical structure morphogenesis; negative regulation of leaf senescence;  regulation of cell size | |
| TCP | + 706 **(0.97)** | Transcription factor TCP24 | *Arabidopsis thaliana* | Cell differentiation; leaf development and morphogenesis; negative regulation of cell population proliferation; positive regulation of development, heterochronic; regulation of transcription, DNA-templated. Plays a pivotal role in the control of morphogenesis of shoot organs by negatively regulating the expression of boundary-specific genes such as CUC genes, probably through the induction of miRNA (miR164). In association with ABAP1, exerts a negative role in cell proliferation in leaves, possibly by inhibiting mitotic DNA replication. Participates in ovule development. |  |
| TCP | - 185 **(1)**  - 204 **(1)**  + 314 **(1)**  - 910 **(1)**  - 988 **(1)**  - 1092 **(1)**  - 1636 **(1)**  + 1707 **(1)**  + 1724 **(1)**  + 1844 **(1)**  - 1896 **(1)** | Homeobox protein knotted-1-like 1  (KNAT1) | *Arabidopsis thaliana* | Cell fate commitment; cell fate specification; xylem and phloem pattern formation; xylem development. May play a role in meristem function and may be involved in maintaining cells in an undifferentiated, meristematic state, and its expression disappears at the same time the shoot apex undergoes the transition from vegetative to reproductive development. Positive regulator of LATERAL ORGAN BOUNDARIES. Probably binds to the DNA sequence 5'-TGAC-3'. Able to traffic from the L1 to the L2/L3 layers of the meristem, presumably through plasmodesmata. |  |
| TCP | - 185 **(1)**  - 204 **(1)**  + 314 **(1)**  - 910 **(1)**  - 988 **(1)**  - 1092 **(1)**  - 1636 **(1)**  + 1707 **(1)**  + 1724 **(1)**  + 1844 **(1)**  - 1896 **(1)** | Homeobox protein knotted-1-like 2  (KNAT2) | *Arabidopsis thaliana* | cytokinin-activated signaling pathway; specification of carpel identity.  May play a role in meristem function and may be involved in maintaining cells in an undifferentiated, meristematic state. Probably binds to the DNA sequence 5'-TGAC-3'. |  |
| TCP | - 185 **(1)**  - 204 **(1)**  + 314 **(1)**  - 910 **(1)**  - 988 **(1)**  - 1092 **(1)**  - 1636 **(1)**  + 1707 **(1)**  + 1724 **(1)**  + 1844 **(1)**  - 1896 **(1)** | Homeobox protein SHOOT MERI-STEMLESS  (STM) | *Arabidopsis thaliana* | Carpel development; floral meristem determinacy; regulation of meristem structural organization; cytokinin biosynthetic process;  plasmodesmata-mediated intercellular transport;  stem cell population maintenance.  Required for shoot apical meristem (SAM) formation during embryogenesis.  Negatively regulates ASYMMETRIC LEAVES1 (AS1) and ASYMMETRIC LEAVES2 (AS2 or LBD6).  Probably binds to the DNA sequence 5'-TGAC-3'.  Binds to RNA. |  |
| TCP | - 185 **(1)**  - 204 **(1)**  + 314 **(1)**  - 910 **(1)**  - 988 **(1)**  - 1092 **(1)**  - 1636 **(1)**  + 1707 **(1)**  + 1724 **(1)**  + 1844 **(1)**  - 1896 **(1)** | Homeobox protein knotted-1-like 6  (KNAT6) | *Arabidopsis thaliana* | Meristem maintenance.  Plays a role in meristem function.  Contributes to the shoot apical meristem (SAM) maintenance and organ separation by controlling boundary establishment in embryo in a CUC1, CUC2 and STM-dependent manner. Involved in maintaining cells in an undifferentiated, meristematic state.  Probably binds to the DNA sequence 5'-TGAC-3'. |  |
| WRC; GRF | - 1836 **(1)** | GRF6 | *Arabidopsis thaliana* | Leaf development; developmental process; transcription;  regulation of transcription. |  |
| Dof | - 348 **(1)**  - 555 **(1)**  - 678 **(1)**  - 939 **(0.98)**  -1318 **(1)** | Dof zinc finger protein MNB1A | *Zea mays* | Regulation of transcription. |  |
| SBP | - 177 **(1)**  + 178 **(1)**  - 227 **(1)**  + 228 **(1)**  - 641 **(1)**  + 642 **(1)**  - 764 **(1)**  + 765 **(1)**  - 1598 **(1)**  + 1599 **(1)**  - 1742 **(1)**  + 1743 **(1)** | Squamosa promoter-binding-like protein 3  (SPL3) | *Arabidopsis thaliana* | cell differentiation; flower development; inflorescence development; positive regulation of flower development;  regulation of vegetative phase change; vegetative to reproductive phase transition of meristem.  Trans-acting factor that binds specifically to the consensus nucleotide sequence 5'-TNCGTACAA-3' of APETALA1 promoter. Binds specifically to the 5'-GTAC-3' core sequence. Promotes both vegetative phase change and flowering. Regulates phase-specific patterns of leaf epidermal differentiation and flowering time but does not seem to affect leaf shape. |  |
| SBP | + 172 **(0.96)**  - 759 **(0.96)**  + 1737 **(0.94)** | Squamosa promoter-binding-like protein 8 (SPL8) | *Arabidopsis thaliana* | Anther development; cell differentiation; megasporogenesis;  microsporogenesis. Trans-acting factor that binds specifically to the consensus nucleotide sequence 5'-TNCGTACAA-3'. Binds specifically to the 5'-GTAC-3' core sequence. Involved in development and floral organogenesis. Required for ovule differentiation, pollen production, filament elongation, seed formation and silique elongation. Also seems to play a role in the formation of trichomes on sepals. May positively modulate GA signaling in flowers. |  |
| AP2; ERF | + 1437 (**0.99**) | Ethylene-responsive transcription factor RAP2-6 | *Arabidopsis thaliana* | Cellular response to heat; chloroplast organization; defense response; ethylene-activated signaling pathway; positive regulation of transcription; response to ABA, cold, jasmonic acid, osmotic stress, salicylic acid, salt stress, water deprivation, wounding. |  |
| Dof | + 1640 **(0.99)**  - 976 **(0.88)** | Dof zinc finger protein DOF5.7 | *Arabidopsis thaliana* | Guard cell differentiation; positive regulation of transcription; regulation of cell wall pectin metabolic process; regulation of transcription; stomatal movement. |  |
| HSF | + 887 **(0.88)**  + 1250 **(0.88)**  - 1670 **(0.88)** | Heat stress transcription factor C-1 (HSFC1) | *Arabidopsis thaliana* | Cellular response to heat; positive regulation of transcription from RNA polymerase II promoter in response to heat stress; transcription from RNA polymerase II promoter in response to stress. |  |
| Alpha-amylase | - 1371 **(0.8)** | Glucan endo-1,3-beta-glucosidase, basic vacuolar isoform GGIB50 | *Arabidopsis thaliana;*  *Nicotiana tabacum* | Carbohydrate metabolic process;  defense response. |  |
| bZIP | - 984 **(0.7)** | Transcription factor TGA2 | *Arabidopsis thaliana* | Negative regulation of transcription; plant-type hypersensitive response; positive regulation of transcription; response to xenobiotic stimulus; regulation of systemic acquired resistance, salicylic acid mediated signaling pathway; transcription. |  |
| bZIP | - 643 **(0.7)** | Transcriptional activator TAF-1 | *Nicotiana tabacum* | Regulation of transcription. |  |
| bZIP | - 1631 **(1)**  + 1632 **(1)** | BZIP10 | *Arabidopsis thaliana* | Plant-type hypersensitive response; positive regulation of seed maturation; positive regulation of transcription; response to molecule of oomycetes origin.  Transcription factor that binds to the C-box-like motif (5'-TGCTGACGTCA-3') and G-box-like motif (5'-CCACGTGGCC-3'), ABRE elements, of gene promoters. Binds to the 5'-ACGT-3' motif of seed storage protein (SSP) encoding gene promoters (e.g. At2S and CRU3) and promotes their expression in seeds when in complex with ABI3 and BZIP53. Involved in the defense responses to the biotrophic pathogen Hyaloperonospora parasitica and oxidative stress responses; mediates positively cell death. Promotes BZIP53-mediated response to hypo-osmolarity stress that leads to POX1/PRODH1 accumulation. |  |
| bZIP | + 1663 **(1)** | VIP1 | *Arabidopsis thaliana* | Cellular response to sulfate starvation; defense response; DNA mediated transformation; import into nucleus; negative regulation of cell differentiation; osmo-sensory signaling pathway; response to osmotic stress; sulfate transport;  thigmotropism. Transcription activator that binds specifically to the VIP1 response elements DNA sequence 5'-ACNGCT-3' found in some stress genes (TRX8 and MYB44), when phosphorylated/activated by MPK3. Required for Agrobacterium VirE2 nuclear import and tumorigenicity. Promotes transient expression of T-DNA in early stages by interacting with VirE2 in complex with the T-DNA and facilitating its translocation to the nucleus and mediates stable genetic transformation by Agrobacterium by binding H2A histone. Prevents cell differentiation and shoot formation. Limits sulfate utilization efficiency and sulfate uptake, especially in low-sulfur conditions. |  |
| LEA type 1 | - 643 **(0.8)** | ABA-inducible protein PHV A1 (HVA1) | *Hordeum vulgare* |  |  |
| (Others) | - 1167  - 1709 | Zein protein 3 | *Zea mays* |  |  |
| Lyase  -aromatic | + 139 **(0.79)** | PAL1 | *Petroselinum hortense* | Cinnamic acid biosynthetic process;  L-phenylalanine catabolic process. |  |
| ARID | + 990 (**0.95**)  - 1059 (**0.93**) | ARID6 | *Arabidopsis thaliana* | Chromatin silencing; glucosinolate metabolic process;  regulation of transcription. |  |
| ARID; Sox | - 462 **(0.93)** | High mobility group B protein 15 (HMGB15) | *Arabidopsis thaliana* | Glucosinolate metabolic process; pollen germination; pollen tube growth; regulation of transcription. |  |
| ARID; Sox | + 989 **(0.98)**  - 1060 **(0.99)**  - 1911 **(0.95)** | HMGB9 | *Arabidopsis thaliana* | Karyogamy;  polar nucleus fusion;  regulation of transcription, |  |
| ARID | - 992 **(0.94)** | ARID3 | *Arabidopsis thaliana* | Chromatin silencing;  regulation of transcription. |  |
| bHLH | - 642 **(0.99)**  - 1689 **(0.99)** | BHLH122 | *Arabidopsis thaliana* | Cuticle development; photoperiodism, flowering; regulation of stomatal movement; regulation of transcription. |  |
| bHLH | - 643 **(0.92)**  - 1690 **(0.89)** | BHLH80 | *Arabidopsis thaliana* | Cuticle development;  regulation of transcription. |  |
| bHLH | - 643 **(0.99)**  - 1690 **(0.98)** | BHLH130 | *Arabidopsis thaliana* | Photoperiodism, flowering;  regulation of transcription. |  |
| bHLH | + 1615 **(1)** | HBI1 | *Arabidopsis thaliana* | Brassinosteroid- and GA_3_-mediated signaling pathways; regulation of growth. |  |
| Dof | + 182 **(1)**  + 1569 **(1)** | CDF1 | *Arabidopsis thaliana* | Chloroplast organization; flower development; negative regulation of transcription; regulation of timing of transition from vegetative to reproductive phase; regulation of transcription; vegetative to reproductive phase transition of meristem. Transcription factor that binds specifically to a 5'-AA[AG]G-3' consensus core sequence. A flanking TGT sequence contributes to the specificity of binding. Regulates a photoperiodic flowering response. Transcriptional repressor of 'CONSTANS' expression. The DNA-binding ability is not modulated by 'GIGANTEA' but the stability of CDF1 is controlled by the proteasome-dependent pathway. Ubiquitinated by the SCF(ADO3) E3 ubiquitin ligase complex. Binds to the FT promoter in the morning. |  |
| Dof | + 748 (**0.86**)  - 752 (**0.92**) | Cyclic Dof factor 5 (CDF5) | *Arabidopsis thaliana* | Flower development; negative regulation of long-day photoperiodism, flowering, short-day; photoperiodism, flowering;  regulation of transcription. |  |
| GATA; tify | - 741 **(0.9)** | GATA12 | *Arabidopsis thaliana* | Cell differentiation; cell wall organization; circadian rhythm;  multicellular organism development; positive regulation of transcription; response to light stimulus; tracheary element differentiation. |  |
| GATA | - 741 **(1)** | GATA1 | *Arabidopsis thaliana* | Cell differentiation; circadian rhythm. |  |
| GATA | - 737 **(0.91)** | GATA14 | *Arabidopsis thaliana* | Cell differentiation; regulation of transcription. |  |
| C2H2 | + 183 **(0.94)**  - 1002 **(1)**  - 1071 **(0.97)** | Zinc finger protein JAGGED-like | *Arabidopsis thaliana* | Anther development; carpel development; stamen development;  cell differentiation. |  |
| C3H | + 306 (**0.83**)  + 394 (**1**)  + 1264 (**0.83**)  + 1267 (**0.83**) | Splicing factor U2af small subunit B (U2AF35B) | *Arabidopsis thaliana* | mRNA splicing, via spliceosome;  photoperiodism; flowering. |  |
| C3H | - 1758 **(0.83)** | At1g70910  (DEP) | *Arabidopsis thaliana* | Response to misfolded protein; seed dormancy process;  ubiquitin-dependent protein catabolic process. |  |
| NF-YB | - 630 **(1)**  - 1835 **(1)** | NFYB4 | *Arabidopsis thaliana* | Positive regulation of transcription;  regulation of transcription. |  |
| Myb/SANT | - 1146 **(1)**  +/- 1294 **(0.95)** | Protein PHR1-LIKE 2  (PHL2) | *Arabidopsis thaliana* | Cellular response to phosphate starvation; positive regulation of gene expression; regulation of transcription. |  |
| Myb/SANT | - 1143 **(0.98)**  - 1147 **(1)**  - 1292 **(0.95)** | Myb-related protein 2  (MYR2) | *Arabidopsis thaliana* | Transcription of specific genes involved in nitrogen uptake or assimilation; repressor of flowering and organ elongation under decreased light intensity; represses GA_3_-dependent responses and affects levels of bioactive GA_3_. |  |
| Myb/SANT | + 1140 **(0.96)**  - 1146 **(1)**  + 1147 **(0.99)** | HHO2 | *Arabidopsis thaliana* | Cellular response to hypoxia; phosphate ion homeostasis; regulation of transcription; response to ABA. |  |
| Myb/SANT | - 1143 **(1)**  + 1140 **(1)** | HHO3 | *Arabidopsis thaliana* | Cellular response to hypoxia; regulation of transcription. |  |
| Myb/SANT; G2-like | + 1143 **(0.95)** | HHO5 | *Arabidopsis thaliana* | Floral organ formation; negative regulation of gene expression; regulation of transcription; specification of plant organ identity. |  |
| Myb/SANT; G2-like | + 1139 **(0.95)**  + 1142 **(0.97)** | HHO6 | *Arabidopsis thaliana* | Regulation of transcription. Probable transcription factor involved in phosphate signaling in roots. |  |
| Myb/SANT | + 1139 **(0.98)**  - 1146 **(1)** | HRS1 | *Arabidopsis thaliana* | Cellular responses to hypoxia, nitrogen compound, phosphate starvation; negative regulation of ABA-activated signaling pathway; primary root development; regulation of response to nutrient levels; regulation of transcription. |  |
| Storekeeper | - 1517 **(0.91)** | STKL2 | *Arabidopsis thaliana* | Cellular response to glucose stimulus; negative regulation of gene expression; regulation of transcription. |  |
| WRC; GRF | + 903 **(0.93)**  + 1629 **(0.99)** | GRF6 | *Arabidopsis thaliana* | Leaf development; regulation of transcription; transcription. |  |
| GRF | + 910 **(0.96)**  + 1636 **(1)** | GRF9 | *Arabidopsis thaliana* | Leaf development; regulation of transcription; transcription. |  |
| HD-ZIP | - 1910 **(0.9)**  - 1804 **(0.73)** | HDG7 | *Arabidopsis thaliana* | DNA-binding transcription factor activity; lipid binding. |  |
| Homeodomain; HD-ZIP | - 1058 **(0.81)**  - 1910 **(0.9)** | HDG1 | *Arabidopsis thaliana* | Maintenance of floral organ identity. |  |
| HD-ZIP | - 1295 **(0.84)** | PDF2 | *Arabidopsis thaliana* | Cotyledon development; epidermal cell differentiation; maintenance of floral organ identity; seed germination. |  |
| HSF | - 375 **(1)** | Heat stress transcription factor A-1b  (HSFA1B) | *Arabidopsis thaliana* | Cellular response to heat; positive regulation of transcription from RNA polymerase II promoter in response to heat stress; regulation of transcription from RNA polymerase II promoter in response to stress; response to heat. |  |
| Myb/SANT; MYB | + 955  - 1518 | At1g49010 | *Arabidopsis thaliana* | Positive regulation of transcription; response to auxin, cadmium ion, GA, jasmonic acid, salicylic acid, salt stress, sucrose. |  |
| Myb/SANT; MYB; MYB-related; MYB | - 319 **(0.93)**  + 1394 **(0.93)** | AtMYB3 | *Arabidopsis thaliana* | Cell differentiation; cinnamic acid biosynthetic process; negative regulation of metabolic process; response to ABA, salicylic acid, salt stress, wounding. |  |
| MYB | + 41 **(0.91)**  + 509 **(0.9)**  - 590 **(0.9)**  - 928 **(0.93)** | MYB3R-1 | *Arabidopsis thaliana* | Negative regulation of cell population proliferation, cellular response to caffeine; positive and negative regulation of transcription; regulation of cytokinesis. |  |
| Myb/SANT; MYB | + 40 **(0.9)** | MYB3R-4 | *Arabidopsis thaliana* | Defense response to fungus; negative regulation of cellular response to caffeine; positive regulation of transcription; regulation of cytokinesis, DNA endoreduplication and transcription; response to fungus and salicylic acid. |  |
| Myb/SANT; MYB | + 39 **(0.89)** | MYB3R-5 | *Arabidopsis thaliana* | Negative regulation of cell population proliferation, transcription;  response to ethylene, salicylic acid. |  |
| Myb/SANT; MYB; MYB-related; MYB | - 321 **(0.95)** | MYB4 | *Arabidopsis thaliana* | Cell differentiation; regulation of phenylpropanoid metabolic process; negative regulation of sinapate ester biosynthetic process, transcription; response to jasmonic acid, salicylic acid, UV-B. |  |
| Myb/SANT; MYB | + 318 **(0.94)**  - 319 | MYB10 | *Arabidopsis thaliana* | Cell differentiation; regulation of gene expression; response to absence of light, cadmium ion, ethylene, iron ion starvation, zinc ion; stress response to nickel ion. |  |
| Myb/SANT; MYB | + 1390 **(0.88)** | MYB17 | *Arabidopsis thaliana* | Cell differentiation; flower development; regulation of flower development; response to jasmonic acid, salicylic acid. |  |
| MYB | + 302 **(0.99)**  + 1260 **(0.99)**  + 1263 **(0.99)** | MYB23 | *Arabidopsis thaliana* | Cell differentiation; regulation of transcription; trichome branching and differentiation; root epidermal cell differentiation. |  |
| Myb/SANT; MYB-related; | + 365 **(0.99)**  + 375 **(1)**  + 1348 **(1)** | MYB28 | Oryza sativa subsp. japonica | Chromosome condensation; negative regulation of chromatin silencing, DNA recombination; nucleosome assembly and positioning; regulation of transcription. |  |
| Myb/SANT | - 932 (**0.98**) | MYB33 | *Arabidopsis thaliana* | Anther development; cell differentiation; anther wall tapetum morphogenesis; pollen sperm cell differentiation; GA_3_-mediated signaling pathway; positive regulation of programmed cell death, ABA-activated signaling pathway, transcription; response to cytokinin, ethylene, GA; protein storage vacuole organization;  negative regulation of cell population proliferation, growth. |  |
| MYB | - 318 **(0.89)** | MYB39 | *Arabidopsis thaliana* | Cell differentiation. |  |
| Myb/SANT; MYB | + 319 **(0.96)** | MYB40 | *Arabidopsis thaliana* | Cell differentiation. |  |
| Myb/SANT; MYB | + 315 **(0.91)** | MYB41 | *Arabidopsis thaliana* | Cell differentiation; multidimensional cell growth; cuticle development; negative regulation of transcription; regulation of primary metabolic process; regulation of raffinose metabolic process; response to ABA, chitin. |  |
| Myb/SANT; MYB | - 318 **(0.92)** | MYB43 | *Arabidopsis thaliana* | Cell differentiation; regulation of secondary cell wall biogenesis; response to ABA. |  |
| Myb/SANT; MYB | - 933 (**0.97**) | MYB44 | *Arabidopsis thaliana* | ABA-activated signaling pathway; defense responses to bacteria and fungi; positive regulation of auxin mediated signaling pathway; regulation of defense response to fungus; regulation of jasmonic acid mediated signaling pathway, salicylic acid mediated signaling pathway, transcription; response to ABA, auxin, cadmium ion, chitin, ethylene, GA, jasmonic acid, salicylic acid, salt stress, water deprivation. |  |
| MYB | + 1488 **(0.90)**  - 594 **(0.94)** | MYB 46 | *Arabidopsis thaliana* | Cell differentiation; defense response to fungus; plant-type secondary cell wall biogenesis; positive regulation of secondary cell wall biogenesis, transcription; regulation of secondary cell wall biogenesis; response to salicylic acid. |  |
| Myb/SANT; MYB | + 316 **(0.9)**  - 318 **(0.91)** | MYB 49 | *Arabidopsis thaliana* | Cell differentiation; cellular cadmium ion homeostasis; positive regulation of gene expression; regulation of transcription; responses to ABA, salicylic acid, salt stress. |  |
| MYB | + 309 **(0.94)**  - 1201 **(0.94)**  - 1394 **(0.94)** | MYB51 | *Arabidopsis thaliana* | Cell differentiation; defense response by callose deposition in cell wall; defense response to bacterium; indole glucosinolate biosynthetic process; induced systemic resistance; response to ABA, auxin, bacterium, ethylene, GA, insect, jasmonic acid, salicylic acid, and salt stress. |  |
| MYB | - 47 **(0.90)**  + 587 **(0.89)** | MYB 52 | *Arabidopsis thaliana* | Cell wall organization; negative regulation of secondary cell wall biogenesis; plant-type secondary cell wall biogenesis; positive regulation of secondary cell wall biogenesis; response to ABA, salt, water deprivation. |  |
| MYB | - 1116 **(0.86)**  - 1119 **(0.92)**  - 1234 **(0.93)** | MYB 57 | *Arabidopsis thaliana* | GA_3_- and jasmonic acid-mediated signaling pathway; stamen development; stamen filament development; regulation of transcription. |  |
| MYB; MYB-related | + 1233 **(0.93)** | MYB59 | *Arabidopsis thaliana* | Cellular response to potassium ion; regulation of potassium ion transport; regulation of transcription; response to cadmium ion, chitin, ethylene, GA, jasmonic acid, salicylic acid. |  |
| Myb/SANT; MYB | + 1114 **(0.96)**  - 1117 **(0.91)** | MYB 62 | *Arabidopsis thaliana* | Cellular response to phosphate starvation; GA_3_-mediated signaling pathway; GA biosynthetic process; response to salicylic acid; negative regulation of GA biosynthetic process, transcription; phosphate ion homeostasis; regulation of transcription; root morphogenesis. |  |
| Myb/SANT | - 928 (**0.98**)  - 933 (**0.99**) | MYB 65 | *Arabidopsis thaliana* | Anther development; anther wall tapetum morphogenesis; cell differentiation; positive regulation of auxin mediated signaling pathway; negative regulation of cell population proliferation; negative regulation of growth; pollen sperm cell differentiation; positive regulation of programmed cell death; positive regulation of transcription; protein storage vacuole organization; regulation of transcription; response to ethylene; response to salicylic acid |  |
| Myb/SANT; MYB | - 931 **(0.99)** | MYB 73 | *Arabidopsis thaliana* | Glucosinolate metabolic process; negative regulation of response to salt stress; response to ABA, cadmium ion, chitin, ethylene, jasmonic acid, salicylic acid; positive regulation of auxin mediated signaling pathway; regulation of transcription. |  |
| Myb/SANT; MYB | + 318 **(0.95)**  - 318 **(0.9)**  + 1392 **(0.89)**  - 1392 **(0.97)**  + 1486 **(0.9)** | MYB74 | *Arabidopsis thaliana* | Cell differentiation;  responses to ABA, ethylene, jasmonic acid, salt stress. |  |
| Myb/SANT | + 927 **(0.96)**  - 1070 **(0.95)** | MYB77 | *Arabidopsis thaliana* | Lateral root development; positive regulation of auxin mediated signaling pathway; regulation of transcription;  response to chitin, ethylene, salicylic acid. |  |
| Myb/SANT; MYB | - 319  + 1484 | MYB 80 | *Arabidopsis thaliana* | Cell differentiation; pollen development; anther wall tapetum development; trichome morphogenesis. |  |
| Myb/SANT; MYB | - 932 **(0.96)** | MYB 81 | *Arabidopsis thaliana* | Cell differentiation |  |
| Myb/SANT; MYB | - 280 (**0.92**) | MYB 88 | *Arabidopsis thaliana* | Embryo sac development; guard cell and guard mother cell differentiation; guard mother cell cytokinesis; lateral root development; megasporogenesis; multicellular organismal water homeostasis; positive regulation of lateral root development; positive regulation of responses to salt stress and water deprivation; response to ABA, gravity; regulation of cell cycle G1/S phase transition, DNA endoreduplication, transcription, stomatal complex patterning. |  |
| Myb/SANT; MYB | + 312 **(0.93)**  - 319 **(0.89)** | MYB92 | *Arabidopsis thaliana* | Cell differentiation;  response to jasmonic acid and salicylic acid. |  |
| Myb/SANT; MYB | - 933 **(0.92)** | MYB 98 | *Arabidopsis thaliana* | Embryo sac development; regulation of embryo sac central cell differentiation; regulation of synergid differentiation; pollen tube guidance; regulation of transcription. |  |
| Myb/SANT; MYB | + 319 **(0.94)** | MYB 99 | *Arabidopsis thaliana* | Cell differentiation. |  |
| Myb/SANT; MYB | - 518 **(0.94)**  - 932 **(0.98)** | MYB 101 | *Arabidopsis thaliana* | Cell differentiation; GA_3_-mediated signaling pathway; response to GA; regulation of ABA-activated signaling pathway, programmed cell death, transcription; pollen development; pollen sperm cell differentiation; positive protein storage vacuole organization; regulation of gene expression, leaf morphogenesis, pollen tube growth. |  |
| Myb/SANT; MYB | - 313 **(0.9)**  + 316 **(0.91)** | MYB 107 | *Arabidopsis thaliana* | Cell differentiation;  response to salicylic acid. |  |
| MYB | + 1112 **(0.94)** | MYB108 | *Arabidopsis thaliana* | Defense response; positive regulation of anther dehiscence; regulation of transcription;  response to ABA, ethylene, fungus, jasmonic acid, salt stress. |  |
| Myb/SANT; MYB | - 320 (**0.93**)  + 1394 (**0.91**)  + 1488 (**0.90**) | MYB111 | *Arabidopsis thaliana* | Cell differentiation; flavonol biosynthetic process; positive regulation of flavonol biosynthetic process;  response to UV-B, light stimulus. |  |
| MYB | + 184 **(0.94)** | MYB113 | *Arabidopsis thaliana* | Cell differentiation; multicellular organism development; regulation of anthocyanin biosynthetic process; regulation of transcription; response to jasmonic acid. |  |
| Myb/SANT; MYB | + 1113 **(0.95)**  + 1117 **(0.88)** | MYB 116 | *Arabidopsis thaliana* | Regulation of transcription. |  |
| Myb/SANT; MYB | + 310 (**0.86**)  - 314 (**0.90**)  - 933 (**0.90**)  + 1320 (**0.80**)  - 1322 (**0.85**) | MYB 118 | *Arabidopsis thaliana* | SE. Endosperm development; vegetative to reproductive phase transition of meristem; fatty acid homeostasis; regulation of embryonic development, endosperm development, seed maturation; negative regulation of endosperm development, seed maturation; positive regulation of fatty acid biosynthetic process, unsaturated fatty acid biosynthetic process, transcription; regulation of glucosinolate biosynthetic process. |  |
| Myb/SANT; MYB | - 314 **(0.86)**  - 933 **(0.89)** | MYB 119 | *Arabidopsis thaliana* | Required for female gametophyte fertility;  initiates switch between free nuclear divisions and cellularization-differentiation in female gametophyte. |  |
| MYB-related | - 133 **(0.96)**  - 465 **(0.89)**  - 875 **(0.95)**  - 1333 **(0.99)**  - 1975 **(0.99)** | REVEILLE 1 (RVE1) | *Arabidopsis thaliana* | Auxin-activated signaling pathway;  circadian rhythm; regulation of auxin biosynthetic process; regulation of transcription. |  |
| Myb/SANT; MYB-related | + 466 (**0.87**)  + 1334 (**0.88**) | REVEILLE 4  RVE4 | *Arabidopsis thaliana* | Circadian rhythm; regulation of circadian rhythm;  response to ABA, auxin, cadmium ion, ethylene, GA, jasmonic acid, salicylic acid, salt stress. |  |
| Myb/SANT; MYB-related | - 1333 **(0.97)**  + 1334 **(0.89)**  - 1976 **(0.94)** | REVEILLE 5  (RVE5) | *Arabidopsis thaliana* | Response to ABA, auxin, ethylene, cadmium ion, GA, jasmonic acid, salicylic acid, salt stress. |  |
| Myb/SANT; MYB-related | + 466 **(0.87)**  + 1334 **(0.91)** | REVEILLE 6  (RVE6) | *Arabidopsis thaliana* | Regulation of circadian rhythm; response to ABA, cadmium ion, ethylene, GA, jasmonic acid, salicylic acid, and to salt stress. |  |
| Myb/SANT; MYB-related | + 134 **(0.98)**  - 135 **(0.97)**  - 466 **(0.95)**  + 876 **(0.98)**  - 877 **(0.96)**  - 1332 **(0.9)**  + 1333 **(1)**  - 1334 **(0.99)**  + 1976 **(0.99)**  - 1977 **(0.97)** | REVEILLE 7  (RVE7) | *Arabidopsis thaliana* | Circadian rhythm; regulation of transcription; response to cadmium ion, ethylene, salt stress.  Transcription factor involved in phytochrome A-mediated cotyledon opening.  Controlled by the central oscillator mediated by LHY and CCA1.  Part of a regulatory circadian feedback loop.  Regulates its own expression. |  |
| Myb/SANT; MYB-related | - 1333 (**0.99**)  + 1334 (**0.93**)  - 1976 (**0.99**) | REVEILLE 8  (RVE8) | *Arabidopsis thaliana* | Circadian regulation of gene expression; histone H3 acetylation; photoperiodism, flowering; positive regulation of circadian rhythm, gene expression, and transcription by RNA polymerase II; regulation of circadian rhythm; regulation of transcription;  response to ABA, auxin, cadmium ion, ethylene, GA, jasmonic acid, salicylic acid, salt stress. |  |
| Myb/SANT; MYB-related | + 1334 (**0.95**) | LHY | *Arabidopsis thaliana* | Circadian rhythm; long-day photoperiodism, flowering; negative regulation of circadian rhythm and DNA-binding transcription factor activity; regulation of circadian rhythm; regulation of transcription; response to abscisic acid, auxin, cadmium ion, cold, ethylene, GA, jasmonic acid, salicylic acid, salt stress. |  |
| Myb/SANT | + 1516 (**0.92**)  - 1517 (**0.94**)  - 1518 (**0.98**) | KUA1 | *Arabidopsis thaliana* | Auxin homeostasis; lateral root development; leaf development; leaf senescence; positive regulation of cell growth; post-embryonic plant organ morphogenesis; negative regulation of peroxidase activity, transcription, and transcription by RNA polymerase II; regulation of transcription; response to ABA, absence of light, auxin, cadmium ion, ethylene, GA, jasmonic acid, salicylic acid, salt stress, sucrose. |  |
| Myb/SANT | + 1431 **(0.93)**  - 1516 **(0.92)**  + 1517 | F25A4.19 | *Arabidopsis thaliana* | Response to ABA, auxin, cadmium ion, ethylene, GA, jasmonic acid, salicylic acid, salt stress. |  |
| Myb/SANT; MYB | - 958  + 1515 | At1g49010 | *Arabidopsis thaliana* | Positive regulation of transcription; response to auxin, cadmium ion, GA, jasmonic acid, salicylic acid, salt stress, sucrose. |  |
| MYB-related | - 377 **(0.99)** | Telomere repeat-binding factor 1  TRB1 |  | Nucleosome assembly;  regulation of transcription by RNA polymerase II;  response to ABA, auxin, cadmium ion, ethylene, GA, jasmonic acid, salicylic acid, salt stress. |  |
| Myb/SANT; MYB-related | - 363 (**0.92**)  + 367 (**1**)  + 377 (**0.97**)  - 1346 (**0.98**)  + 1347 (**0.97**)  + 1350 (**0.97**)  - 1420 (**0.98**) | Telomere repeat-binding factor 2 (TRB2) | *Arabidopsis thaliana* | Nucleosome assembly;  response to ABA, auxin, cadmium ion, ethylene, GA, jasmonic acid, salicylic acid, salt stress. |  |
| MYB-related | + 1340 **(0.99)**  - 1347 **(0.99)** | Telomere repeat-binding factor 5 | *Arabidopsis thaliana* | Nucleosome assembly. |  |
| Myb/SANT; MYB | + 955 **(0.96)**  - 955 **(0.89)**  + 958 **(0.94)**  - 960 **(1)**  - 972 **(0.93)**  + 974 **(0.99)**  + 1512 **(0.91)**  - 1516 **(0.95)**  + 1519 **(1)** | Duplicated homeodomain-like superfamily protein | *Arabidopsis thaliana* | Regulation of transcription;  response to GA, salicylic acid. |  |
| NAC | - 617  - 647 **(0.95)**  - 682  - 1790 **(0.88)**  + 1935 | NAC domain-containing protein 40  NTL8 | *Arabidopsis thaliana* | Cellular response to salt stress; flower development; regulation of trichome morphogenesis; seed germination; GA_3_-mediated signaling pathway; membrane protein proteolysis; negative regulation of GA_3_-mediated signaling pathway; photoperiodism; positive regulation of transcription; response to GA, salt stress. |  |
| NAC | + 617  - 617 **(0.91)**  - 619 **(0.95)**  - 1373  + 1935 **(0.93)**  - 1935 | NAC domain-containing protein 62  NAC062 | *Arabidopsis thaliana* | Cellular response to cold; response to chitin;  defense response, incompatible interaction;  positive regulation of endoplasmic reticulum unfolded protein response; regulation of transcription. |  |
| (Others) | + 703 **(1)**  - 923 **(1)**  + 1213 **(1)**  - 1360 **(1)** | Ethylene-responsive transcription factor ERF026 | *Arabidopsis thaliana* | Ethylene-activated signaling pathway. |  |
| Trihelix | + 15 **(1)**  + 355 **(0.96)**  + 357 **(1)**  - 1536 **(1)** | Trihelix transcription factor GTL1 | *Arabidopsis thaliana* | Cellular response to water deprivation; negative regulation of cell growth, DNA endoreduplication; transcription; regulation of cell size; regulation of stomatal complex development and patterning; response to water deprivation; transcription; trichome morphogenesis.  Transcription repressor that binds specific DNA sequence such as GT3 box 5'-GGTAAA-3' in the SDD1 promoter. Negative regulator of water use efficiency via the promotion of stomatal density and distribution by the transcription repression of SDD1. Regulates the expression of several cell cycle genes and endoreduplication, especially in trichomes where it prevents ploidy-dependent plant cell growth. |  |
| Trihelix | - 366 **(0.95)**  - 1349 **(0.97)** | Trihelix transcription factor ASR3 | *Arabidopsis thaliana* | Cellular response to molecule of bacterial origin; defense response; negative regulation of immune response; negative regulation of transcription; regulation of transcription. |  |
| MADF ; Trihelix | + 1046 **(0.87)**  - 1052 **(1)**  - 1107 **(0.91)** | Trihelix transcription factor GT-4 | *Arabidopsis thaliana* | Regulation of transcription. |  |
| MADF ; Trihelix | - 1045 **(0.83)**  + 1056 **(1)**  + 1109 **(0.89)** | Trihelix transcription factor GT-1 | *Arabidopsis thaliana* | Regulation of transcription.  May act as a molecular switch in response to light signals. |  |
| WRKY | + 1633 **(0.73)**  - 1719 | WRKY1 | *Arabidopsis thaliana* | Positive regulation of transcription; response to salicylic acid; salicylic acid mediated signaling pathway. |  |
| WRKY | + 311 **(0.86)**  + 1721 **(0.87)** | WRKY6 | *Arabidopsis thaliana* | Cellular response to boron-containing substance deprivation and to phosphate starvation; ethylene-activated signaling pathway; negative regulation of transcription; response to chitin. |  |
| WRKY | - 309 **(0.82)** | WRKY8 | *Arabidopsis thaliana* | Cellular response to hydrogen peroxide; response to ABA;  defense response to bacteria, fungi, viruses;  positive regulation of response to salt stress. |  |
| WRKY | -310 **(0.98)**  - 311 **(0.91)**  - 1721 **(0.9)** | WRKY15 | *Arabidopsis thaliana* | Response to chitin. |  |
| WRKY | + 311 **(0.95)**  + 1721 **(0.95)**  - 1721 **(0.97)** | WRKY18 | *Arabidopsis thaliana* | Defense response to bacterium and fungus; regulation of defense response; response to chitin, molecule of bacterial origin, salicylic acid. |  |
| WRKY | + 1719 **(0.93)** | WRKY20 | *Arabidopsis thaliana* | Positive regulation of transcription; response to 1-aminocyclopropane-1-carboxylic acid; response to wounding. |  |
| WRKY | - 311 **(0.85)** | WRKY21 | *Arabidopsis thaliana* | Glucosinolate metabolic process. |  |
| WRKY | - 309 **(0.85 0** | WRKY22 | *Arabidopsis thaliana* | Cellular response to hypoxia; defense response;  leaf senescence; response to chitin. |  |
| WRKY | + 312 **(0.98)**  - 1721 **(0.91)** | WRKY23 | *Arabidopsis thaliana* | Protein localization involved in auxin polar transport; response to auxin; response to nematode. |  |
| WRKY | - 311 **(0.93)**  - 1721 **(0.91)** | WRKY25 | *Arabidopsis thaliana* | Cellular heat acclimation; cellular response to heat;  response to cold, osmotic stress, salt stress. |  |
| WRKY | - 309 **(0.87)**  + 311 **(0.92)**  - 1719 **(0.86)**  + 1721 **(0.91)** | WRKY26 | *Arabidopsis thaliana* | Cellular heat acclimation;  cellular response to heat. |  |
| WRKY | + 309 **(0.94)** | WRKY28 | *Arabidopsis thaliana* | Regulation of cell fate specification. |  |
| WRKY | - 311 **(1)** | WRKY33 | *Arabidopsis thaliana* | Camalexin biosynthetic process; cellular heat acclimation; cellular response to heat; positive regulation of autophagy;  response to chitin, cold, heat, osmotic stress, salt stress, water deprivation; defense response to bacterium, fungus;  systemic acquired resistance. |  |
| WRKY | + 309 **(0.94)**  - 311 **(0.94)**  - 312 **(1)**  + 1719 **(0.93)**  - 1721 **(0.92)**  - 1722 **(1)** | WRKY40 | *Arabidopsis thaliana* | Defense response to bacterium;  defense response to fungus; regulation of defense response;  negative regulation of transcription;  response to chitin, molecule of bacterial origin, salicylic acid; response to wounding. |  |
| WRKY | + 1711 **(0.85)**  - 1716 **(0.85)** | WRKY42 | *Arabidopsis thaliana* | Negative regulation of transcription. |  |
| WRKY | + 311 **(0.88)**  - 311  + 1720 **(0.89)** | WRKY45 | *Arabidopsis thaliana* | Phosphate ion transport. |  |
| WRKY | - 1721 (**0.88**) | WRKY46 | *Arabidopsis thaliana* | Brassinosteroid mediated signaling pathway; cellular response to hypoxia; defense response to bacterium; lateral root development; regulation of brassinosteroid mediated signaling pathway; regulation of jasmonic acid mediated signaling pathway; regulation of response to water deprivation; regulation of transcription; response to chitin, salicylic acid, bacterium. |  |
| WRKY | + 311 **(0.91)**  - 311 **(0.96)** | WRKY50 | *Arabidopsis thaliana* | Defense response to fungus;  jasmonic acid mediated signaling pathway. |  |
| WRKY | - 311 (**0.98**) | WRKY57 | *Arabidopsis thaliana* | Response to osmotic stress, salt stress, water deprivation |  |
| WRKY | - 311 **(0.9)**  - 1721 **(0.84)** | WRKY71 | *Arabidopsis thaliana* | Positive regulation of transcription, DNA-templated;  vegetative to reproductive phase transition of meristem. |  |
| WRKY | + 309 **(0.88)**  - 309 **(0.89)**  - 312 (**0.98**) | WRKY75 | *Arabidopsis thaliana* | Atrichoblast differentiation; lateral root development; negative regulation of transcription by RNA polymerase II; regulation of transcription in response to stress; regulation of response to nutrient levels |  |
| ZF-HD | - 1054 (**0.97**)  + 1055 (**0.97**)  - 1113 (**0.98**)  + 1114 (**0.97**)  + 1910 (**0.98**)  - 1911 (**1**) | ZHD1 | *Arabidopsis thaliana* | Regulation of GA biosynthetic process;  regulation of transcription;  seed maturation. |  |
| ZF-HD | - 1057 **(0.97)**  - 1116 **(0.98)**  - 1912 **(0.98)** | ZHD5 | *Arabidopsis thaliana* | ABA-activated signaling pathway;  positive regulation of transcription;  response to ABA. |  |
| ZF-HD | + 1050 **(0.94)**  - 1057 **(0.97)**  + 1109 **(0.96)**  - 1116 **(0.98)**  + 1905 **(0.96)** | ZHD9 | *Arabidopsis thaliana* | Glucosinolate metabolic process. |  |
| ZF-HD | - 1048 (**0.92**)  + 1054 (**0.93**)  - 1056 (**0.98**)  + 1057 (**0.98**)  - 1107 (**0.95**)  + 1113 (**0.93**)  - 1903 (**0.93**)  + 1909 (**0.89**)  - 1911 (**1**) | ZHD10 | *Arabidopsis thaliana* | DNA-templated transcriptional start site selection; gibberellic acid mediated signaling pathway; positive regulation of transcription, DNA-templated;  response to blue light;  response to GA. |  |
| Myb/SANT | + 1143 **(0.88)**  + 1146 **(0.99)** | LUX | *Arabidopsis thaliana* | Circadian rhythm;  positive regulation of circadian rhythm. |  |
| Myb/SANT | + 1143  + 1146 | Homeodomain-like superfamily protein PCL1 | *Arabidopsis thaliana* | Circadian rhythm ;  regulation of gene expression;  regulation of transcription. |  |
| VOZ | + 1719 (**0.91**) | VOZ2 | *Arabidopsis thaliana* | Long-day photoperiodism, flowering; red, far-red light phototransduction; positive regulation of long-day photoperiodism, flowering; positive regulation of transcription;  response to salt stress. |  |
| Dehydrin | - 2 (**0.80**)  - 50 (**0.80**)  - 163 (**0.80**)  - 166 (**0.80**)  - 186 (**0.80**)  + 323 (**0.80**)  - 511 (**0.80**)  - 520 (**0.80**)  + 589 (**0.80**)  - 911 (**0.80**)  - 934 (**0.80**)  - 948 (**0.80**)  - 1192 (**0.80**)  - 1203 (**0.80**)  - 1239 (**0.80**)  + 1280 (**0.80**)  - 1396 (**0.80**)  - 1407 (**0.80**)  + 1409 (**0.80**)  - 1414 (**0.80**)  + 1423 (**0.80**)  + 1540 (**0.80**)  - 1593 (**0.80**)  - 1637 (**0.80**)  - 1695 (**0.80**)  - 1831 (**0.80**)  - 1955 (**0.80**) | Protein COLD-REGULATED 15A, chloroplastic | *Arabidopsis thaliana* | Circadian rhythm; cold acclimation; drought recovery; heat acclimation; leaf senescence; plastid inner membrane organization; protein complex oligomerization; protein homo-oligomerization; protein stabilization; red or far-red light signaling pathway; response to abscisic acid, cold, freezing, high light intensity, light stimulus; osmotic stress, salt stress, water deprivation.  Exhibits cryoprotective activity toward stromal substrates (e.g. LDH and rubisco) in chloroplasts and in protoplasts and confers freezing tolerance to plants in a CBF-dependent manner Protectant against various stresses (e.g. cold, drought and heat stress) by preventing protein aggregation (e.g. LDH) and attenuating enzyme inactivation. Influences the intrinsic curvature of the inner membrane of the chloroplast envelope and modulates the freeze-induced lamellar-to-hexagonal II phase transitions that occur in regions where the plasma membrane is brought into close apposition with the chloroplast envelope during freeze-induced osmotic contraction. Mediates a shift in the melting curves of phospholipids-containing membranes to lower temperatures. Involved in the regulation of leaf senescence by ABA in a VNI2-dependent manner. |  |
| bHLH | +/- 793 **(0.83)**  +/- 1095  **(0.83)** | PIF3 | *Arabidopsis thaliana* | De-etiolation; GA_3_-mediated signaling pathway; positive regulation of anthocyanin metabolic process; red, far-red light phototransduction; red or far-red light signaling pathway; regulation of transcription; response to red or far red light. Transcription factor acting positively in the phytochrome signaling pathway. Activates transcription by binding to the G box (5'-CACGTG-3'). |  |
| bHLH | +/- 793 **(0.83)**  +/- 1095  **(0.83)** | PIF1 | *Arabidopsis thaliana* | Chlorophyll biosynthetic process; GA_3_-mediated signaling pathway; heme biosynthetic process; negative gravitropism; negative regulation of photomorphogenesis, seed germination; red light signaling pathway; regulation of photomorphogenesis, seed germination, transcription. Transcription activator. Negatively regulates chlorophyll biosynthesis and seed germination in the dark; light induced degradation of PIF1 relieves negative regulation to promote photomorphogenesis. Binds to the G-box motif (5'-CACGTG-3') found in many light-regulated promoters. Promotes the expression of SOM, and thus modulates responses to ABA and GA_3_. |  |
| SPHCORE,  ZMC1 | - 1138 **(1)** | VP1 | *Zea mays* | Core of Sph element; Core motif of Sph element in maize (Z.m.) C1 gene to which maize VP1 (viviparous 1) protein binds; VP1-responsive element; see RYREPEAT4 (S000010); VP1 gene is specifically required for expression of the maturation program in seed development; VP1 is a novel transcription factor possibly involved in potentiation of a seed-specific hormone response; |  |
| RY element | - 308 (**0.88**)  + 452 (**0.88**) | ABI3 | *Brassica napus; Arabidopsis thaliana* | Required for seed specific expression and ABA responsiveness; dist B ABRE mediated transactivation by ABI3 and ABI3-dependent response to ABA; a tetramer of the composite RY/G complex mediated only ABA-independent transactivation by ABI3; B2 domain of ABI3 is necessary for ABA-independent and ABA-dependent activation through the dist B ABRE |  |
| MYB | + 1207 **(0.86)** | MYB1LEPR | *Arabidopsis thaliana; Lycopersicon esculentum* | Tomato Pti4(ERF) regulates defense-related gene expression via GCC box and non-GCC box cis elements (Myb1(GTTAGTT), G box (CACGTG)). |  |
| MYB-AT-RD22 | - 836  + 1111  - 1205 | MYB2 | *Arabidopsis thaliana* | Binding site for MYB (ATMYB2) in dehydration-responsive gene, rd22; MYB binding site in rd22 gene of Arabidopsis thaliana; ABA-induction; |  |
| Core of CAN element | + 452 | CANBNNAPA | *Brassica napus* | Core of "(CA)n element" in storage protein genes in Brassica napus; embryo- and endosperm-specific transcription of napin (storage protein) gene, napA; seed specificity; activator and repressor. |  |
| LEAFY-AT-AG | + 500  - 879  + 1625 | LEAFY | *Oryza sativa; Arabidopsis thaliana* | Target sequence of LEAFY in the intron of AGAMOUS gene in Arabidopsis. |  |
| MYB2  CONSENSUS | + 35 **(1)**  + 48 **(1)**  +/- 188 **(1)**  + 317 **(1)**  +/- 458 **(1)**  - 590 **(1)**  - 816 **(1)**  + 936 **(1)**  +/- 1002 **(1)**  +/- 1071 **(1)**  + 1088 **(1)**  + 1190 **(1)**  + 1829 **(1)**  + 1853 **(1)**  + 1957 **(1)** | MYB2 | *Arabidopsis thaliana* | Cellular response to phosphate starvation; positive regulation of transcription; regulation of transcription.  Response to ABA, cadmium ion, ethylene, salicylic acid, salt stress, water deprivation.  MYB recognition site found in the promoters of the dehydration-responsive gene rd22 and many other genes in Arabidopsis. |  |
| MYB1-AT |  | MYB1 | *Arabidopsis thaliana* | MYB recognition site found in the promoters of the dehydration-responsive gene rd22 and many other genes in Arabidopsis. |  |
| GT1  CONSENSUS | + 15 (**1**)  - 221 (**1**)  - 258 (**1**)  - 259 (**1**)  + 357 (**1**)  - 904 (**1**)  + 983 (**1**)  + 1215 (**1**)  - 1288 (**1**)  - 1289 (**1**)  + 1521 (**1**)  - 1536 (**1**)  - 1612 (**1**)  + 1652 (**1**)  + 1819 (**1**)  - 1824 (**1**) | GT-1 | *Pisum sativum; Avena sativa; Oryza sativa; Nicotiana tabacum; Arabidopsis thaliana; Spinacia oleracea; Fabaceae (Family)* | Consensus GT-1 binding site in many light-regulated genes, e.g., RBCS from many species, PHYA from oat and rice, spinach RCA and PETA, and bean CHS15. GT-1 can stabilize the TFIIA-TBP-DNA (TATA box) complex. The activation mechanism of GT-1 may be achieved through direct interaction between TFIIA and GT-1. Binding of GT-1-like factors to the PR-1a promoter influences the level of SA-inducible gene expression; |  |
| GT1-GMSCAM4 | - 258 **(1)**  + 1215 **(1)**  - 1288 **(1)**  + 1652 **(1)** | GT-1 | *Glycine max* | GT-1 motif found in the promoter of Glycine max CaM isoform, SCaM-4; Plays a role in pathogen- and salt-induced SCaM-4 gene expression; (GT-1 consensus). |  |
| LRE-BOXI-PCCHS1 | + 1113 **(0.7)**  - 1487 **(0.8)**  - 1709 | MYB1 | *Petroselinum crispum* | BoxI; Light responsive element found in the parsley CHS-1 (chalcone synthase-1) gene promoter. Required for light responsiveness; nuclear protein binding site. Recognition site of MYB305 and a novel factor PcMYB1. |  |
| WINPSTPIIIK |  | WINPSTPIIIK | *Solanum tuberosum* | Binding site of wound-inducible nuclear protein from wounded potato leaves. Found in the promoter region of a protease inhibitor IIK gene from potato. |  |
| L4DCPAL1 | + 119 | n/a | *Daucus carota* | L4 element, found in PAL1 promoter in Daucus carota,is a UV-B responsive element; L4 contains Box L-like sequence (TCCAACCA); see also S000492 (BoxL core of DC PAL); |  |
| RY-REPEAT4 | + 487 **(0.77)** | VP1 | *Zea mays; Triticum aestivum; Oryza sativa* | Seed expression; ABA; VP1 gene is specifically required for expression of the maturation program in seed development. |  |
| PRE-AT-  PRODH | + 1846 **(1)** |  | *Arabidopsis thaliana* | PRE (Pro- or hypoosmolarity-responsive element) found in them promoter region of proline dehydrogenase (ProDH) gene in Arabidopsis; Core of 9-bp sequence ACTCATCCT which is necessary for the efficient expression of ProDH in response to L-Pro and hypoosmolarity. ATB2-binding site. |  |
| LTRE1-HV-BLT49 | - 519  - 933 | unknown | *Hordeum vulgare* | LTRE-1 (low-temperature-responsive element) in blt4.9 gene promoter; A new LTRE; A previously known LTRE is CCGAC; |  |
| PYRIMIDINE BOX; MYB;  Myb/SANT; trp; NF-YC | + 551 (**0.86**)  - 1446 (**0.86**)  - 1665 (**0.86**)  - 1944 (**0.86**) | NF-YC9 | *Oryza sativa; Hordeum vulgare;*  *Arabidopsis thaliana* | ABA-activated signaling; GA_3_-mediated signaling pathway;  positive regulation of photomorphogenesis;  regulation of seed germination; regulation of transcription. |  |
| Myb/SANT; trp; MYB;  NF-YC | + 551 (**0.86**)  - 1446 (**0.86**)  - 1665 (**0.86**)  - 1944 (**0.86**) | CDC5 | *Arabidopsis thaliana* | Cell cycle; defense response signaling pathway, resistance gene-dependent; defense response signaling pathway, resistance gene-independent; defense responses to bacteria and fungi; DNA repair; innate immune response;  mRNA processing; regulation of transcription; RNA splicing |  |
| Myb/SANT; MYB | + 273 **(0.86)**  - 1269 **(0.86)** | GAMYB | *Oryza sativa* | Anther and pollen development; cell differentiation. |  |
| Alpha-amylase | - 34  + 273  - 316  + 497  - 1193  - 1269 | Alpha-amylase | *Oryza sativa subsp. japonica* | Starch catabolic process; sucrose catabolic process  Important for breakdown of endosperm starch during germination. Pyrimidine box found in rice alpha-amylase (RAmy1A) gene. Partially involved in sugar repression. Found in the promoter of barley alpha-amylase (Amy2/32b) gene which is induced in the aleurone layers in response to GA. BPBF protein binds specifically to this site. |  |
| MYB-related | - 262 **(0.86)**  + 829 **(0.86)**  + 1234 **(0.86)**  + 1349 **(1)** | Single myb histone 1  SMH1 | *Zea mays* | Nucleosome assembly. |  |
| bHLH | - 360 **(0.75)**  - 767 **(0.75)**  + 793 **(1)**  + 1095 **(1)**  + 1197 **(0.75)**  - 1199 **(0.75)** | MYC2 | *Arabidopsis thaliana* | ABA-activated signaling pathway; extracellular ATP signaling; positive regulation of flavonoid biosynthetic process; positive regulation of transcription; protein homo-tetramerization; regulation of defense response to insect; regulation of DNA-binding transcription factor activity; regulation of secondary cell wall biogenesis; regulation of transcription from RNA polymerase II promoter in response to oxidative stress; response to ABA, chitin, desiccation, jasmonic acid, wounding; stomatal complex development. |  |
| LEA_5;  RY-REPEAT-VFLEB4 | - 91 (**0.75**)  - 477 (**0.75**)  + 481 (**0.88**)  - 894 (**0.88**)  - 1137 (**0.88**)  - 1222 (**0.88**)  + 1497 (**0.75**)  + 1546 (**0.75**) | Embryonic abundant protein 1 | *Oryza sativa subsp. japonica* | Response to abscisic acid. Em protein may act as a cytoplasm protectant during desiccation.  Binding site of Arabidopsis B3-domain-containing transcription factor FUS3; TRAB1, bZIP transcription factor, interacts with VP1 and mediates abscisic acid-induced transcription; FUS3 protein physically interact with two RY elements present in the AtGA3ox promoter. |  |
| Homeo-domain;  HD-ZIP; HD-ZIP | + 359 (**1**)  - 408 (**0.86**)  - 615 (**1**)  - 718 (**1**)  + 1063 (**0.86**) | MERISTEM L1 | *Arabidopsis thaliana* | Cotyledon development; plant epidermal cell differentiation; seed germination. |  |
| BOX1-PV-CHS15 | + 265 **(0.71)**  + 329 **(0.79)**  - 436 **(0.71)**  - 447 **(0.79)**  - 719 **(0.71)**  - 775 **(0.71)** | SBF-1 | *Phaseolus vulgaris* | Box 1 of bean chs15 promoter; one of SBF-1 binding sites in chs15 promoter. Involved in organ-specific expression in plant development. Functions as a transcriptional silencer in electroporated protoplasts derived from undifferentiated suspension-cultured soybean cells. Resembles the binding site for the GT-1 factor in light-responsive elements. |  |
| bHLH | +/- 388 **(1)**  +/- 435 **(1)**  +/- 635 **(1)**  +/- 714 **(1)**  +/- 793 **(1)**  +/- 816 **(1)**  +/- 1095 **(1)**  +/- 1840 **(1)**  +/- 1853 **(1)** | BIM1 | *Arabidopsis thaliana* | Positive brassinosteroid-signaling protein.  Transcription factor that binds specifically to the DNA sequence 5'-CANNTG-3'(E box).  Can bind individually to the promoter as a homodimer or synergistically as a heterodimer with BZR2/BES1.  Does not itself activate transcription but enhances BZR2/BES1-mediated target gene activation. |  |
| (Others) | +/- 321 **(0.83)**  +/- 621 **(0.83)**  +/- 1322  **(0.83)**  +/- 1397  **(0.83)**  +/- 1600  **(0.83)**  +/- 1728  **(0.83)**  +/- 1938  **(0.83)** | RISBZ3 | *Oryza sativa* | Transcriptional activator that possesses broad binding specificity for DNA promoter elements with the core sequence 5'-ACGT-3'.  May be involved in the regulation of genes expressed during seed development.  Binds to the DNA specific sequence 5'-TGAGTCA-3' found in seed storage protein gene promoters. |  |
| (Others) | - 519 **(0.86)**  - 933 **(0.86)**  - 1395 **(0.86)**  - 1830 **(0.86)** | RD29A (Low-temperature-induced protein) (Desiccation-responsive protein 29A) | *Arabidopsis thaliana* | Circadian rhythm; hyperosmotic salinity response; leaf senescence; regulation of root development; response to ABA, cold, mannitol, osmotic stress, reactive oxygen species, salt, salt stress, symbiotic bacterium, water deprivation, wounding. Involved in responses to abiotic stresses. Probably regulates root elongation in cold conditions. |  |

**Supplemental Table S2.** List of primers utilized in PCR, RT-PCR, RACE-PCR, genome walking, and transgenic plant gene expression.

| Gene | Primer Name | Forward (5’→3’) | Reverse (5’→3’) |
| --- | --- | --- | --- |
| **RT-PCR for isolation of expressed and conserved *LEC1* sequence from Douglas-fir** | | | |
| Pinus cDNA |  | 5’-AGAGAGCAAGATAGGTTCATGCC-3’ |  |
| AtLEC1 |  |  | 5’-CCMARCTTGCTCATAGCCCARAG-3’ |
| **RACE-PCR** | | | |
|  | GSP1  5’ RACE |  | 5’-CTGACAACGTTCATTGGCCTCACTGG-3’ |
|  | GSP2  3’ RACE | 5’-CCCACCCATGCAAAGATTTCTGATG-3’ |  |
| PmLEC1 | PmLEC1 FWD & REV | 5’-ATGATGTCCGAAGTTGGAAGCCCT-3’ | 5’-CTTATACTGAGCATAGGGATCATA-3’ |
| **Genome walking for isolation of genomic DNA sequence upstream of the coding region** | | | |
| PmLEC1 | GSP1 |  | 5’-CTGACAACGTTCATTGGCCTCACTGG-3’ |
| PmLEC1 | GSP2 |  | 5’-CTTCCAACTTCGGACATCATAC-CTAC-3’ |
| **PCR for confirming genotype and analyzing gene expression of transgenic plants** | | | |
| AtLEC1 | AtLEC1 FWD & REV | 5’-ATGACCAGCTCAGTCATAG-3’ | 5’-TCACTTATACTGACCATAATG-3’ |
| PmLEC1 | PmLEC1 FWD & REV | 5’-ATGATGTCCGAAGTTGGAAGCCCT-3’ | 5’-CTTATACTGAGCATAGGGATCATA-3’ |
| Oleosin | Oleosin  FWD & REV | 5’-ATGGCCGATACAGCTAGAGG-3’ | 5’-AGAGAAAACGGTTATAGCGGC-3’ |
| Cruciferin | Cruciferin  FWD & REV | 5’-ATGGTGCTTCCTAAATACAAG-3’ | 5’-TTAAGCCTCGACAATCTCCT-3’ |
